# Analyzing the Evolution and Host Adaptation of the Rabies Virus from the Perspective of Codon Usage Bias

**DOI:** 10.1155/2023/4667253

**Published:** 2023-10-10

**Authors:** Gen Li, Xuhong Chen, Xin Li, Yinyi Liang, Xiaolong Li, Weiheng Liang, Zhibin Yan, Yueming Wang, Yang Wang, Jun Luo, Xiao-Feng Guo, Xiu-Tong Zhu

**Affiliations:** ^1^College of Veterinary Medicine, South China Agricultural University, Guangzhou 510642, China; ^2^South China Biological Medicine, Guangzhou 511300, China

## Abstract

Rabies virus (RABV) is a highly pathogenic virus that causes a fatal disease in humans and other mammals, but the mechanism of its evolution, spread, and spillover remains unknown. In this study, we analyzed the codon usage pattern of 2,018 RABV full-length genome sequences from 79 countries collected between 1931 and 2021 to provide an insight into its molecular evolution and unravel its unknown host-adapted pattern. We found that RABV exhibited a weak codon usage bias, with a preference for the codons ending in A (28.10 ± 0.01) or U (26.43 ± 0.02). Moreover, natural selection plays a major role in shaping the codon usage bias of the RABV. Notably, nearly half of the 18 codons in the virus were best matched to the hosts' most abundant isoacceptor tRNAs, which might account for the wide range of RABV hosts. Furthermore, significant differences were observed in the codon usage patterns of RABV for different host species, suggesting that codon usage bias may be influenced by host-specific factors. In conclusion, our study reveals codon usage patterns of RABV that may help in the development of control strategies and effective vaccines and therapies against this deadly virus.

## 1. Introduction

Rabies is a fatal zoonotic disease of almost all warmblooded animals, including humans. It is caused by the rabies virus (RABV), which is responsible for the deaths of more than 59,000 people annually [1, 2]. The disease is particularly prevalent in developing countries, primarily in Asia and Africa, where they account for approximately 99% of human cases [3–6]. This virus poses a persistent and significant health threat to the public. To this end, international organizations such as the World Health Organization (WHO), the World Organization for Animal Health (WOAH), and the Global Alliance for Rabies Control (GARC) are working together to support countries to eliminate dog-mediated rabies by 2030 (https://www.who.int/news-room/commentaries/detail/new-global-strategic-plan-to-eliminate-dog-mediated-rabies-by-2030).

RABV is a negative-sense and single-stranded RNA virus with an envelope and bullet-shaped morphology [2]. It belongs to the genus *Lyssavirus* of family *Rhabdoviridae* and has a genome size of approximately 12 kb (https://ictv.global/report/chapter/rhabdoviridae/rhabdoviridae). The genome is composed of five proteins, nucleoprotein (N), phosphoprotein (P), matrix protein (M), glycoprotein (G), and polymerase (L) from the 3′ terminal to the 5′ terminal [2, 7]. Based on the previous studies, RABV can be divided into eight clades, including Africa-2, Africa-3, Arctic-related, Asian, Bats, Cosmopolitan, Indian-sub, and RAC-SK (Raccoons and skunks) [8–10]. Of these clade, Africa-2, Africa-3, Arctic-related, Asian, Cosmopolitan, and Indian-sub are primarily associated with hosts within the *Canidae* family, including almost all human cases, which are also termed dog-related clade. Therefore, RABV can be also divided into three major clades depending on its host species, dog-related, bat-related, and RAC&SK-related [10–12]. However, the mechanisms of viral spillover and cross-species transmission are not yet definite.

Codon usage bias is the nonrandom usage among the synonymous codons [13–16]. Under environmental pressure, viruses prefer synonymous codons in order to adapt to change, given the degeneracy of the codon [17, 18]. While viruses replicate inside a host organism, they rely on the cellular resources and energy of the host to carry out the processes of replication, translation, and expression [19, 20]. Thus, as viruses switch between different hosts, their codon usage pattern would change to increasing their translation efficiency and overall replication rate for new cellular environment. Meanwhile, evasion of the host immune response and spillover events could result from the change in viral codon usage bias [21, 22]. In the study of SARS-CoV-2 codons, it was observed that the expression levels of host genes with a similar pattern of synonymous codon usage to the virus significantly decreased. This could be attributed to competition for resources between the virus and the host, leading to the reduction in the expression of host genes that share similar codons, potentially facilitating immune evasion [23]. Furthermore, through the analysis of codon usage patterns in virus-infected yeast cells and human cells, it was found that the similarity in codon usage between the virus and the host influences their coevolution [24]. Therefore, it is essential to understand into the molecular evolution and host adaptability of viruses by studying the viral codon usage bias [24–27].

RABV is an ancient virus that has been extensively studied to elucidate its genomic features and transmission, evolutionary dynamics, and epidemiology [8–10, 28]. However, analysis on codon usage and adaptation to hosts of RABVs were scattered. To fill the gap, we used the latest and most comprehensive RABV genomes to analyze viral base composition, factors affecting codon usage, and adaptation to hosts. This study provides novel insights into the evolution, spread, and spillover of RABV from the perspective of the viral codon usage.

## 2. Materials and Methods

### 2.1. Sequence Dataset

In this study, full-length genome sequences of RABV, including their coding genes, were obtained from RABV-GLUE, a sequence data resourcefor RABV (http://rabv-glue.cvr.gla.ac.uk/) [10]. The dataset was accessed on March 15, 2023, at the time of conducting this study. Sequences without information on collection countries or collection years, or with a length below 11,000 bp were excluded. A total of 2,018 sequences collected from 79 countries between 1931 and 2021 and from a wide variety of host species were analyzed. Details of the sequences are provided in Table S1 (*Supplementary 1*). Annotated coding sequences of the *Canis lupus familiaris* genome (Dog10K_Boxer_Tasha), *Homo sapiens* genome (GRCh38.p13), *Mustela erminea* genome (mMusErm1.Pri), and *Myotis lucifugus* genome (Myoluc2.0) were obtained from NCBI GenBank [29–31]. The isoacceptor tRNAs in Canis familiaris, *Homo sapiens*, *Myotis lucifugus*, and *Mustela putorius* furo were retrieved from the Genomic tRNA Database (GtRNAdb, http://gtrnadb.ucsc.edu/GtRNAdb2/index.html) [32].

### 2.2. Distribution and Phylogenetic Analysis of Genomic Sequences

The original sequences were aligned with the MAFFT software (version 7.313, mafft–auto input-file > output-file) and trimmed with the trimAI software (version 1.2, trimal -in alignment-file -out trimAI-file -automated1) [33, 34]. The maximum-likelihood (ML) tree was reconstructed using the IQ-Tree software (version 1.6.8) with a total of 1,000 bootstraps (iqtree2 -s trimAI-file -o “outgroup-name” -b 1000 - T auto) [35, 36]. The best-fitting nucleotide substitution model, the general time-reversible substitution model with empirical base frequencies, and relaxing gamma-distributed rate heterogeneity (GTR + F + R10, automatically selected), were selected using the ModelFinder software (version 1.6.8, one module in IQ-tree) [37]. The final tree was visualized in iTOL [38] (Interactive Tree Of Life, https://itol.embl.de/).

### 2.3. Nucleotide Components Analysis

The nucleotide composition properties of RABV coding sequences were analyzed, including the frequency of all nucleotides (GC%, AU%, A%, U%, G%, and C%) and the A, C, G, and U frequencies in synonymous codons at different sites (GC1%, GC2%, GC3%, GC12%, A3%, U3%, G3%, C3%, and AU3%). These values were calculated using the CAIcai tool (http://genomes.urv.es/CAIcal), Codon W software (version 1.4.2, codonw alignment-file out-file blk-file -code 0 -silent -totals -all_indices -nomenu) [39], and the Seqinr package (version 4.2-23, http://mirrors.cqu.edu.cn/CRAN/web/packages/seqinr/index.html) in R [40].

### 2.4. Relative Synonymous Codon Usage (RSCU) Analysis

RSCU is a measure of the degree of codon usage bias in different genes or species [41]. RSCU values were calculated using the Seqinr package (version 4.2-23) in R. The RSCU value is calculated as follows:

(1) $$\begin{array}{c} \text{RSCU}=\frac{g_{\text{ij}}}{\sum_{j}^{\text{ni}}g_{\text{ij}}}\text{ni }. \end{array}$$

If the RSCU values >1.6, the codon is considered to be overrepresented, while values <0.6 indicate underrepresentation. A value of 1.0 suggests no codon usage bias.

### 2.5. Effective Number of Codons (ENC) Analysis

ENC refers to the number of valid codons used in a gene, indicating that the random selection of codon usage deviation [42]. The ENC values range from 20 to 61, with values closer to 20 representing stronger codon preference. The following equation was applied to calculate the ENC:

(2) $$\begin{array}{c} \text{ENC}=2+\frac{9}{{\overset{―}{F}}_{2}}+\frac{1}{{\overset{―}{F}}_{3}}+\frac{5}{{\overset{―}{F}}_{4}}+\frac{3}{{\overset{―}{F}}_{6}}\;, \end{array}$$

*F* (*i* = 2, 3, 4, 6) stands for Fi values for the *i*-fold degenerate amino acids. ENC values were calculated using the coRdon package (version 1.16.0) in R.

### 2.6. Analysis of ENC-Plot, Parity Rule 2 (PR2), and Neutrality Plot

ENC-plot refers to the correlation between ENC and GC3s to investigate the major factors affecting codon usage bias in different genes or species [43, 44]. The following formula was used to calculated the expected ENC values:

(3) $$\begin{array}{c} \text{ENC expected}=2+s+\frac{29}{s^{2}+\left(1-s^{2}\right)}\;. \end{array}$$

If the codons are only under the pressure from G + C mutational bias, the ENC-GC3s points will fall on or around the expected curve, whereas, the points away from the expected curve indicate that the codons are also under the pressure from natural selection.

PR2 analysis is a method for evaluating the effects of random mutational pressure and natural selection on codon usage patterns. The plot consists of the AU deviation (A3/(A3 + U3)) as the vertical coordinate and the GC deviation (G3/(G3 + C3)) as the horizontal coordinate, with a plotting origin of 0.5. The direction and extent of codon usage bias is inferred from the quadrant in which the points fall and the distance from the origin point (0.5, 0.5), with the origin point implying equal contributions from random mutation pressure and natural selection [45, 46].

Neutrality plot refers to the assessment of the correlation between the GC3s as the horizontal coordinate and GC12s as the vertical coordinate [47]. Mutations occurring at the third position of the codon are mostly synonymous, which are the most neutral bases, whereas mutations in the bases at the first and second positions of the codon cause amino acid changes. Therefore, the ratio of GC3s–GC12s, also known as the regression coefficient, can discern the dominant role of random mutation and natural selection on the codon. A regression coefficient closer to 1 indicates that there is little or no external selective pressure and that random mutation plays a dominant role. The closer the regression coefficient is to zero, or the absence of a significant correlation in the regression curve, the more natural selection prevails.

### 2.7. Correspondence Analysis (COA) and Correlation Analysis

COA is a multivariate statistical analysis that determines variable and sample relationships by reducing the dimensionality of the data and filtering out the main factors. COA was performed from the RSCU values and visualized in the ggplot2 package of R.

The corrplot package of R and GraphPad Prism were applied to measure the correlation between variables.

### 2.8. Relative Dinucleotide Abundance Analysis

Codon usage bias is restrained by the relative abundance of 16 dinucleotides, possibly due to the intrinsic properties of the viruses or to the mutational pressure of the innate immune system of the host. The relative dinucleotides abundances are defined as the ratio of observed to expected dinucleotide frequency, computed by SSE (version 1.4, http://www.virus-evolution.org). The contents of the 16 dinucleotides calculated as the following formula [48]:

(4) $$\begin{array}{c} P_{xy}=\frac{f_{xy}}{f_{x}f_{y}}\;. \end{array}$$

In the formula, *f*_*x*_ and *f*_*y*_ represent the frequency of nucleotide *X* and *Y*, respectively, and the *f*_*xy*_ represents the frequency of the dinucleotide *XY*. When *P*_*xy*_ < 0.78 or *P*_*xy*_ > 1.23, the dinucleotides were inferred as underrepresented or overrepresented, respectively [49].

### 2.9. Adaption Index Analysis between RABV and Hosts

The codon adaption index (CAI) is supposed to investigate the codon usage bias of viral genome or gene across different species, with a range from 0 to 1. Theoretically, a high-CAI value means that the codon usage pattern of a virus is adapted to that of its host [50, 51]. The relative codon deoptimization index (RCDI) is used to compare the similarity of codon usage between virus and host. If the RCDI = 1, the virus is considered to be fully adapted to its host, while the RCDI much higher than 1 indicates poor adaptability [52, 53]. Moreover, the similarity index (SiD) value is used to assess the influence of host codon usage on pathogen codon usage, with the range from 0 to 1. Higher SiD values indicate greater impact of the hosts on the codon usage of the pathogen [54]. The calculation of the SiD value was as follows:

(5) $$\begin{array}{c} R\left(a,b\right)=\frac{\sum_{i=1}^{50}a_{i}\times b_{i}}{\sqrt{\sum_{i=1}^{\infty }a_{i}^{2}\times \sum_{i=1}^{40}b_{i}^{2}}}\;, \end{array}$$

(6) $$\begin{array}{c} D\left(A,B\right)=\frac{1-R\left(A,B\right)}{2}\;. \end{array}$$

To balance the sample bias between the three phylogenetic clades, we used a python script to randomly select 50 sequences from each of the 2,018 RABV coding sequences from bat-related, dog-related, and RAC&SK-related, for a total of 150 sequences for the fitness analysis. All of the CAI, RCDI, and SiD were computed by vhcub package of R [55].

## 3. Result

### 3.1. Distribution and Phylogenetic Tree of Full-Length Genomes

**Figure 1:**
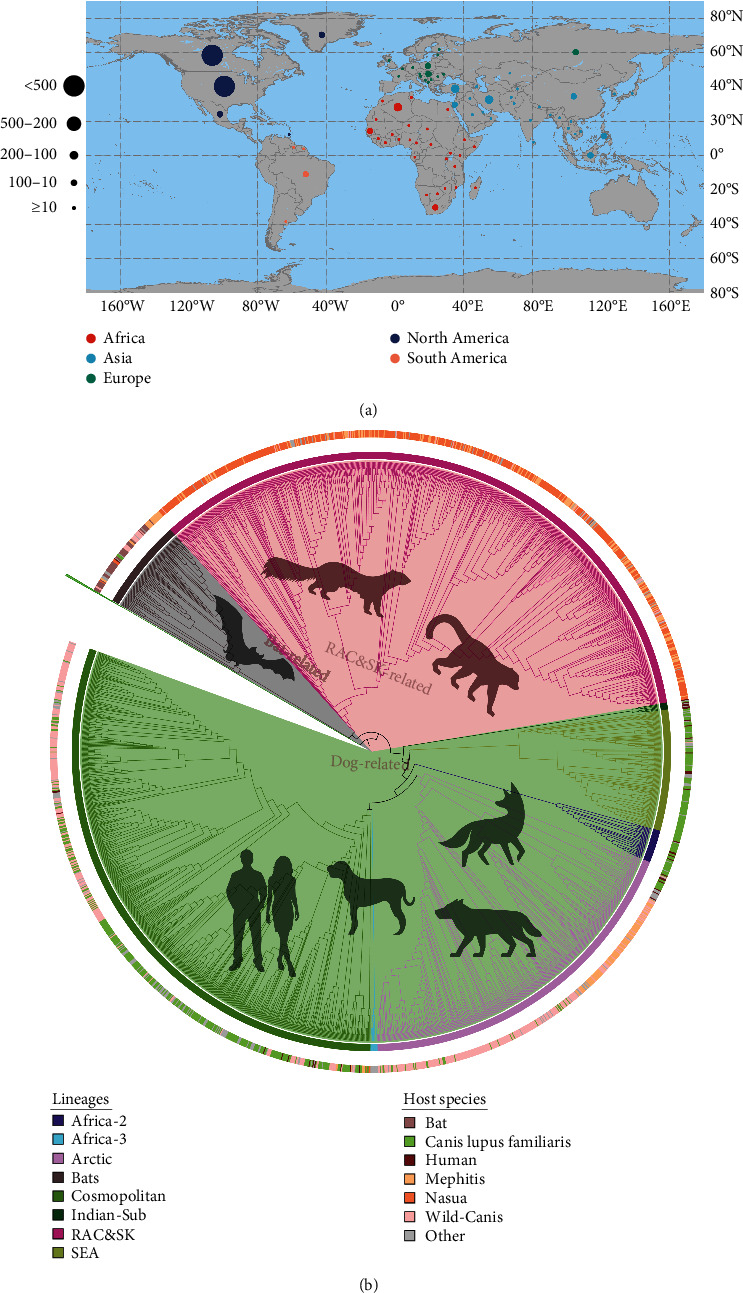
Distribution and phylogenetic tree of full-length genomes. (a) Geographical distribution of RABV genomes around the world. Different continents are denoted by different colors of circle; the number of sequences is denoted by sizes of circle for different country. (b) Phylogenetic tree of RABV genomes. Inner ring stripe represents the lineages in previous study; outer ring stripes represent the different host of RABV.

According to the data from the GenBank database, the RABV full-length genomes have been reported in 79 countries, and the virus can infect virtually at all warmblooded animals, including bat, dog, fox, wolf, skunk, racoon, and even human. There were 1,187 sequences, with the largest number in Nouth America, most of which were sampled from skunks or raccoons. In contrast, South America reported the fewest sequences, with only 50, most of which came from bats. Two hundred twenty-one sequences were reported in Europe, and their hosts were mainly wild Canis. Of note, the sequences infecting with Canis-familiaris and human are concentrated in Asia and Africa (Figure 1(a) and Table S1 (*Supplementary 1*)).

The ML tree indicated that the RABV genomes might be divided into eight clades, corresponding to the previous study. Based on the outer ring (host species) of the ML tree, the sequences could roughly divide into three major group, including bat-related, dog-related, and RAC&SK-related (Figure 1(b), https://doi.org/10.6084/m9.figshare.24077211).

### 3.2. Base Composition Analysis of RABV Full-Length Genomes

A clear trend of AU richness was observed in the RABV genomes by considerable analysis of the base composition of the viral coding sequences (*Supplementary 2*). The mean compositions (%) of the nucleotides A (28.10 ± 0.01) and U (26.43 ± 0.02) were significantly higher than C (21.91 ± 0.02) and G (23.56 ± 0.01) (*P* < 0.01). Comparing the codons at the third position, the mean of A3s (32.71 ± 0.05) and U3s (31.91 ± 0.07) were significantly higher than C3s (30.29 ± 0.05) and G3s (31.06 ± 0.01) (*P* < 0.01). Moreover, the GC3s (47.65 ± 0.06) were also lower than the AU3s. Regarding the RSCU patterns, 3 out of the 4 overrepresented (RSCU >1.6) codons with AU3s (Table 1, *Supplementary 2*).

### 3.3. Codon Usage Bias under Natural Selection Pressure

**Figure 2:**
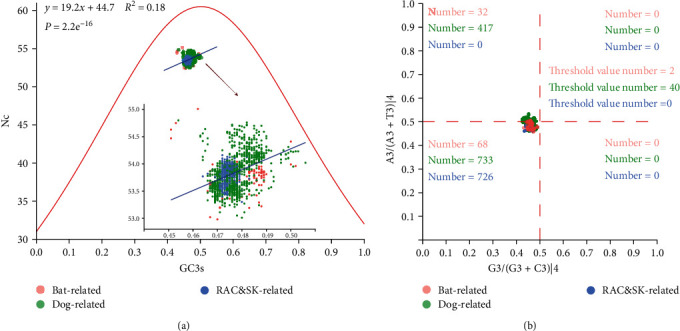
ENC-plot (ENC value plot against GC3s) analysis and parity rule-2 plot analysis of RABV. Different clades are denoted by different colors of circle. (a) ENC-plot (ENC value plot against GC3s) in relation to three major clades and (b) parity rule 2-plot in relation to three major clades.

The ENC-plot revealed that both of random mutational and natural selective pressure on the RABV codon usage bias. We found that ENC value of coding sequences was 53.83 ± 0.28 higher than 35, with a low-codon usage bias, and all the points regardless of group fall under the expected curve and gathered on the left. Furthermore, there was a clear positive correlation between the ENC and GC3s (Figure 2(a)).

To further investigate the codon usage pattern of RABV coding sequences, we used PR2 plot analysis to confirm the effect of random mutation and natural selection on the codon usage bias. Our study found that random mutation and natural selection play unequal roles in the viral codon usage bias, due to the fact that the points are deviated from the origin and concentrated in Quadrants 1 and 3 (Figure 2(b)).

**Figure 3:**
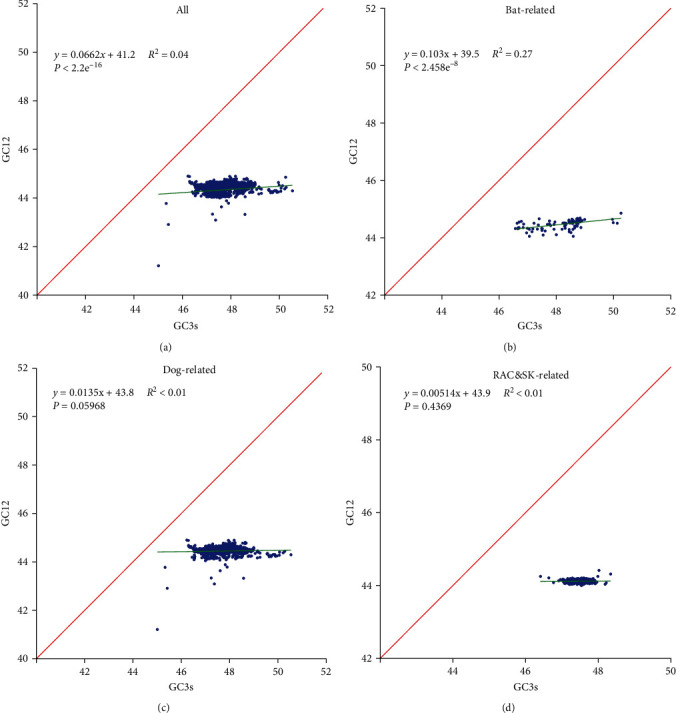
Neutrality plot for RABV and its three clades: (a) all sequences of RABV genome; (b) the genomic sequences of the bat-related; (c) the genomic sequences of the dog-related; and (d) the genomic sequences of the RAC&SK-related.

We then used neutral analysis to show that, compared with random mutations, natural selection pressures play a major role in shaping the codon usage bias of RABV coding sequences. Based on the ML tree analysis, these viral genomes were divided into three phylogenetic groups, and their correlation coefficients between GC3 and GC12 were calculated separately for the bat-related (0.1030), the dog-related (0.0135), and the RAC&SK-related (0.00514). Thus, their percentages of natural selective pressure on codon usage patterns were 89.70%, 98.65%, and 99.49%, respectively. Similarly, natural selection also played an important role in all the ungrouped genomes, contributing 93.38% of the constrain (Figure 3).

**Figure 4:**
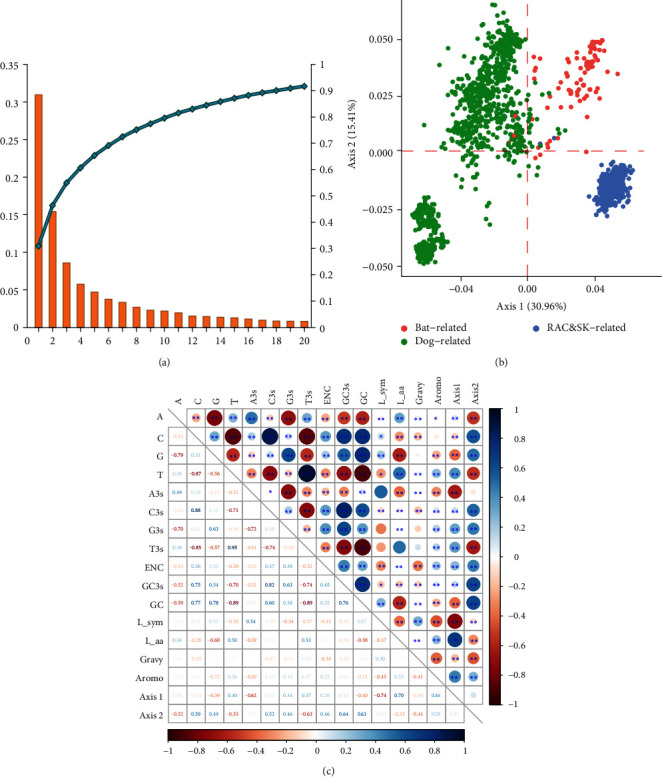
Correspondence analysis and correlation analysis in different nucleotide-relevant indices of RABV. (a) The first 20 axes are applied to display the tendency of codon usage bias of RABV genomes. The plot is drawn based on the relative and cumulative inertia of the first 20 factors; the blue curve with dots represents the cumulative inertia, which is made by the result of COA. (b) Positions of the RABV genomes in the plot of the first two major axes by COA of RSCU values distributed by different countries. (c) Heatmap of correlation analysis in different nucleotide-relevant indices of RABV. Spearman test was performed to test the correlation in different nucleotide-relevant indices.  ^*∗*^*P* < 0.05;  ^*∗∗*^*P* < 0.01; and  ^*∗∗∗*^*P* < 0.001. NS, not significant. Aromo represents aromaticity of the viral gene product; GRAVY represents grand average hydropathicity score. L_sym represents the number of synonymous amino acids; L_aa represents the number of amino acids.

Our results suggest that the host species of RABV is highly correlated with phylogenetic clade and is a potentially critical factor in viral evolution and codon usage bias generation. We applied the first 20 axes to the relative and cumulative inertia analyses and found that the first axis explained 30.96% of the data inertia and was the main driver of variation, with each subsequent axis explaining a decreasing amount of variation. The first two axes accounted for almost half of the total variance, so we restricted our analysis to these two axes. In the COA analysis, these three groups clustered according to their viral host species, suggesting that viral host species can affect codon usage patterns (Figures 4(a) and 4(b)).

In addition, multifactorial correlations were calculated using corrplot package of R. Almost all indices, with the exception of the relationship between T3s and Axis2, were significantly correlated with the main axis, further confirming the above findings. Altogether, the codon usage bias of RABV coding sequences was under the pressure of both natural selection and random mutation (Figure 4(c)).

### 3.4. Comparison of Relative Dinucleotide Abundance of RABV and Its Hosts

**Figure 5:**
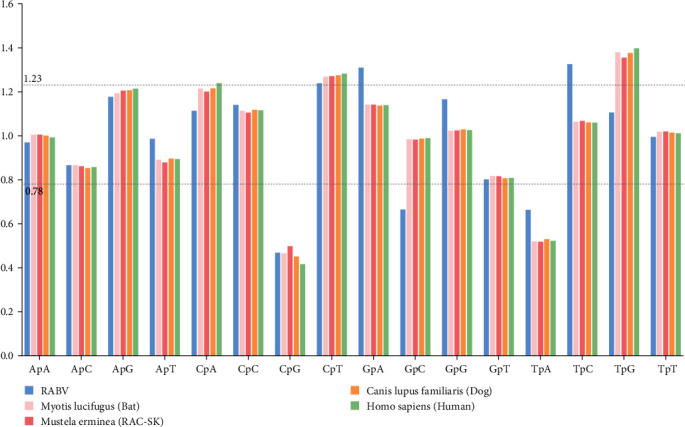
The relative dinucleotide abundance values of 16 dinucleotides of the RABV and its hosts' coding region. Different coding sequences are denoted by different colors.

The relative abundance of 16 dinucleotides were calculated and showed that the frequencies of occurrence of each dinucleotide in the RABV and its host coding region were not evenly distributed. We found that CpU (1.238 ± 0.017), GpA (1.309 ± 0.015) and UpC dinucleotides (1.33 ± 0.014) were overrepresented, which were consistent with that GCU, AGA for Ala, CUG for Leu, CCU for Pro, UCU, UCA, UCC for Ser, ACU for Thr, GAG for Glu, GGA for Gly, and AUC for Ile were preferred in RSCU with the value > 1. While CpG (0.468 ± 0.014), GpC (0.665 ± 0.012), and UpA dinucleotides (0.663 ± 0.013) were underrepresented, which were consistent with that the GCG for Ala, CGC, CGG, CGU for Arg, CCG for Pro, UGG, AGC for Ser, ACG for Thr, UGC for Gly, and UAG for Ter were underrepresented in RSCU with the value < 0.6. Meantime, we noted that the relative dinucleotide abundances of viral hosts were similar. The GpA, TpC, GpC, and TpG showed different degrees between RABVA and its hosts (Table 1 and Figure 5).

### 3.5. Most Preferred Codons in RABV and Isoacceptor tRNAs in Its Hosts

We compared the preferred codons (for each amino acid) in RABV with the most abundant isoacceptor tRNAs in its hosts and found that 9, 8, 9, and 10 of the 18 codons in the virus best matched the most abundant isoacceptor tRNAs in *Myotis lucifugus*, *Mustela putorius furo*, *Canis familiaris*, and *Homo sapiens*, respectively. Among the matched tRNAs, six tRNAs (GTT, CTG, CTT, GAA, AGG, and GTA) corresponding to Asn, Gln, Lys, Phe, Pro, and Tyr were the most abundant in all four hosts (Table 2).

### 3.6. Measures of RABV Adaptation between Different Hosts

In this study, CAI, RCDI, and SiD values were used to assess adaptation of RABV and its three phylogenetic clades, between the virus hosts *Myotis lucifugus* (M.L, represent bat), *Mustela putorius furo* (M.P.F, represent RAC&SK), *Canis familiaris* (C.F, represent dog), and *Homo sapiens* (H.S, represent human). The results of three clades are consistent with all sequences analyzed using CAI, RCDI, and SiD (Table S2 (*Supplementary 1*)).

**Figure 6:**
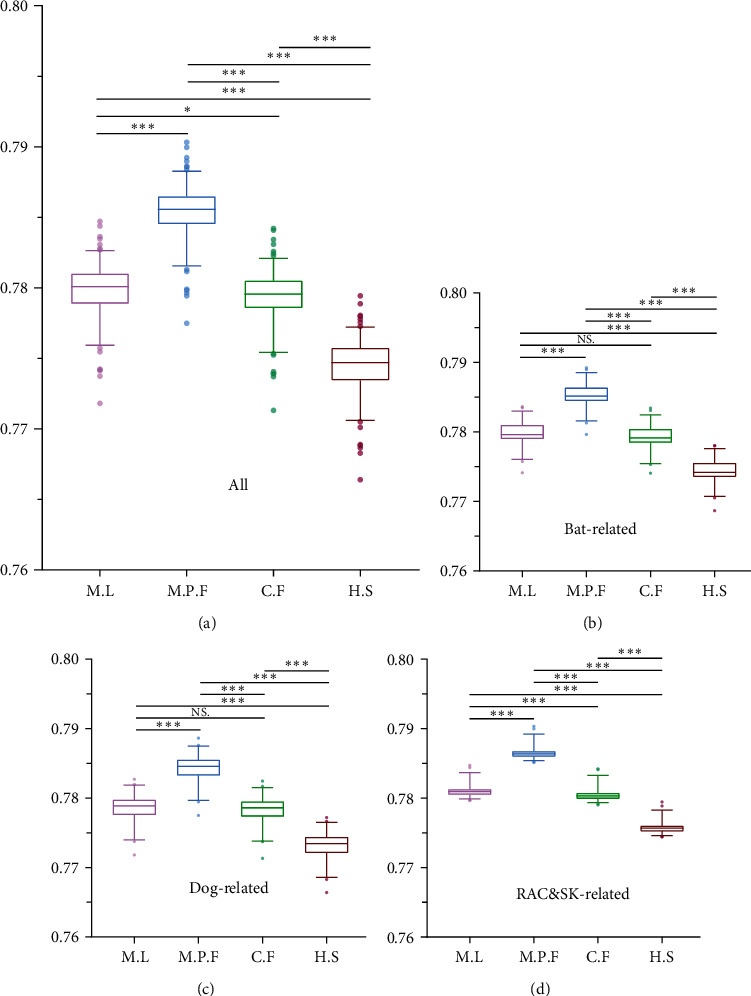
The codon adaption index (CAI) analysis. Different hosts are denoted by different colors: (a) all sequences of RABV genome; (b) the genomic sequences of the bat-related; (c) the genomic sequences of the dog-related; (d) the genomic sequences of the RAC&SK-related. Wilcoxon test was performed to compare the mean of the CAI values pertaining to the different sets of hosts.  ^*∗*^*P* < 0.05,  ^*∗∗*^*P* < 0.01, and  ^*∗∗∗*^*P* < 0.001. NS, not significant.

The CAI values of viral coding genes, calculated from host usage patterns, are commonly used to predict the efficiency of gene expression in different hosts. The average CAI values for RABV with bat, RAC&SK, dog, and human were found to be 0.780 ± 0.002, 0.785 ± 0.002, 0.7793 ± 0.002, and 0.774 ± 0.002, respectively. Based on the CAI analysis, our findings imply that there is a relatively higher adaptation of the RABV, to RAC&SK cellular systems in contrast to bats, dogs, and humans (Figure 6).

**Figure 7:**
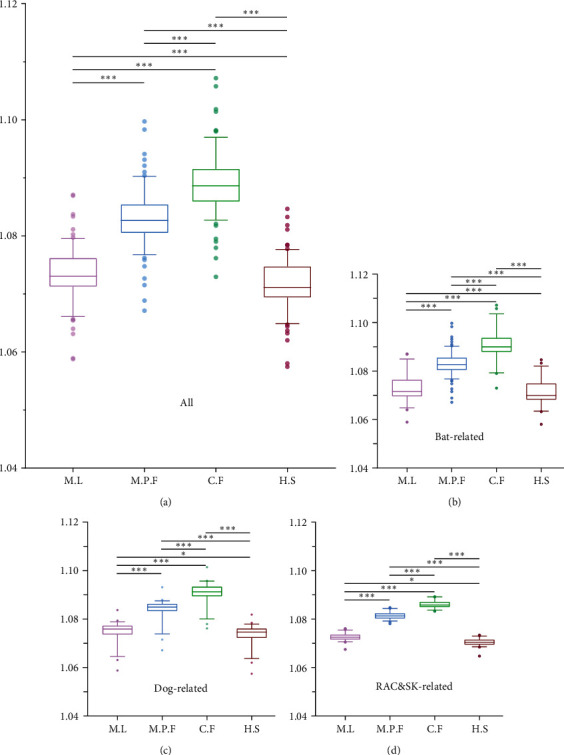
The relative codon deoptimization index (RCDI) analysis. Different hosts are denoted by different colors. (a) All sequences of RABV genome; (b) the genomic sequences of the bat-related; (c) the genomic sequences of the dog-related; (d) the genomic sequences of the RAC&SK-related. Wilcoxon test was performed to compare the mean of the RCDI values pertaining to the different sets of hosts.  ^*∗*^*P* < 0.05;  ^*∗∗*^*P* < 0.01;  ^*∗∗∗*^*P* < 0.001. NS, not significant.

The RCDI value is a measure of the similarity between viral coding genes and its hosts. We calculated the average RCDI values for RABV with bat (1.073 ± 0.004), RAC&SK (1.083 ± 0.004), dog (1.089 ± 0.005), and human (1.072 ± 0.004), respectively. Of note, the human counterpart had the lowest RCDI values compared to the other three hosts, indicating that the genes encoding RABV are the least deoptimised and more adaptive to the human host, which is consistent with the analysis of isoacceptor tRNAs (Table 2 and Figure 7).

**Figure 8:**
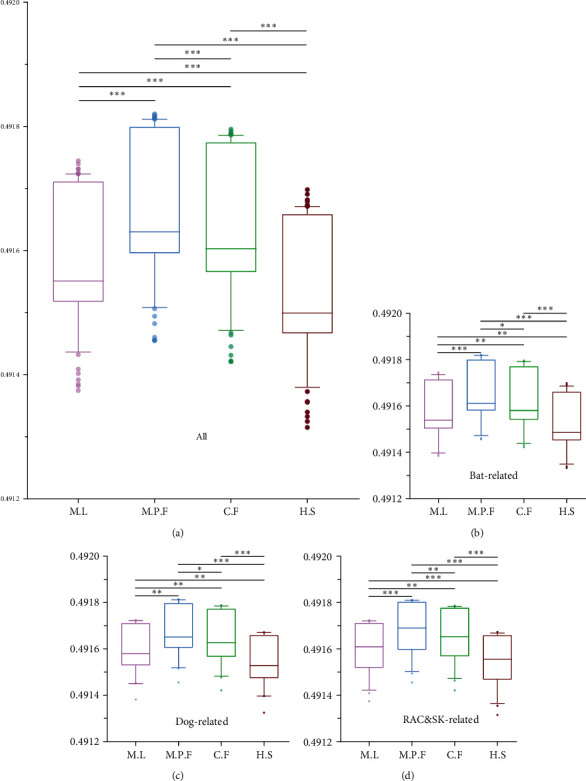
The similarity index (SiD) analysis. Different hosts are denoted by different colors. (a) All sequences of RABV genome; (b) the genomic sequences of the bat-related; (c) the genomic sequences of the dog-related; (d) the genomic sequences of the RAC&SK-related. Wilcoxon test was performed to compare the mean of the SiD values pertaining to the different sets of hosts.  ^*∗*^*P* < 0.05;  ^*∗∗*^*P* < 0.01; and  ^*∗∗∗*^*P* < 0.001. NS, not significant.

The SiD value is applied to assess the effect of the overall codon usage pattern of the viral host on viral codon usage. The average CAI values for RABV with bat, RAC&SK, dog, and human were found to be 0.4916 ± 0.0001, 0.4917 ± 0.0001, 0.4916 ± 0.0001, and 0.4915 ± 0.0001, respectively. This indicates that during RABV evolution, RAC&SK had a greater impact on the virus than other three hosts (Figure 8).

## 4. Discussion

Rabies has a long history, almost accompanying the history of human civilization [1, 2]. It is widely distributed throughout the world and switches repeatedly between the different species [8, 9, 11]. Of note, understanding the ability of RABV to spread across species, particularly across orders, is important for studying viral patterns of cross-species evolution, for predicting the spread of zoonotic infections and thus for their ultimate control. Therefore, our study provides new insights into the evolution, spread, spillover, and adaptation to hosts of RABV from the perspective of the viral codon usage.

The latest and the largest RABV data were used to apply phylogenetic analysis, suggesting the RABV genomes could be divided into three clades, based on the host species, which were similar to the previous reports [8–10]. Subsequently, we searched the GenBank for reference genomes, *Canis lupus familiaris*, *Mustela erminea*, and *Myotis lucifugus* genome as representative hosts for each of the three clades, plus the *Homo sapiens* genome. The base composition analysis of RABV were applied to comprehensive analyze and showed a weak codon usage bias with a preference for the codons ending in A or U, consistent with the RSCU results.

The ENC-plot, PR2-plot, and neutrality-plot analysis were applied to assess the main effect on codon usage bias. For the all sequences, the mean ENC value is 53.83, with a narrow distribution range from 52.98 to 55.01, indicating a low-codon usage bias [56]. Compared to the bat-related and dog-related, the points of RAC&SK-related are more clustered. Furthermore, the points in PR2 plot are concentrated to the left of the origin suggesting that all the sequences have a preference for the codons ending in C rather than G. It is consistent with the ENC plot that he points of RAC&SK-related are more clustered in the Quadrant 3. In term of the neutrality-plot analysis, natural selection pressures play a major role in shaping the codon usage bias of RABV coding sequences, contributing about 90% of the constrain. Of the three clades, the RAC&SK-related has the smallest value of correlation coefficient and *R*^*2*^, and nonsignificant *P* value, indicate that the genome is highly conserved in GC% and under greater pressure from natural selection [24, 27].

In the COA analysis, we find that the first two axes account for almost half of the total variance and the Axis 1 and Axis 2 can divide the RSCU of RABV into three groups, roughly corresponding to the three host species, suggesting viral host species may affect codon usage patterns. Subsequently, a total of 17 nucleotide-relevant indices are used for the correlation analysis. Almost all indices were significantly correlated with the main axis, to further confirming the above results [19, 56].

Most single-stranded RNA viruses are defective in the composition of the CpG and TpA dinucleotide, as has also been observed in RABV in the present study. The low CpG has been reported to contribute to immune evasion. Meanwhile, the CpG and TpA underrepresentation is also a feature of vertebrate genomes. Inhibition of CpG and TpA may be involved in the formation of the codon usage pattern and adaptation to its exclusive hosts. Matching of viral codon usage bias with isoacceptor tRNA abundance determines translation error rates. Of note, about a half of the 18 codons in the virus best are matched to the most abundant isoacceptor tRNAs in the hosts, contributing to rapid adaptation of the virus to the host, accurate expression of viral proteins and improved expression efficiency. It might be a potential factor for the wide host range of RABV [57, 58].

Previous studies have suggested that RABV might originate in the bats and evolved over a long period of time, spreading across order from the order *pteropods* to *carnivores* [8, 11]. In order to compare the differences of RABV adaptation to various hosts, we applied the values of CAI, RCDI, and SiD for assessment. Based on the values of CAI, the adaptation of RABV to each host in descending order is RAC&SK (M.P.F), bat (M.L), dog (C.F), and human (H.S), indicating that the RABV coding sequences have a higher gene expression potential in the RAC&SK and greater adaptation to the RAC&SK. There is a similar trend to the CAI analysis in SiD analysis, suggesting that the host of RAC&SK apply stronger pressure to shape viral codon usage pattern, consistent with the neutrality-plot analysis [59, 60]. Therefore, the RAC&SK might an ideal switching host from the order *pteropods* to *carnivores*, with high adaptation to RABV. However, in term of the RCDI analysis, the value in descending order is dog (C.F), RAC&SK (M.P.F), bat (M.L), and human (H.S), suggesting that RABV is predicted to have a higher translation rate to the host of human, due to the highest number (10/18) of human isoacceptor tRNAs that best match viral preferred codons [57].

RABV employs diverse different strategies to thrive within various host species [61]. When carnivorous animals such as dogs, skunks, and raccoons become infected with the RABV, it often leads to acute death. However, in the case of bats, it may result in long-term carriers of the virus [62, 63]. Additionally, when a host is infected with RABV transmitted by the same species, it typically manifests acute clinical symptoms, eventually leading to fatality. In contrast, infections with viruses from other species tend to yield milder symptoms and may not be lethal [64, 65]. This indicates that the RABV needs to evolve to overcome the host barrier. It also demonstrates that the virus has different adaptation patterns in the hosts of different species, which may be related to the usage of codons.

Although our study provides an overall insight into the RABV codon usage, frankly, this study has several limitations. First, to avoid sampling bias, which is also limited by computational power, we used a Python script to randomly select 50 sequences in each clade, 150 in total, to assess the adaptation of RABV to the hosts. The random selective sequences might not adequately represent the adaptation of the entire clade. Second, our study focuses on only four viral host, bats, dogs, human, and RAC&SK, because these hosts were distinctly clustered and representative on the phylogenetic tree and some hosts' genomes are not available in GenBank.

In summary, our findings provide a novel and comprehensive insight into codon usage bias of RABV and its adaptation to hosts. We reveal natural selection pressure shaping the RABV codon usage pattern, account for more than 90%, and in brief analyzed why RABV has the wide range of hosts and the relation between viral transmission and host adaptation. Overall, the data in the study will help to RABV surveillance and further research to control and eliminate this deadly virus.

## Figures and Tables

**Table 1:** Relative synonymous codon usage patterns of RABV in comparison with its host.

AA	Codon	Virus				Host			
		All-sequences	Bat-related	Dog-related	RAC&SK-related	*Myotis lucifugus*	*Mustela putorius* furo	Canis	*Homo sapiens*
ALA	**GCA**	1.34	1.31	1.31	1.39	1.16	1.1	1.05	1.18
	GCC	1.12	1.11	1.12	1.11	1.23	1.22	1.32	1.22
	*GCG*	0.34	0.49	0.33	0.35	0.48	0.55	0.48	0.44
	GCU	1.2	1.09	1.24	1.15	1.13	1.12	1.15	1.16

ARG	**AGA**	2.76	2.63	2.78	2.73	1.9	1.81	1.86	1.99
	AGG	1.73	1.89	1.67	1.81	1.57	1.36	1.28	1.59
	CGA	0.62	0.53	0.66	0.56	0.61	0.71	0.71	0.6
	CGC	0.27	0.29	0.27	0.28	0.64	0.73	0.77	0.61
	CGG	0.38	0.44	0.39	0.37	0.82	0.87	0.88	0.76
	*CGU*	0.24	0.22	0.23	0.25	0.46	0.53	0.51	0.45

ASN	**AAC**	1.06	1.1	1.11	0.99	1.01	1.01	0.98	0.98
	*AAU*	0.94	0.9	0.89	1.01	0.99	0.99	1.02	1.02

ASP	**GAC**	1.03	0.97	1.01	1.09	1	1.04	1	0.97
	GAU	0.97	1.03	0.99	0.91	1	0.96	1	1.03

CYS	*UGC*	0.77	0.81	0.78	0.75	1.12	1.12	1.05	1.11
	**UGU**	1.23	1.19	1.22	1.25	0.88	0.88	0.95	0.89

GLN	**CAA**	0.97	0.97	0.97	0.96	0.78	0.84	0.79	0.78
	CAG	1.03	1.03	1.03	1.04	1.22	1.16	1.21	1.22

GLU	*GAA*	0.8	0.79	0.81	0.8	1.04	1.05	1.02	1.06
	**GAG**	1.2	1.21	1.19	1.2	0.96	0.95	0.98	0.94

GLY	GGA	1.34	1.38	1.36	1.3	1.36	1.41	1.35	1.38
	*GGC*	0.58	0.58	0.59	0.56	1.06	1.08	1.12	1.06
	**GGG**	1.46	1.41	1.45	1.48	0.93	0.89	0.87	0.91
	GGU	0.62	0.64	0.6	0.65	0.65	0.63	0.66	0.65

HIS	*CAC*	0.87	0.78	0.89	0.85	1.04	1.03	1.01	1.03
	**CAU**	1.13	1.22	1.11	1.15	0.96	0.97	0.99	0.97

ILE	AUA	1.03	1.03	1.03	1.02	0.77	0.72	0.61	0.76
	**AUC**	1.16	1.11	1.17	1.14	1.14	1.2	1.25	1.13
	*AUU*	0.81	0.85	0.79	0.84	1.09	1.07	1.14	1.11

LEU	CUA	0.87	0.77	0.92	0.81	0.59	0.62	0.59	0.61
	CUC	0.92	0.92	0.87	1.01	1.13	1.25	1.23	1.12
	CUG	1.22	1.32	1.22	1.22	1.77	1.72	1.85	1.76
	CUU	0.94	0.98	0.91	0.97	0.99	1.1	1.09	1.02
	*UUA*	0.72	0.56	0.73	0.71	0.58	0.51	0.51	0.58
	**UUG**	1.33	1.45	1.35	1.28	0.93	0.79	0.73	0.92

LYS	*AAA*	0.96	0.93	0.91	1.04	0.98	1	1	1
	**AAG**	1.04	1.07	1.09	0.96	1.02	1	1	1

PHE	**UUC**	1.02	1.03	1.04	1	1.03	1.05	1.01	1.02
	*UUU*	0.98	0.97	0.96	1	0.97	0.95	0.99	0.98
PRO	CCA	0.91	0.86	0.88	0.97	1.27	1.2	1.16	1.31
	CCC	1.05	1.05	1.1	0.98	1.08	1.09	1.13	1.05
	*CCG*	0.58	0.68	0.5	0.68	0.54	0.61	0.54	0.48
	**CCU**	1.46	1.41	1.52	1.38	1.12	1.1	1.17	1.16

SER	AGC	0.56	0.58	0.55	0.57	1.37	1.38	1.36	1.37
	AGU	0.61	0.61	0.64	0.57	0.92	0.87	0.93	0.92
	**UCA**	1.3	1.41	1.35	1.19	1.16	1.15	1.05	1.2
	UCC	1.17	1.09	1.17	1.19	1.13	1.15	1.17	1.1
	*UCG*	0.48	0.42	0.53	0.42	0.39	0.43	0.38	0.34
	UCU	1.87	1.89	1.76	2.05	1.03	1.03	1.11	1.06

TER	**UAA**	1.7	0.9	1.96	1.52	0.69	0.71	0.72	0.69
	UAG	0.57	0.7	0.12	1.46	0.54	0.47	0.43	0.54
	UGA	0.73	1.4	0.92	0.02	1.77	1.82	1.85	1.77

THR	ACA	1.22	1.26	1.2	1.23	1.33	1.3	1.26	1.38
	**ACC**	1.28	1.34	1.3	1.24	1.19	1.18	1.25	1.18
	*ACG*	0.37	0.24	0.39	0.34	0.52	0.56	0.47	0.44
	ACU	1.14	1.17	1.11	1.18	0.97	0.95	1.02	1

TYR	*UAC*	0.89	0.95	0.87	0.92	1.03	1.05	1.02	1.02
	**UAU**	1.11	1.05	1.13	1.08	0.97	0.95	0.98	0.98

VAL	*GUA*	0.63	0.73	0.65	0.58	0.62	0.59	0.57	0.62
	GUC	1.15	1.18	1.16	1.12	0.95	1.06	1.01	0.93
	**GUG**	1.19	1.17	1.16	1.24	1.55	1.46	1.52	1.55
	GUU	1.03	0.92	1.03	1.06	0.88	0.89	0.9	0.9

*Note*: AA means, amino acids; green marked the value of RSCU <0.6 (underrepresented); orange marked the value of RSCU >1; red marked the value of RSCU >1.6 (overrepresented); the most preferred codons and dispreferred codons used in coding each amino acid are marked in bold and italic, respectively. Most preferred or dispreferred codons in both of virus and host are underline.

**Table 2:** The most preferred codon of RABV for each amino acid and isoacceptor tRNAs in its hosts.

AA	Most preferred codons in RABV	tRNA Isotypes in hosts			
		*Myotis lucifugus*	*Mustela putorius* furo	Canis familiaris	*Homo sapiens*
Ala	GCA	AGC (8/12)	AGC (15/28)	AGC (13/27)	AGC (26/38)
Arg	AGA	ACG (6/23)	CCT (7/29)	CCT (7/25)	ACG (7/28)
Asn	AAC	**GTT** (9/9)	**GTT** (11/11)	**GTT** (14/14)	**GTT** (25/25)
Asp	GAC	GCT (7/7)	**GTC** (7/7)	**GTC** (11/11)	**GTC** (13/13)
Cys	UGU	**ACA** (27/27)	GCA (25/25)	GCA (19/19)	GCA (29/29)
Gln	CAG	**CTG** (6/9)	**CTG** (7/11)	**CTG** (10/14)	**CTG** (13/19)
Glu	GAG	**CTC** (6/11)	TTC (7/13)	TTC (9/16)	**CTC**, TTC (8/16)
Gly	GGG	GCC (8/17)	GCC (9/15)	GCC (11/26)	GCC (14/28)
His	CAU	GTG (6/6)	GTG (6/6)	GTG (8/8)	GTG (9/9)
Ile	AUC	AAT (7/9)	AAT (10/15)	AAT (8/13)	AAT (15/23)
Leu	UUG	CAG (5/15)	AAG, **CAA** (6/24)	CAG (6/21)	AAG, CAG (9/31)
Lys	AAG	**CTT** (12/15)	**CTT** (37/48)	**CTT** (38/48)	**CTT** (15/27)
Phe	UUC	**GAA** (5/5)	**GAA** (11/11)	**GAA** (10/10)	**GAA** (10/10)
Pro	CCU	**AGG** (6/13)	**AGG** (9/16)	**AGG** (8/17)	**AGG** (9/20)
Ser	UCU	**AGA** (6/15)	GCT (10/25)	**AGA** (10/25)	**AGA** (9/25)
Thr	ACC	AGT, TGT (5/13)	AGT (10/20)	AGT (8/15)	AGT (9/20)
Tyr	UAU	**GTA** (5/5)	**GTA** (9/9)	**GTA** (8/8)	**GTA** (13/13)
Val	GUG	AAC (5/11)	AAC (8/21)	**CAC** (8/19)	**CAC** (13/27)

*Note*: most abundant isoacceptor tRNAs in its hosts matching the most preferred codons of RABV are marked in bold.

## Data Availability

The data used to support this research article (supplementary materials, sequence alignments, and phylogenetic trees) are available on request at https://doi.org/10.6084/m9.figshare.24077211.

## Supplementary Materials

Supplementary 1 Table S1: sequences data. Table S2: the CAI, RADI, and SiD values of the selective sequences.

Supplementary 2 The base composition of the viral coding sequences.
