# Supplementary material for: Analyzing the Evolution and Host Adaptation of the Rabies Virus from the Perspective of Codon Usage Bias
Source: Transbound Emerg Dis. 2023 Oct 10;2023:4667253. doi: 10.1155/2023/4667253 (PMC12016951; doi:10.1155/2023/4667253)
Supplement: Supplementary 1 — Table S1: sequences data. Table S2: the CAI, RADI, and SiD values of the selective sequences. [file 4667253.f1.docx]

**Supplemental Materials**

Table S1 Sequences data

| **Accession** | **Year** | | **Country** | **Host** | **Region** | **Lineage** |
| --- | --- | --- | --- | --- | --- | --- |
| AB569299 | 2008 | Sri Lanka | | Human | Asia | Indian-Sub |
| AB635373 | 2009 | Sri Lanka | | Other | Asia | Indian-Sub |
| AB699220 | 2010 | Bangladesh | | Other | Asia | Arctic |
| AB981663 | 2011 | Laos | | Canis-familiaris | Asia | Asian |
| AB981664 | 2011 | Laos | | Canis-familiaris | Asia | Asian |
| EF564174 | 1956 | China | | Human | Asia | Asian |
| EU293111 | 1983 | Thailand | | Human | Asia | Asian |
| EU293113 | 1990 | Guyana | | Canis-familiaris | South America | Bats |
| EU293115 | 1991 | France | | Wild-Canis | Europe | Cosmopolitan |
| EU293116 | 1997 | Argentina | | Bat | South America | Bats |
| EU311738 | 1999 | Canada | | RAC-SK | North America | RAC-SK |
| EU886631 | 2001 | Germany | | Wild-Canis | Europe | Cosmopolitan |
| EU886632 | 2002 | Germany | | Wild-Canis | Europe | Cosmopolitan |
| EU886633 | 2004 | Austria | | Wild-Canis | Europe | Cosmopolitan |
| EU886634 | 2004 | Germany | | Wild-Canis | Europe | Cosmopolitan |
| EU886635 | 2005 | Germany | | Wild-Canis | Europe | Cosmopolitan |
| EU886636 | 2006 | Austria | | Wild-Canis | Europe | Cosmopolitan |
| FJ712193 | 2008 | China | | Canis-familiaris | Asia | Asian |
| FJ712194 | 2008 | China | | Canis-familiaris | Asia | Asian |
| FJ712195 | 2008 | China | | Other | Asia | Asian |
| FJ712196 | 2008 | China | | Other | Asia | Asian |
| FJ866835 | 2008 | China | | Canis-familiaris | Asia | Asian |
| FJ866836 | 2008 | China | | Canis-familiaris | Asia | Asian |
| FJ959397 | 1956 | China | | Human | Asia | Asian |
| GQ412744 | 1931 | China | | Canis-familiaris | Asia | Cosmopolitan |
| GU345746 | 1992 | China | | Canis-familiaris | Asia | Asian |
| GU345747 | 1986 | China | | Human | Asia | Asian |
| GU345748 | 2006 | China | | Canis-familiaris | Asia | Asian |
| GU358653 | 1994 | China | | Canis-familiaris | Asia | Asian |
| GU647092 | 2008 | China | | Other | Asia | Asian |
| HE802675 | 2010 | Pakistan | | Other | Asia | Arctic |
| HE802676 | 2007 | Pakistan | | Other | Asia | Arctic |
| HQ317918 | 1956 | China | | Human | Asia | Asian |
| HQ450385 | 2008 | China | | Canis-familiaris | Asia | Asian |
| JN234411 | 1931 | China | | Canis-familiaris | Asia | Cosmopolitan |
| JN609295 | 2008 | China | | Canis-familiaris | Asia | Asian |
| JQ423952 | 2011 | China | | Other | Asia | Asian |
| JQ647510 | 2011 | China | | Other | Asia | Asian |
| JQ685892 | 2009 | USA | | Wild-Canis | North America | Bats |
| JQ685893 | 2001 | USA | | RAC-SK | North America | Bats |
| JQ685894 | 1994 | USA | | RAC-SK | North America | Cosmopolitan |
| JQ685895 | 2003 | USA | | Bat | North America | Bats |
| JQ685896 | 2009 | USA | | Wild-Canis | North America | Bats |
| JQ685897 | 2001 | USA | | Bat | North America | Bats |
| JQ685898 | 2009 | USA | | Bat | North America | Bats |
| JQ685899 | 2009 | USA | | Wild-Canis | North America | Cosmopolitan |
| JQ685900 | 2003 | USA | | Bat | North America | Bats |
| JQ685901 | 2003 | USA | | RAC-SK | North America | RAC-SK |
| JQ685902 | 2005 | USA | | Bat | North America | Bats |
| JQ685903 | 2004 | USA | | Bat | North America | Bats |
| JQ685904 | 2001 | USA | | RAC-SK | North America | Bats |
| JQ685905 | 2003 | USA | | Bat | North America | Bats |
| JQ685906 | 2001 | USA | | RAC-SK | North America | Bats |
| JQ685907 | 1996 | USA | | Bat | North America | Bats |
| JQ685908 | 2009 | USA | | Wild-Canis | North America | Bats |
| JQ685909 | 2005 | USA | | Bat | North America | Bats |
| JQ685910 | 2002 | USA | | Bat | North America | Bats |
| JQ685911 | 2001 | USA | | RAC-SK | North America | Bats |
| JQ685912 | 2009 | USA | | Wild-Canis | North America | Bats |
| JQ685913 | 1985 | USA | | Bat | North America | Bats |
| JQ685914 | 2010 | USA | | Wild-Canis | North America | Bats |
| JQ685915 | 2002 | USA | | Bat | North America | Bats |
| JQ685916 | 2002 | USA | | Bat | North America | Bats |
| JQ685917 | 2010 | USA | | Wild-Canis | North America | Bats |
| JQ685918 | 2010 | USA | | Wild-Canis | North America | Bats |
| JQ685919 | 2005 | USA | | Bat | North America | Bats |
| JQ685920 | 1984 | USA | | Bat | North America | Bats |
| JQ685921 | 2001 | USA | | Bat | North America | Bats |
| JQ685922 | 2005 | USA | | Bat | North America | Bats |
| JQ685923 | 1999 | USA | | Bat | North America | Bats |
| JQ685924 | 2010 | USA | | Wild-Canis | North America | Bats |
| JQ685925 | 2004 | USA | | Bat | North America | Bats |
| JQ685926 | 2005 | USA | | Bat | North America | Bats |
| JQ685927 | 2001 | USA | | RAC-SK | North America | Bats |
| JQ685928 | 2009 | USA | | Wild-Canis | North America | Bats |
| JQ685929 | 2009 | Mexico | | RAC-SK | North America | RAC-SK |
| JQ685930 | 2001 | USA | | RAC-SK | North America | Bats |
| JQ685931 | 2000 | USA | | Bat | North America | Bats |
| JQ685932 | 2001 | USA | | RAC-SK | North America | Bats |
| JQ685933 | 2004 | USA | | Wild-Canis | North America | Bats |
| JQ685934 | 2009 | USA | | Wild-Canis | North America | Bats |
| JQ685935 | 2001 | USA | | RAC-SK | North America | Bats |
| JQ685936 | 2009 | Mexico | | Other | North America | Bats |
| JQ685937 | 2009 | USA | | Other | North America | Bats |
| JQ685938 | 2009 | USA | | RAC-SK | North America | RAC-SK |
| JQ685939 | 2009 | USA | | Wild-Canis | North America | Bats |
| JQ685940 | 2001 | USA | | RAC-SK | North America | Bats |
| JQ685941 | 2005 | USA | | RAC-SK | North America | Bats |
| JQ685942 | 1981 | USA | | Bat | North America | Bats |
| JQ685943 | 2009 | USA | | Wild-Canis | North America | Cosmopolitan |
| JQ685944 | 1984 | USA | | RAC-SK | North America | Cosmopolitan |
| JQ685945 | 2005 | USA | | Other | North America | Bats |
| JQ685946 | 1999 | USA | | Bat | North America | Bats |
| JQ685947 | 2004 | USA | | Bat | North America | Bats |
| JQ685948 | 2010 | USA | | Wild-Canis | North America | Bats |
| JQ685949 | 2001 | USA | | RAC-SK | North America | Bats |
| JQ685950 | 2009 | USA | | Bat | North America | Bats |
| JQ685951 | 2010 | USA | | Bat | North America | Bats |
| JQ685952 | 2002 | USA | | Bat | North America | Bats |
| JQ685953 | 2009 | Mexico | | Human | North America | Bats |
| JQ685954 | 2007 | Mexico | | RAC-SK | North America | RAC-SK |
| JQ685955 | 2005 | USA | | Bat | North America | Bats |
| JQ685956 | 1975 | USA | | Bat | North America | Bats |
| JQ685957 | 2009 | USA | | Wild-Canis | North America | Bats |
| JQ685958 | 2001 | USA | | RAC-SK | North America | Bats |
| JQ685959 | 2001 | USA | | RAC-SK | North America | Bats |
| JQ685960 | 2001 | USA | | Bat | North America | Bats |
| JQ685961 | 2010 | USA | | Bat | North America | Bats |
| JQ685962 | 2001 | USA | | RAC-SK | North America | Bats |
| JQ685963 | 2009 | Mexico | | RAC-SK | North America | Bats |
| JQ685964 | 2004 | USA | | RAC-SK | North America | Bats |
| JQ685965 | 2002 | USA | | Bat | North America | Bats |
| JQ685966 | 2001 | USA | | RAC-SK | North America | Bats |
| JQ685968 | 2009 | USA | | RAC-SK | North America | RAC-SK |
| JQ685969 | 2001 | USA | | RAC-SK | North America | Bats |
| JQ685970 | 1974 | USA | | RAC-SK | North America | Cosmopolitan |
| JQ685971 | 2009 | USA | | Bat | North America | Bats |
| JQ685972 | 2009 | USA | | Wild-Canis | North America | Bats |
| JQ685973 | 2011 | USA | | Wild-Canis | North America | Bats |
| JQ685974 | 1995 | USA | | Bat | North America | Bats |
| JQ685975 | 2009 | Mexico | | RAC-SK | North America | Cosmopolitan |
| JQ685976 | 2009 | USA | | Wild-Canis | North America | Bats |
| JQ685977 | 2010 | USA | | Wild-Canis | North America | Bats |
| JQ730682 | 2010 | China | | Canis-familiaris | Asia | Asian |
| JQ944704 | 2009 | Russia | | Canis-familiaris | Europe | Cosmopolitan |
| JQ944705 | 2008 | Russia | | Canis-familiaris | Europe | Cosmopolitan |
| JQ944706 | 2008 | Russia | | Canis-familiaris | Europe | Cosmopolitan |
| JQ944707 | 2008 | Russia | | Other | Europe | Arctic |
| JQ944708 | 2008 | Russia | | Wild-Canis | Europe | Cosmopolitan |
| JQ970480 | 2008 | China | | Canis-familiaris | Asia | Asian |
| JQ970481 | 2006 | China | | Canis-familiaris | Asia | Asian |
| JQ970482 | 2006 | China | | Canis-familiaris | Asia | Asian |
| JQ970483 | 2008 | China | | Canis-familiaris | Asia | Asian |
| JQ970484 | 2009 | China | | Canis-familiaris | Asia | Asian |
| JQ970485 | 2009 | China | | Canis-familiaris | Asia | Asian |
| JQ970486 | 2007 | China | | Canis-familiaris | Asia | Asian |
| JQ970487 | 2009 | China | | Canis-familiaris | Asia | Asian |
| JX473838 | 2009 | Namibia | | Wild-Canis | Africa | Cosmopolitan |
| JX473839 | 2009 | Namibia | | Wild-Canis | Africa | Cosmopolitan |
| JX473840 | 2009 | Namibia | | Other | Africa | Cosmopolitan |
| JX473841 | 2009 | Namibia | | Other | Africa | Cosmopolitan |
| KC169986 | 2009 | China | | Other | Asia | Asian |
| KC171643 | 2008 | South Korea | | Other | Asia | Arctic |
| KC171644 | 2004 | South Korea | | Canis-familiaris | Asia | Arctic |
| KC171645 | 1999 | South Korea | | Canis-familiaris | Asia | Arctic |
| KC193267 | 2011 | China | | Other | Asia | Asian |
| KC196743 | 2011 | Nigeria | | Canis-familiaris | Africa | Africa-2 |
| KC252633 | 2011 | China | | Other | Asia | Cosmopolitan |
| KC252634 | 2011 | China | | Canis-familiaris | Asia | Asian |
| KC595280 | 2011 | Russia | | Wild-Canis | Europe | Cosmopolitan |
| KC595281 | 2011 | Russia | | Other | Europe | Cosmopolitan |
| KC595282 | 2011 | Russia | | Wild-Canis | Europe | Cosmopolitan |
| KC595283 | 2011 | Russia | | Wild-Canis | Europe | Cosmopolitan |
| KC660078 | 2012 | China | | Wild-Canis | Asia | Asian |
| KC737850 | 2011 | USA | | Human | North America | Cosmopolitan |
| KC762941 | 2009 | China | | Other | Asia | Asian |
| KC977995 | 2009 | China | | Canis-familiaris | Asia | Asian |
| KF154996 | 1987 | UK | | Human | Europe | Arctic |
| KF154998 | 1950 | Israel | | Canis-familiaris | Asia | Cosmopolitan |
| KF154999 | 2008 | UK | | Canis-familiaris | Europe | Indian-Sub |
| KF155000 | 2010 | Iraq | | Other | Asia | Cosmopolitan |
| KF155001 | 2009 | Morocco | | Other | Africa | Cosmopolitan |
| KF155002 | 2010 | Tanzania | | Canis-familiaris | Africa | Cosmopolitan |
| KF620487 | 2012 | China | | Other | Asia | Asian |
| KF620488 | 2012 | China | | Other | Asia | Asian |
| KF620489 | 2013 | China | | Other | Asia | Asian |
| KF726852 | 2009 | China | | Other | Asia | Asian |
| KF726853 | 2010 | China | | Other | Asia | Asian |
| KF977826 | 2011 | CAR | | Human | Africa | Africa-2 |
| KJ004416 | 2013 | China | | Human | Asia | Asian |
| KJ564280 | 2013 | China | | Other | Asia | Asian |
| KM016899 | 2014 | China | | Wild-Canis | Asia | Cosmopolitan |
| KM272192 | 2012 | China | | Canis-familiaris | Asia | Arctic |
| KM594023 | 2008 | Brazil | | Other | South America | Bats |
| KM594024 | 2012 | Brazil | | Other | South America | Bats |
| KM594025 | 2007 | Brazil | | Other | South America | Bats |
| KM594029 | 2006 | Brazil | | Bat | South America | Bats |
| KM594030 | 2010 | Brazil | | Bat | South America | Bats |
| KM594031 | 2010 | Brazil | | Bat | South America | Bats |
| KM594032 | 2010 | Brazil | | Bat | South America | Bats |
| KM594033 | 2010 | Brazil | | Bat | South America | Bats |
| KM594034 | 2010 | Brazil | | Other | South America | Bats |
| KM594035 | 2010 | Brazil | | Other | South America | Bats |
| KM594036 | 2010 | Brazil | | Other | South America | Bats |
| KM594037 | 2009 | Brazil | | Bat | South America | Bats |
| KM594038 | 2010 | Brazil | | Bat | South America | Bats |
| KM594039 | 2009 | Brazil | | Wild-Canis | South America | Cosmopolitan |
| KM594040 | 2013 | Brazil | | Bat | South America | Bats |
| KM594041 | 2013 | Brazil | | Bat | South America | Bats |
| KM594042 | 2013 | Brazil | | Bat | South America | Bats |
| KM594043 | 2012 | Brazil | | Other | South America | Bats |
| KP723638 | 2014 | Ethiopia | | Wild-Canis | Africa | Cosmopolitan |
| KP997032 | 2014 | Russia | | Other | Europe | Cosmopolitan |
| KR230089 | 2015 | China | | Other | Asia | Asian |
| KR230090 | 2015 | China | | Other | Asia | Asian |
| KR534217 | 2008 | Tanzania | | Canis-familiaris | Africa | Cosmopolitan |
| KR534218 | 2008 | Tanzania | | Canis-familiaris | Africa | Cosmopolitan |
| KR534219 | 2007 | Tanzania | | Canis-familiaris | Africa | Cosmopolitan |
| KR534220 | 2008 | Tanzania | | Canis-familiaris | Africa | Cosmopolitan |
| KR534228 | 2010 | Tanzania | | Canis-familiaris | Africa | Cosmopolitan |
| KR534229 | 2010 | Tanzania | | Canis-familiaris | Africa | Cosmopolitan |
| KR534230 | 2010 | Tanzania | | Canis-familiaris | Africa | Cosmopolitan |
| KR534231 | 2010 | Tanzania | | Canis-familiaris | Africa | Cosmopolitan |
| KR534232 | 2011 | Tanzania | | Wild-Canis | Africa | Cosmopolitan |
| KR534233 | 2011 | Tanzania | | Canis-familiaris | Africa | Cosmopolitan |
| KR534234 | 2011 | Tanzania | | Other | Africa | Cosmopolitan |
| KR534235 | 2011 | Tanzania | | Other | Africa | Cosmopolitan |
| KR534236 | 2011 | Tanzania | | Other | Africa | Cosmopolitan |
| KR534237 | 2011 | Tanzania | | Other | Africa | Cosmopolitan |
| KR534238 | 2011 | Tanzania | | Canis-familiaris | Africa | Cosmopolitan |
| KR534244 | 2011 | Tanzania | | Canis-familiaris | Africa | Cosmopolitan |
| KR534245 | 2011 | Tanzania | | Other | Africa | Cosmopolitan |
| KR534246 | 2011 | Tanzania | | Other | Africa | Cosmopolitan |
| KR534247 | 2011 | Tanzania | | Canis-familiaris | Africa | Cosmopolitan |
| KR534248 | 2011 | Tanzania | | Other | Africa | Cosmopolitan |
| KR534249 | 2011 | Tanzania | | Canis-familiaris | Africa | Cosmopolitan |
| KR534250 | 2011 | Tanzania | | Canis-familiaris | Africa | Cosmopolitan |
| KR534251 | 2011 | Tanzania | | Canis-familiaris | Africa | Cosmopolitan |
| KR534252 | 2011 | Tanzania | | Canis-familiaris | Africa | Cosmopolitan |
| KR534253 | 2011 | Tanzania | | Canis-familiaris | Africa | Cosmopolitan |
| KR534254 | 2011 | Tanzania | | Other | Africa | Cosmopolitan |
| KR534256 | 2011 | Tanzania | | Other | Africa | Cosmopolitan |
| KR906734 | 2008 | Tanzania | | Canis-familiaris | Africa | Cosmopolitan |
| KR906735 | 2008 | Tanzania | | Canis-familiaris | Africa | Cosmopolitan |
| KR906736 | 2008 | Tanzania | | Canis-familiaris | Africa | Cosmopolitan |
| KR906737 | 2008 | Tanzania | | Canis-familiaris | Africa | Cosmopolitan |
| KR906738 | 2004 | Tanzania | | Other | Africa | Cosmopolitan |
| KR906739 | 2004 | Tanzania | | Canis-familiaris | Africa | Cosmopolitan |
| KR906740 | 2009 | Tanzania | | Other | Africa | Cosmopolitan |
| KR906741 | 2003 | Tanzania | | Canis-familiaris | Africa | Cosmopolitan |
| KR906742 | 2009 | Tanzania | | Canis-familiaris | Africa | Cosmopolitan |
| KR906743 | 2010 | Tanzania | | Canis-familiaris | Africa | Cosmopolitan |
| KR906744 | 2010 | Tanzania | | Canis-familiaris | Africa | Cosmopolitan |
| KR906745 | 2010 | Tanzania | | Canis-familiaris | Africa | Cosmopolitan |
| KR906746 | 2010 | Tanzania | | Canis-familiaris | Africa | Cosmopolitan |
| KR906747 | 2010 | Tanzania | | Canis-familiaris | Africa | Cosmopolitan |
| KR906748 | 2010 | Tanzania | | Canis-familiaris | Africa | Cosmopolitan |
| KR906749 | 2010 | Tanzania | | Canis-familiaris | Africa | Cosmopolitan |
| KR906750 | 2010 | Tanzania | | Canis-familiaris | Africa | Cosmopolitan |
| KR906751 | 2011 | Tanzania | | Canis-familiaris | Africa | Cosmopolitan |
| KR906752 | 2011 | Tanzania | | Canis-familiaris | Africa | Cosmopolitan |
| KR906753 | 2011 | Tanzania | | Other | Africa | Cosmopolitan |
| KR906754 | 2011 | Tanzania | | Canis-familiaris | Africa | Cosmopolitan |
| KR906755 | 2011 | Tanzania | | Canis-familiaris | Africa | Cosmopolitan |
| KR906756 | 2011 | Tanzania | | Canis-familiaris | Africa | Cosmopolitan |
| KR906757 | 2010 | Tanzania | | Canis-familiaris | Africa | Cosmopolitan |
| KR906759 | 2010 | Tanzania | | Canis-familiaris | Africa | Cosmopolitan |
| KR906760 | 2010 | Tanzania | | Canis-familiaris | Africa | Cosmopolitan |
| KR906762 | 2010 | Tanzania | | Canis-familiaris | Africa | Cosmopolitan |
| KR906763 | 2010 | Tanzania | | Canis-familiaris | Africa | Cosmopolitan |
| KR906764 | 2010 | Tanzania | | Other | Africa | Cosmopolitan |
| KR906765 | 2010 | Tanzania | | Canis-familiaris | Africa | Cosmopolitan |
| KR906766 | 2010 | Tanzania | | Canis-familiaris | Africa | Cosmopolitan |
| KR906767 | 2011 | Tanzania | | Canis-familiaris | Africa | Cosmopolitan |
| KR906768 | 2011 | Tanzania | | Canis-familiaris | Africa | Cosmopolitan |
| KR906769 | 2011 | Tanzania | | Canis-familiaris | Africa | Cosmopolitan |
| KR906770 | 2011 | Tanzania | | Canis-familiaris | Africa | Cosmopolitan |
| KR906771 | 2011 | Tanzania | | Canis-familiaris | Africa | Cosmopolitan |
| KR906772 | 2011 | Tanzania | | Canis-familiaris | Africa | Cosmopolitan |
| KR906773 | 2011 | Tanzania | | Canis-familiaris | Africa | Cosmopolitan |
| KR906774 | 2011 | Tanzania | | Canis-familiaris | Africa | Cosmopolitan |
| KR906775 | 2011 | Tanzania | | Other | Africa | Cosmopolitan |
| KR906776 | 2011 | Tanzania | | Canis-familiaris | Africa | Cosmopolitan |
| KR906777 | 2011 | Tanzania | | Other | Africa | Cosmopolitan |
| KR906778 | 2011 | Tanzania | | Other | Africa | Cosmopolitan |
| KR906779 | 2012 | Tanzania | | Other | Africa | Cosmopolitan |
| KR906780 | 2011 | Tanzania | | Canis-familiaris | Africa | Cosmopolitan |
| KR906781 | 2012 | Tanzania | | Other | Africa | Cosmopolitan |
| KR906782 | 2012 | Tanzania | | Canis-familiaris | Africa | Cosmopolitan |
| KR906783 | 2012 | Tanzania | | Canis-familiaris | Africa | Cosmopolitan |
| KR906784 | 2012 | Tanzania | | Canis-familiaris | Africa | Cosmopolitan |
| KR906785 | 2012 | Tanzania | | Other | Africa | Cosmopolitan |
| KR906786 | 2012 | Tanzania | | Other | Africa | Cosmopolitan |
| KR906787 | 2012 | Tanzania | | Other | Africa | Cosmopolitan |
| KR906788 | 2012 | Tanzania | | Other | Africa | Cosmopolitan |
| KR906789 | 2012 | Tanzania | | Canis-familiaris | Africa | Cosmopolitan |
| KR906790 | 2012 | Tanzania | | Canis-familiaris | Africa | Cosmopolitan |
| KR906791 | 2012 | Tanzania | | Canis-familiaris | Africa | Cosmopolitan |
| KR906792 | 2012 | Tanzania | | Canis-familiaris | Africa | Cosmopolitan |
| KT006769 | 2013 | Mexico | | Canis-familiaris | North America | Cosmopolitan |
| KT336432 | 2012 | South Africa | | Canis-familiaris | Africa | Cosmopolitan |
| KT336433 | 1991 | Zimbabwe | | Canis-familiaris | Africa | Cosmopolitan |
| KT336434 | 1992 | Zimbabwe | | Canis-familiaris | Africa | Cosmopolitan |
| KT336435 | 1993 | Zimbabwe | | Canis-familiaris | Africa | Cosmopolitan |
| KT336436 | 2012 | South Africa | | Canis-familiaris | Africa | Cosmopolitan |
| KT336437 | 2012 | South Africa | | Canis-familiaris | Africa | Cosmopolitan |
| KT728348 | 2003 | Russia | | Human | Europe | Cosmopolitan |
| KT728349 | 2003 | Russia | | Human | Europe | Cosmopolitan |
| KU198460 | 1989 | USA | | Wild-Canis | North America | Arctic |
| KU198461 | 1990 | Canada | | Wild-Canis | North America | Arctic |
| KU198462 | 1990 | USA | | Wild-Canis | North America | Arctic |
| KU198463 | 1990 | USA | | Wild-Canis | North America | Arctic |
| KU198464 | 1991 | Canada | | Wild-Canis | North America | Arctic |
| KU198465 | 1992 | Canada | | Wild-Canis | North America | Arctic |
| KU198466 | 1993 | Canada | | Wild-Canis | North America | Arctic |
| KU198467 | 1997 | Canada | | Wild-Canis | North America | Arctic |
| KU198468 | 2000 | Canada | | Wild-Canis | North America | Arctic |
| KU198469 | 2001 | Canada | | Wild-Canis | North America | Arctic |
| KU198470 | 2002 | Canada | | Wild-Canis | North America | Arctic |
| KU198471 | 2007 | USA | | Wild-Canis | North America | Arctic |
| KU198472 | 2012 | Canada | | Wild-Canis | North America | Arctic |
| KU198473 | 2012 | Canada | | Wild-Canis | North America | Arctic |
| KU198474 | 2012 | Canada | | Wild-Canis | North America | Arctic |
| KU198475 | 2013 | Canada | | Wild-Canis | North America | Arctic |
| KU198476 | 2013 | Canada | | Wild-Canis | North America | Arctic |
| KU198477 | 2013 | Canada | | Wild-Canis | North America | Arctic |
| KU198478 | 2013 | Canada | | Wild-Canis | North America | Arctic |
| KU198479 | 2014 | Canada | | Wild-Canis | North America | Arctic |
| KU523255 | 2010 | French Guiana | | Bat | South America | Bats |
| KX036361 | 1990 | Greenland | | Wild-Canis | North America | Arctic |
| KX036362 | 2001 | Greenland | | Wild-Canis | North America | Arctic |
| KX036363 | 2002 | Greenland | | Wild-Canis | North America | Arctic |
| KX036364 | 2002 | Greenland | | Wild-Canis | North America | Arctic |
| KX036365 | 2002 | Greenland | | Wild-Canis | North America | Arctic |
| KX036366 | 2002 | Greenland | | Wild-Canis | North America | Arctic |
| KX036367 | 2002 | Greenland | | Wild-Canis | North America | Arctic |
| KX148100 | 2009 | French Guiana | | Bat | South America | Bats |
| KX148101 | 1979 | Egypt | | Human | Africa | Cosmopolitan |
| KX148102 | 1991 | Mexico | | Canis-familiaris | North America | Cosmopolitan |
| KX148103 | 1981 | South Africa | | Human | Africa | Cosmopolitan |
| KX148104 | 1978 | Montenegro | | Other | Europe | Cosmopolitan |
| KX148105 | 1980 | Greenland | | Canis-familiaris | North America | Arctic |
| KX148106 | 1990 | USA | | Wild-Canis | North America | Arctic |
| KX148107 | 1986 | Benin | | Other | Africa | Africa-2 |
| KX148108 | 2011 | Nepal | | Other | Asia | Indian-Sub |
| KX148109 | 1986 | Brazil | | Canis-familiaris | South America | Bats |
| KX148110 | 1991 | Mexico | | Canis-familiaris | North America | Cosmopolitan |
| KX148111 | 1991 | Mexico | | Canis-familiaris | North America | Cosmopolitan |
| KX148112 | 1991 | Mexico | | Human | North America | Cosmopolitan |
| KX148113 | 1991 | Germany | | Wild-Canis | Europe | Cosmopolitan |
| KX148114 | 1995 | Poland | | Wild-Canis | Europe | Cosmopolitan |
| KX148115 | 1996 | Poland | | Wild-Canis | Europe | Cosmopolitan |
| KX148116 | 1996 | Poland | | Wild-Canis | Europe | Cosmopolitan |
| KX148117 | 1997 | Poland | | Wild-Canis | Europe | Cosmopolitan |
| KX148118 | 1994 | Poland | | Canis-familiaris | Europe | Cosmopolitan |
| KX148119 | 1993 | Poland | | Wild-Canis | Europe | Cosmopolitan |
| KX148120 | 1993 | Poland | | Canis-familiaris | Europe | Cosmopolitan |
| KX148121 | 1993 | Poland | | Wild-Canis | Europe | Cosmopolitan |
| KX148122 | 1991 | Germany | | Wild-Canis | Europe | Cosmopolitan |
| KX148123 | 1991 | Germany | | Wild-Canis | Europe | Cosmopolitan |
| KX148124 | 1991 | Germany | | Wild-Canis | Europe | Cosmopolitan |
| KX148125 | 1994 | Belgium | | Wild-Canis | Europe | Cosmopolitan |
| KX148126 | 1996 | France | | Wild-Canis | Europe | Cosmopolitan |
| KX148127 | 1991 | France | | Wild-Canis | Europe | Cosmopolitan |
| KX148128 | 1992 | France | | Wild-Canis | Europe | Cosmopolitan |
| KX148129 | 1994 | Slovenia | | Wild-Canis | Europe | Cosmopolitan |
| KX148130 | 1994 | Slovenia | | Wild-Canis | Europe | Cosmopolitan |
| KX148131 | 1994 | Slovenia | | Wild-Canis | Europe | Cosmopolitan |
| KX148132 | 1994 | Slovenia | | Wild-Canis | Europe | Cosmopolitan |
| KX148133 | 1986 | Bosnia | | Wild-Canis | Europe | Cosmopolitan |
| KX148134 | 1994 | France | | Wild-Canis | Europe | Cosmopolitan |
| KX148135 | 1991 | Germany | | Wild-Canis | Europe | Cosmopolitan |
| KX148136 | 1993 | Hungary | | Wild-Canis | Europe | Cosmopolitan |
| KX148137 | 1993 | Hungary | | Wild-Canis | Europe | Cosmopolitan |
| KX148138 | 1993 | Hungary | | Wild-Canis | Europe | Cosmopolitan |
| KX148139 | 1993 | Hungary | | Wild-Canis | Europe | Cosmopolitan |
| KX148140 | 1986 | Serbia | | Wild-Canis | Europe | Cosmopolitan |
| KX148141 | 1992 | Poland | | Wild-Canis | Europe | Cosmopolitan |
| KX148142 | 1994 | Poland | | Wild-Canis | Europe | Cosmopolitan |
| KX148143 | 1993 | Hungary | | Wild-Canis | Europe | Cosmopolitan |
| KX148144 | 1994 | Slovenia | | Wild-Canis | Europe | Cosmopolitan |
| KX148145 | 1986 | Bosnia | | Wild-Canis | Europe | Cosmopolitan |
| KX148146 | 1993 | Finland | | Wild-Canis | Europe | Cosmopolitan |
| KX148147 | 1988 | Finland | | Canis-familiaris | Europe | Cosmopolitan |
| KX148148 | 1992 | Estonia | | Wild-Canis | Europe | Cosmopolitan |
| KX148149 | 1991 | Estonia | | Canis-familiaris | Europe | Cosmopolitan |
| KX148150 | 1986 | Poland | | Canis-familiaris | Europe | Cosmopolitan |
| KX148151 | 1996 | Poland | | Wild-Canis | Europe | Cosmopolitan |
| KX148152 | 1996 | Poland | | Canis-familiaris | Europe | Cosmopolitan |
| KX148153 | 1996 | Poland | | Canis-familiaris | Europe | Cosmopolitan |
| KX148154 | 1997 | Poland | | Canis-familiaris | Europe | Cosmopolitan |
| KX148155 | 1993 | Estonia | | Wild-Canis | Europe | Cosmopolitan |
| KX148156 | 1991 | Estonia | | Canis-familiaris | Europe | Cosmopolitan |
| KX148157 | 1993 | Estonia | | Canis-familiaris | Europe | Cosmopolitan |
| KX148158 | 1993 | Estonia | | Wild-Canis | Europe | Cosmopolitan |
| KX148159 | 1974 | Iran | | Other | Asia | Cosmopolitan |
| KX148160 | 1991 | Hungary | | Human | Europe | Cosmopolitan |
| KX148161 | 1972 | Serbia | | Wild-Canis | Europe | Cosmopolitan |
| KX148162 | 1993 | Turkey | | Canis-familiaris | Europe | Cosmopolitan |
| KX148163 | 1993 | Turkey | | Canis-familiaris | Europe | Cosmopolitan |
| KX148164 | 1993 | Turkey | | Canis-familiaris | Europe | Cosmopolitan |
| KX148165 | 1993 | Turkey | | Canis-familiaris | Europe | Cosmopolitan |
| KX148166 | 1993 | Turkey | | Canis-familiaris | Europe | Cosmopolitan |
| KX148167 | 1993 | Turkey | | Canis-familiaris | Europe | Cosmopolitan |
| KX148168 | 1987 | Saudi Arabia | | Wild-Canis | Asia | Cosmopolitan |
| KX148169 | 1990 | Oman | | Other | Asia | Cosmopolitan |
| KX148170 | 1990 | Oman | | Wild-Canis | Asia | Cosmopolitan |
| KX148171 | 1991 | USA | | Wild-Canis | North America | Cosmopolitan |
| KX148172 | 1987 | Saudi Arabia | | Other | Asia | Cosmopolitan |
| KX148173 | 1997 | Saudi Arabia | | Wild-Canis | Asia | Cosmopolitan |
| KX148174 | 1998 | Saudi Arabia | | Wild-Canis | Asia | Cosmopolitan |
| KX148175 | 1998 | Saudi Arabia | | Wild-Canis | Asia | Cosmopolitan |
| KX148176 | 2004 | Saudi Arabia | | Wild-Canis | Asia | Cosmopolitan |
| KX148177 | 1996 | Israel | | Wild-Canis | Asia | Cosmopolitan |
| KX148178 | 1996 | Israel | | Canis-familiaris | Asia | Cosmopolitan |
| KX148179 | 1996 | Israel | | Wild-Canis | Asia | Cosmopolitan |
| KX148180 | 1993 | Israel | | Wild-Canis | Asia | Cosmopolitan |
| KX148181 | 1996 | Israel | | Canis-familiaris | Asia | Cosmopolitan |
| KX148182 | 1996 | Israel | | Canis-familiaris | Asia | Cosmopolitan |
| KX148183 | 1993 | Israel | | Wild-Canis | Asia | Cosmopolitan |
| KX148184 | 1996 | Israel | | Other | Asia | Cosmopolitan |
| KX148185 | 1996 | Iran | | Wild-Canis | Asia | Cosmopolitan |
| KX148186 | 1984 | Iran | | Wild-Canis | Asia | Cosmopolitan |
| KX148187 | 1996 | Iran | | Wild-Canis | Asia | Cosmopolitan |
| KX148188 | 1991 | Iran | | Wild-Canis | Asia | Cosmopolitan |
| KX148189 | 1985 | Iran | | Canis-familiaris | Asia | Cosmopolitan |
| KX148190 | 1976 | Iran | | Wild-Canis | Asia | Cosmopolitan |
| KX148191 | 1993 | Israel | | Wild-Canis | Asia | Cosmopolitan |
| KX148192 | 1993 | Israel | | Canis-familiaris | Asia | Cosmopolitan |
| KX148193 | 2008 | Morocco | | Canis-familiaris | Africa | Cosmopolitan |
| KX148194 | 1989 | Morocco | | Canis-familiaris | Africa | Cosmopolitan |
| KX148195 | 2004 | Morocco | | Canis-familiaris | Africa | Cosmopolitan |
| KX148196 | 1990 | Morocco | | Canis-familiaris | Africa | Cosmopolitan |
| KX148197 | 2015 | Algeria | | Canis-familiaris | Africa | Cosmopolitan |
| KX148198 | 1993 | Somalia | | Canis-familiaris | Africa | Cosmopolitan |
| KX148199 | 1993 | Somalia | | Wild-Canis | Africa | Cosmopolitan |
| KX148200 | 1988 | Ethiopia | | Canis-familiaris | Africa | Cosmopolitan |
| KX148201 | 1986 | Nigeria | | Canis-familiaris | Africa | Cosmopolitan |
| KX148202 | 1995 | Gabon | | Canis-familiaris | Africa | Cosmopolitan |
| KX148203 | 1986 | Mozambique | | Canis-familiaris | Africa | Cosmopolitan |
| KX148204 | 1992 | Namibia | | Canis-familiaris | Africa | Cosmopolitan |
| KX148205 | 1994 | Rwanda | | Canis-familiaris | Africa | Cosmopolitan |
| KX148206 | 1996 | Tanzania | | Canis-familiaris | Africa | Cosmopolitan |
| KX148207 | 2014 | Kenya | | Human | Africa | Cosmopolitan |
| KX148208 | 1992 | CAR | | Canis-familiaris | Africa | Cosmopolitan |
| KX148209 | 2004 | Madagascar | | Canis-familiaris | Africa | Cosmopolitan |
| KX148210 | 1998 | Madagascar | | Human | Africa | Cosmopolitan |
| KX148211 | 1986 | Madagascar | | Canis-familiaris | Africa | Cosmopolitan |
| KX148212 | 1993 | Iran | | Wild-Canis | Asia | Cosmopolitan |
| KX148213 | 1982 | USA | | RAC-SK | North America | Cosmopolitan |
| KX148214 | 1995 | Brazil | | Canis-familiaris | South America | Cosmopolitan |
| KX148215 | 1995 | Brazil | | Canis-familiaris | South America | Cosmopolitan |
| KX148216 | 1986 | Brazil | | Canis-familiaris | South America | Cosmopolitan |
| KX148217 | 1986 | Brazil | | Canis-familiaris | South America | Cosmopolitan |
| KX148218 | 2009 | Botswana | | Other | Africa | Africa-3 |
| KX148219 | 2009 | Botswana | | Other | Africa | Africa-3 |
| KX148220 | 2013 | South Africa | | Other | Africa | Africa-3 |
| KX148221 | 2014 | South Africa | | Other | Africa | Africa-3 |
| KX148222 | 2014 | South Africa | | Other | Africa | Africa-3 |
| KX148223 | 2000 | South Africa | | Other | Africa | Africa-3 |
| KX148224 | 1991 | USA | | RAC-SK | North America | Arctic |
| KX148225 | 2002 | Afghanistan | | Canis-familiaris | Asia | Arctic |
| KX148226 | 2002 | Afghanistan | | Canis-familiaris | Asia | Arctic |
| KX148227 | 2004 | Afghanistan | | Canis-familiaris | Asia | Arctic |
| KX148228 | 1998 | Nepal | | Canis-familiaris | Asia | Arctic |
| KX148229 | 1990 | Niger | | Canis-familiaris | Africa | Africa-2 |
| KX148230 | 1995 | Burkina | | Canis-familiaris | Africa | Africa-2 |
| KX148231 | 1990 | Niger | | Canis-familiaris | Africa | Africa-2 |
| KX148232 | 1992 | Côte | | Canis-familiaris | Africa | Africa-2 |
| KX148233 | 1992 | Côte | | Canis-familiaris | Africa | Africa-2 |
| KX148234 | 1986 | Burkina | | Canis-familiaris | Africa | Africa-2 |
| KX148235 | 2001 | Côte | | Canis-familiaris | Africa | Africa-2 |
| KX148236 | 1993 | Mauritania | | Canis-familiaris | Africa | Africa-2 |
| KX148237 | 1993 | Mauritania | | Canis-familiaris | Africa | Africa-2 |
| KX148238 | 1991 | Senegal | | Canis-familiaris | Africa | Africa-2 |
| KX148239 | 1992 | Senegal | | Canis-familiaris | Africa | Africa-2 |
| KX148240 | 1990 | Chad | | Canis-familiaris | Africa | Africa-2 |
| KX148241 | 1996 | Chad | | Canis-familiaris | Africa | Africa-2 |
| KX148242 | 1994 | Cameroon | | Canis-familiaris | Africa | Africa-2 |
| KX148243 | 1987 | Cameroon | | Canis-familiaris | Africa | Africa-2 |
| KX148244 | 1990 | Guinea | | Canis-familiaris | Africa | Africa-2 |
| KX148245 | 2009 | Nepal | | Other | Asia | Indian-Sub |
| KX148246 | 1997 | India | | Human | Asia | Indian-Sub |
| KX148247 | 1999 | Myanmar | | Canis-familiaris | Asia | Asian |
| KX148248 | 1999 | Myanmar | | Canis-familiaris | Asia | Asian |
| KX148249 | 1998 | Cambodia | | Canis-familiaris | Asia | Asian |
| KX148250 | 1999 | Cambodia | | Canis-familiaris | Asia | Asian |
| KX148251 | 1998 | Cambodia | | Canis-familiaris | Asia | Asian |
| KX148252 | 1999 | Cambodia | | Canis-familiaris | Asia | Asian |
| KX148253 | 1998 | Cambodia | | Canis-familiaris | Asia | Asian |
| KX148254 | 2001 | Vietnam | | Canis-familiaris | Asia | Asian |
| KX148255 | 1999 | Laos | | Canis-familiaris | Asia | Asian |
| KX148256 | 2002 | Laos | | Canis-familiaris | Asia | Asian |
| KX148257 | 2002 | Laos | | Canis-familiaris | Asia | Asian |
| KX148258 | 2002 | Laos | | Canis-familiaris | Asia | Asian |
| KX148259 | 1994 | Philippines | | Canis-familiaris | Asia | Asian |
| KX148260 | 2004 | Philippines | | Canis-familiaris | Asia | Asian |
| KX148261 | 1994 | Philippines | | Canis-familiaris | Asia | Asian |
| KX148262 | 1994 | Philippines | | Canis-familiaris | Asia | Asian |
| KX148263 | 1994 | Philippines | | Canis-familiaris | Asia | Asian |
| KX148264 | 1994 | China | | Canis-familiaris | Asia | Asian |
| KX148265 | 1998 | China | | Canis-familiaris | Asia | Asian |
| KX148266 | 2003 | Indonesia | | Canis-familiaris | Asia | Asian |
| KX148267 | 1992 | China | | Human | Asia | Asian |
| KX148268 | 1990 | French Guiana | | Canis-familiaris | South America | Bats |
| KX148269 | 1997 | Argentina | | Bat | South America | Bats |
| KX708499 | 2015 | Mexico | | Canis-familiaris | North America | Cosmopolitan |
| KX708500 | 2014 | Mexico | | Canis-familiaris | North America | Cosmopolitan |
| KX708501 | 2013 | Mexico | | Canis-familiaris | North America | Cosmopolitan |
| KX708502 | 2012 | Mexico | | Canis-familiaris | North America | Cosmopolitan |
| KX708503 | 2014 | Mexico | | Canis-familiaris | North America | Cosmopolitan |
| KX708504 | 2008 | Mexico | | Canis-familiaris | North America | Cosmopolitan |
| KX954123 | 2016 | Russia | | Wild-Canis | Europe | Arctic |
| KY026414 | 2013 | USA | | RAC-SK | North America | RAC-SK |
| KY026415 | 2014 | Canada | | RAC-SK | North America | RAC-SK |
| KY026416 | 1995 | USA | | RAC-SK | North America | RAC-SK |
| KY026417 | 1998 | USA | | RAC-SK | North America | RAC-SK |
| KY026418 | 1998 | USA | | RAC-SK | North America | RAC-SK |
| KY026419 | 2004 | USA | | RAC-SK | North America | RAC-SK |
| KY026420 | 2004 | USA | | RAC-SK | North America | RAC-SK |
| KY026421 | 2011 | USA | | RAC-SK | North America | RAC-SK |
| KY026422 | 1999 | Canada | | RAC-SK | North America | RAC-SK |
| KY026423 | 1999 | Canada | | RAC-SK | North America | RAC-SK |
| KY026424 | 1999 | Canada | | RAC-SK | North America | RAC-SK |
| KY026425 | 1999 | Canada | | RAC-SK | North America | RAC-SK |
| KY026426 | 2000 | Canada | | RAC-SK | North America | RAC-SK |
| KY026427 | 2000 | Canada | | RAC-SK | North America | RAC-SK |
| KY026428 | 2000 | Canada | | RAC-SK | North America | RAC-SK |
| KY026429 | 2000 | Canada | | RAC-SK | North America | RAC-SK |
| KY026430 | 2000 | Canada | | RAC-SK | North America | RAC-SK |
| KY026431 | 2000 | Canada | | RAC-SK | North America | RAC-SK |
| KY026432 | 2000 | Canada | | RAC-SK | North America | RAC-SK |
| KY026433 | 2000 | Canada | | RAC-SK | North America | RAC-SK |
| KY026434 | 2000 | Canada | | RAC-SK | North America | RAC-SK |
| KY026435 | 2000 | Canada | | RAC-SK | North America | RAC-SK |
| KY026436 | 2000 | Canada | | RAC-SK | North America | RAC-SK |
| KY026437 | 2000 | Canada | | RAC-SK | North America | RAC-SK |
| KY026438 | 2000 | Canada | | RAC-SK | North America | RAC-SK |
| KY026439 | 2000 | Canada | | RAC-SK | North America | RAC-SK |
| KY026440 | 2000 | Canada | | RAC-SK | North America | RAC-SK |
| KY026441 | 2000 | Canada | | RAC-SK | North America | RAC-SK |
| KY026442 | 2000 | Canada | | RAC-SK | North America | RAC-SK |
| KY026443 | 2000 | Canada | | RAC-SK | North America | RAC-SK |
| KY026444 | 2001 | Canada | | RAC-SK | North America | RAC-SK |
| KY026445 | 2001 | Canada | | RAC-SK | North America | RAC-SK |
| KY026446 | 2001 | Canada | | RAC-SK | North America | RAC-SK |
| KY026447 | 2001 | Canada | | RAC-SK | North America | RAC-SK |
| KY026448 | 2001 | Canada | | RAC-SK | North America | RAC-SK |
| KY026449 | 2001 | Canada | | RAC-SK | North America | RAC-SK |
| KY026450 | 2001 | Canada | | RAC-SK | North America | RAC-SK |
| KY026451 | 2001 | Canada | | RAC-SK | North America | RAC-SK |
| KY026452 | 2001 | Canada | | RAC-SK | North America | RAC-SK |
| KY026453 | 2001 | Canada | | RAC-SK | North America | RAC-SK |
| KY026454 | 2001 | Canada | | RAC-SK | North America | RAC-SK |
| KY026455 | 2001 | Canada | | RAC-SK | North America | RAC-SK |
| KY026456 | 2001 | Canada | | RAC-SK | North America | RAC-SK |
| KY026457 | 2001 | Canada | | RAC-SK | North America | RAC-SK |
| KY026458 | 2001 | Canada | | RAC-SK | North America | RAC-SK |
| KY026459 | 2002 | Canada | | RAC-SK | North America | RAC-SK |
| KY026460 | 2002 | Canada | | RAC-SK | North America | RAC-SK |
| KY026461 | 2002 | Canada | | RAC-SK | North America | RAC-SK |
| KY026462 | 2002 | Canada | | RAC-SK | North America | RAC-SK |
| KY026463 | 2002 | Canada | | RAC-SK | North America | RAC-SK |
| KY026464 | 2003 | Canada | | RAC-SK | North America | RAC-SK |
| KY026465 | 2003 | Canada | | RAC-SK | North America | RAC-SK |
| KY026466 | 2003 | Canada | | RAC-SK | North America | RAC-SK |
| KY026467 | 2003 | Canada | | RAC-SK | North America | RAC-SK |
| KY026468 | 2003 | Canada | | RAC-SK | North America | RAC-SK |
| KY026469 | 2003 | Canada | | RAC-SK | North America | RAC-SK |
| KY026470 | 2003 | Canada | | RAC-SK | North America | RAC-SK |
| KY026471 | 2003 | Canada | | RAC-SK | North America | RAC-SK |
| KY026472 | 2003 | Canada | | RAC-SK | North America | RAC-SK |
| KY026473 | 2004 | Canada | | RAC-SK | North America | RAC-SK |
| KY026474 | 2004 | Canada | | RAC-SK | North America | RAC-SK |
| KY026475 | 2004 | Canada | | RAC-SK | North America | RAC-SK |
| KY026476 | 2004 | Canada | | RAC-SK | North America | RAC-SK |
| KY026477 | 2005 | Canada | | RAC-SK | North America | RAC-SK |
| KY026478 | 2006 | USA | | RAC-SK | North America | RAC-SK |
| KY026479 | 2006 | USA | | RAC-SK | North America | RAC-SK |
| KY026480 | 2007 | USA | | RAC-SK | North America | RAC-SK |
| KY026481 | 2008 | USA | | RAC-SK | North America | RAC-SK |
| KY026482 | 2008 | USA | | RAC-SK | North America | RAC-SK |
| KY026483 | 2011 | USA | | RAC-SK | North America | RAC-SK |
| KY175229 | 2012 | China | | Canis-familiaris | Asia | Arctic |
| KY175230 | 2015 | China | | Human | Asia | Arctic |
| KY210220 | 2008 | Tanzania | | Other | Africa | Cosmopolitan |
| KY210221 | 2008 | Tanzania | | Other | Africa | Cosmopolitan |
| KY210222 | 2009 | Tanzania | | Canis-familiaris | Africa | Cosmopolitan |
| KY210223 | 2007 | Tanzania | | Canis-familiaris | Africa | Cosmopolitan |
| KY210224 | 2010 | Tanzania | | Canis-familiaris | Africa | Cosmopolitan |
| KY210225 | 2011 | Tanzania | | Other | Africa | Cosmopolitan |
| KY210226 | 2011 | Tanzania | | Canis-familiaris | Africa | Cosmopolitan |
| KY210227 | 2011 | Tanzania | | Canis-familiaris | Africa | Cosmopolitan |
| KY210228 | 2011 | Tanzania | | Other | Africa | Cosmopolitan |
| KY210229 | 2011 | Tanzania | | Canis-familiaris | Africa | Cosmopolitan |
| KY210230 | 2011 | Tanzania | | Other | Africa | Cosmopolitan |
| KY210231 | 2011 | Tanzania | | Canis-familiaris | Africa | Cosmopolitan |
| KY210232 | 2011 | Tanzania | | Canis-familiaris | Africa | Cosmopolitan |
| KY210233 | 2011 | Tanzania | | Other | Africa | Cosmopolitan |
| KY210234 | 2011 | Tanzania | | Canis-familiaris | Africa | Cosmopolitan |
| KY210235 | 2011 | Tanzania | | Canis-familiaris | Africa | Cosmopolitan |
| KY210236 | 2011 | Tanzania | | Other | Africa | Cosmopolitan |
| KY210237 | 2011 | Tanzania | | Other | Africa | Cosmopolitan |
| KY210238 | 2011 | Tanzania | | Canis-familiaris | Africa | Cosmopolitan |
| KY210239 | 2011 | Tanzania | | Canis-familiaris | Africa | Cosmopolitan |
| KY210240 | 2011 | Tanzania | | Other | Africa | Cosmopolitan |
| KY210241 | 2011 | Tanzania | | Other | Africa | Cosmopolitan |
| KY210242 | 2011 | Tanzania | | Other | Africa | Cosmopolitan |
| KY210243 | 2011 | Tanzania | | Canis-familiaris | Africa | Cosmopolitan |
| KY210244 | 2011 | Tanzania | | Canis-familiaris | Africa | Cosmopolitan |
| KY210245 | 2012 | Tanzania | | Canis-familiaris | Africa | Cosmopolitan |
| KY210246 | 2012 | Tanzania | | Other | Africa | Cosmopolitan |
| KY210247 | 2012 | Tanzania | | Canis-familiaris | Africa | Cosmopolitan |
| KY210248 | 2012 | Tanzania | | Canis-familiaris | Africa | Cosmopolitan |
| KY210249 | 2012 | Tanzania | | Canis-familiaris | Africa | Cosmopolitan |
| KY210250 | 2012 | Tanzania | | Canis-familiaris | Africa | Cosmopolitan |
| KY210251 | 2012 | Tanzania | | Canis-familiaris | Africa | Cosmopolitan |
| KY210252 | 2012 | Tanzania | | Canis-familiaris | Africa | Cosmopolitan |
| KY210253 | 2013 | Tanzania | | Canis-familiaris | Africa | Cosmopolitan |
| KY210254 | 2013 | Tanzania | | Canis-familiaris | Africa | Cosmopolitan |
| KY210255 | 2013 | Tanzania | | Canis-familiaris | Africa | Cosmopolitan |
| KY210256 | 2013 | Tanzania | | Canis-familiaris | Africa | Cosmopolitan |
| KY210257 | 2013 | Tanzania | | Canis-familiaris | Africa | Cosmopolitan |
| KY210258 | 2013 | Tanzania | | Canis-familiaris | Africa | Cosmopolitan |
| KY210259 | 2013 | Tanzania | | Other | Africa | Cosmopolitan |
| KY210260 | 2013 | Tanzania | | Canis-familiaris | Africa | Cosmopolitan |
| KY210261 | 2013 | Tanzania | | Other | Africa | Cosmopolitan |
| KY210262 | 2013 | Tanzania | | Other | Africa | Cosmopolitan |
| KY210263 | 2013 | Tanzania | | Canis-familiaris | Africa | Cosmopolitan |
| KY210264 | 2013 | Tanzania | | Canis-familiaris | Africa | Cosmopolitan |
| KY210265 | 2013 | Tanzania | | Canis-familiaris | Africa | Cosmopolitan |
| KY210266 | 2013 | Tanzania | | Canis-familiaris | Africa | Cosmopolitan |
| KY210267 | 2013 | Tanzania | | Canis-familiaris | Africa | Cosmopolitan |
| KY210268 | 2013 | Tanzania | | Other | Africa | Cosmopolitan |
| KY210269 | 2013 | Tanzania | | Canis-familiaris | Africa | Cosmopolitan |
| KY210270 | 2013 | Tanzania | | Canis-familiaris | Africa | Cosmopolitan |
| KY210271 | 2013 | Tanzania | | Canis-familiaris | Africa | Cosmopolitan |
| KY210272 | 2013 | Tanzania | | Canis-familiaris | Africa | Cosmopolitan |
| KY210273 | 2013 | Tanzania | | Canis-familiaris | Africa | Cosmopolitan |
| KY210274 | 2013 | Tanzania | | Other | Africa | Cosmopolitan |
| KY210275 | 2013 | Tanzania | | Other | Africa | Cosmopolitan |
| KY210276 | 2013 | Tanzania | | Other | Africa | Cosmopolitan |
| KY210277 | 2011 | Tanzania | | Other | Africa | Cosmopolitan |
| KY210278 | 2011 | Tanzania | | Canis-familiaris | Africa | Cosmopolitan |
| KY210279 | 2011 | Tanzania | | Canis-familiaris | Africa | Cosmopolitan |
| KY210280 | 2012 | Tanzania | | Canis-familiaris | Africa | Cosmopolitan |
| KY210281 | 2012 | Tanzania | | Canis-familiaris | Africa | Cosmopolitan |
| KY210282 | 2012 | Tanzania | | Canis-familiaris | Africa | Cosmopolitan |
| KY210283 | 2012 | Tanzania | | Other | Africa | Cosmopolitan |
| KY210284 | 2012 | Tanzania | | Wild-Canis | Africa | Cosmopolitan |
| KY210285 | 2012 | Tanzania | | Other | Africa | Cosmopolitan |
| KY210286 | 2011 | Tanzania | | Canis-familiaris | Africa | Cosmopolitan |
| KY210287 | 2012 | Tanzania | | Canis-familiaris | Africa | Cosmopolitan |
| KY210288 | 2012 | Tanzania | | Canis-familiaris | Africa | Cosmopolitan |
| KY210289 | 2012 | Tanzania | | Canis-familiaris | Africa | Cosmopolitan |
| KY210290 | 2012 | Tanzania | | Canis-familiaris | Africa | Cosmopolitan |
| KY210291 | 2012 | Tanzania | | Wild-Canis | Africa | Cosmopolitan |
| KY210292 | 2012 | Tanzania | | Canis-familiaris | Africa | Cosmopolitan |
| KY210293 | 2012 | Tanzania | | Canis-familiaris | Africa | Cosmopolitan |
| KY210294 | 2012 | Tanzania | | Canis-familiaris | Africa | Cosmopolitan |
| KY210295 | 2012 | Tanzania | | Canis-familiaris | Africa | Cosmopolitan |
| KY210296 | 2012 | Tanzania | | Canis-familiaris | Africa | Cosmopolitan |
| KY210297 | 2012 | Tanzania | | Other | Africa | Cosmopolitan |
| KY210298 | 2012 | Tanzania | | Other | Africa | Cosmopolitan |
| KY210299 | 2012 | Tanzania | | Canis-familiaris | Africa | Cosmopolitan |
| KY210300 | 2012 | Tanzania | | Canis-familiaris | Africa | Cosmopolitan |
| KY210301 | 2012 | Tanzania | | Other | Africa | Cosmopolitan |
| KY210302 | 2012 | Tanzania | | Other | Africa | Cosmopolitan |
| KY210303 | 2012 | Tanzania | | Canis-familiaris | Africa | Cosmopolitan |
| KY210304 | 2012 | Tanzania | | Canis-familiaris | Africa | Cosmopolitan |
| KY210305 | 2012 | Tanzania | | Other | Africa | Cosmopolitan |
| KY210306 | 2012 | Tanzania | | Other | Africa | Cosmopolitan |
| KY210307 | 2012 | Tanzania | | Canis-familiaris | Africa | Cosmopolitan |
| KY210308 | 2012 | Tanzania | | Other | Africa | Cosmopolitan |
| KY210309 | 2012 | Tanzania | | Other | Africa | Cosmopolitan |
| KY210310 | 2012 | Tanzania | | Other | Africa | Cosmopolitan |
| KY210311 | 2012 | Tanzania | | Canis-familiaris | Africa | Cosmopolitan |
| KY451767 | 2015 | China | | Canis-familiaris | Asia | Asian |
| KY765901 | 2012 | Tajikistan | | Canis-familiaris | Asia | Cosmopolitan |
| KY775603 | 2014 | India | | Other | Asia | Arctic |
| KY775604 | 2014 | India | | Other | Asia | Arctic |
| KY780299 | 2017 | China | | Human | Asia | Asian |
| KY860583 | 1999 | Turkey | | Canis-familiaris | Europe | Cosmopolitan |
| KY860584 | 1999 | Turkey | | Wild-Canis | Europe | Cosmopolitan |
| KY860585 | 2001 | Turkey | | Wild-Canis | Europe | Cosmopolitan |
| KY860586 | 2001 | Turkey | | Wild-Canis | Europe | Cosmopolitan |
| KY860587 | 2001 | Turkey | | Wild-Canis | Europe | Cosmopolitan |
| KY860588 | 2004 | Turkey | | Wild-Canis | Europe | Cosmopolitan |
| KY860589 | 2006 | Turkey | | Wild-Canis | Europe | Cosmopolitan |
| KY860590 | 2007 | Turkey | | Wild-Canis | Europe | Cosmopolitan |
| KY860591 | 2008 | Turkey | | Wild-Canis | Europe | Cosmopolitan |
| KY860592 | 2009 | Turkey | | Wild-Canis | Europe | Cosmopolitan |
| KY860593 | 2010 | Turkey | | Wild-Canis | Europe | Cosmopolitan |
| KY860594 | 2012 | Turkey | | Wild-Canis | Europe | Cosmopolitan |
| KY860595 | 2012 | Turkey | | Wild-Canis | Europe | Cosmopolitan |
| KY860596 | 2014 | Turkey | | Wild-Canis | Europe | Cosmopolitan |
| KY860597 | 2014 | Turkey | | Canis-familiaris | Europe | Cosmopolitan |
| KY860598 | 2014 | Turkey | | Wild-Canis | Europe | Cosmopolitan |
| KY860599 | 2014 | Turkey | | Canis-familiaris | Europe | Cosmopolitan |
| KY860600 | 2014 | Turkey | | Wild-Canis | Europe | Cosmopolitan |
| KY860601 | 2014 | Turkey | | Wild-Canis | Europe | Cosmopolitan |
| KY860602 | 2014 | Turkey | | Canis-familiaris | Europe | Cosmopolitan |
| KY860603 | 2015 | Turkey | | Wild-Canis | Europe | Cosmopolitan |
| KY860604 | 2000 | Turkey | | Wild-Canis | Europe | Cosmopolitan |
| KY860605 | 2001 | Turkey | | Wild-Canis | Europe | Cosmopolitan |
| KY860606 | 2001 | Turkey | | Wild-Canis | Europe | Cosmopolitan |
| KY860607 | 2001 | Turkey | | Canis-familiaris | Europe | Cosmopolitan |
| KY860608 | 2001 | Turkey | | Canis-familiaris | Europe | Cosmopolitan |
| KY860609 | 2001 | Turkey | | Canis-familiaris | Europe | Cosmopolitan |
| KY860610 | 2001 | Turkey | | Canis-familiaris | Europe | Cosmopolitan |
| KY860611 | 2001 | Turkey | | Canis-familiaris | Europe | Cosmopolitan |
| KY860612 | 1989 | Turkey | | Canis-familiaris | Europe | Cosmopolitan |
| KY860613 | 1989 | Russia | | Canis-familiaris | Europe | Arctic |
| KY912036 | 2014 | China | | Other | Asia | Arctic |
| KY952219 | 2014 | China | | Wild-Canis | Asia | Arctic |
| KY952220 | 2016 | China | | Canis-familiaris | Asia | Arctic |
| KY964322 | 2016 | China | | Canis-familiaris | Asia | Arctic |
| KY964323 | 2016 | China | | Human | Asia | Arctic |
| KY982922 | 2014 | China | | Canis-familiaris | Asia | Arctic |
| KY982923 | 2012 | China | | Human | Asia | Arctic |
| KY997452 | 2014 | China | | Canis-familiaris | Asia | Arctic |
| LC029889 | 2009 | Uganda | | Other | Africa | Cosmopolitan |
| LC571945 | 2006 | Japan | | Human | Asia | Asian |
| LC717421 | 1965 | USA | | RAC-SK | North America | Cosmopolitan |
| LC717422 | 1963 | Thailand | | Canis-familiaris | Asia | Asian |
| LC717423 | 1985 | Thailand | | Canis-familiaris | Asia | Asian |
| LC717424 | 1985 | Thailand | | Canis-familiaris | Asia | Asian |
| LC717425 | 1985 | Thailand | | Canis-familiaris | Asia | Asian |
| LC717426 | 1976 | Thailand | | Canis-familiaris | Asia | Asian |
| LC717427 | 1977 | Thailand | | Other | Asia | Asian |
| LC717428 | 1977 | Thailand | | Canis-familiaris | Asia | Asian |
| LM645015 | 2006 | Greenland | | Wild-Canis | North America | Arctic |
| LM645016 | 2006 | Greenland | | Wild-Canis | North America | Arctic |
| LM645017 | 2005 | Greenland | | Wild-Canis | North America | Arctic |
| LM645018 | 2006 | Greenland | | Wild-Canis | North America | Arctic |
| LM645019 | 2010 | Greenland | | Wild-Canis | North America | Arctic |
| LM645020 | 2007 | Greenland | | Wild-Canis | North America | Arctic |
| LM645021 | 2006 | Greenland | | Wild-Canis | North America | Arctic |
| LM645022 | 2006 | Greenland | | Canis-familiaris | North America | Arctic |
| LM645023 | 2006 | Greenland | | Wild-Canis | North America | Arctic |
| LM645024 | 2006 | Greenland | | Wild-Canis | North America | Arctic |
| LM645025 | 2006 | Greenland | | Wild-Canis | North America | Arctic |
| LM645026 | 2006 | Greenland | | Wild-Canis | North America | Arctic |
| LM645027 | 2006 | Greenland | | Wild-Canis | North America | Arctic |
| LM645028 | 2006 | Greenland | | Wild-Canis | North America | Arctic |
| LM645029 | 2007 | Greenland | | Wild-Canis | North America | Arctic |
| LM645030 | 2007 | Greenland | | Wild-Canis | North America | Arctic |
| LM645031 | 2006 | Greenland | | Wild-Canis | North America | Arctic |
| LM645032 | 2010 | Greenland | | Wild-Canis | North America | Arctic |
| LM645033 | 2010 | Greenland | | Wild-Canis | North America | Arctic |
| LM645034 | 2009 | Greenland | | Wild-Canis | North America | Arctic |
| LM645035 | 2010 | Greenland | | Wild-Canis | North America | Arctic |
| LM645036 | 2010 | Greenland | | Wild-Canis | North America | Arctic |
| LM645037 | 2008 | Greenland | | Wild-Canis | North America | Arctic |
| LM645038 | 2009 | Greenland | | Wild-Canis | North America | Arctic |
| LM645039 | 2009 | Greenland | | Other | North America | Arctic |
| LM645040 | 2009 | Greenland | | Other | North America | Arctic |
| LM645041 | 2008 | Greenland | | Wild-Canis | North America | Arctic |
| LM645042 | 2009 | Greenland | | Wild-Canis | North America | Arctic |
| LM645043 | 2010 | Greenland | | Wild-Canis | North America | Arctic |
| LM645044 | 2011 | Greenland | | Other | North America | Arctic |
| LM645045 | 2007 | Greenland | | Wild-Canis | North America | Arctic |
| LM645046 | 2006 | Greenland | | Other | North America | Arctic |
| LM645047 | 2008 | Greenland | | Wild-Canis | North America | Arctic |
| LM645048 | 2005 | Greenland | | Wild-Canis | North America | Arctic |
| LM645049 | 2007 | Greenland | | Wild-Canis | North America | Arctic |
| LM645050 | 2007 | Greenland | | Wild-Canis | North America | Arctic |
| LM645051 | 2007 | Greenland | | Wild-Canis | North America | Arctic |
| LM645052 | 2006 | Greenland | | Wild-Canis | North America | Arctic |
| LM645053 | 2007 | Greenland | | Wild-Canis | North America | Arctic |
| LM645054 | 2007 | Greenland | | Other | North America | Arctic |
| LM645055 | 2006 | Greenland | | Wild-Canis | North America | Arctic |
| LM645056 | 2008 | Greenland | | Wild-Canis | North America | Arctic |
| LN879480 | 2002 | Azerbaijan | | Canis-familiaris | Asia | Cosmopolitan |
| LN879481 | 1998 | Germany | | Wild-Canis | Europe | Cosmopolitan |
| MF143191 | 2013 | USA | | RAC-SK | North America | RAC-SK |
| MF143192 | 2013 | USA | | RAC-SK | North America | RAC-SK |
| MF143193 | 2013 | USA | | RAC-SK | North America | RAC-SK |
| MF143194 | 2013 | USA | | RAC-SK | North America | RAC-SK |
| MF143195 | 2013 | USA | | RAC-SK | North America | RAC-SK |
| MF143196 | 2013 | USA | | RAC-SK | North America | RAC-SK |
| MF143197 | 2013 | USA | | RAC-SK | North America | RAC-SK |
| MF143198 | 2013 | USA | | RAC-SK | North America | RAC-SK |
| MF143199 | 2013 | USA | | RAC-SK | North America | RAC-SK |
| MF143200 | 2013 | USA | | RAC-SK | North America | RAC-SK |
| MF143201 | 2013 | USA | | RAC-SK | North America | RAC-SK |
| MF143202 | 2013 | USA | | RAC-SK | North America | RAC-SK |
| MF143203 | 2013 | USA | | Wild-Canis | North America | RAC-SK |
| MF143204 | 2013 | USA | | RAC-SK | North America | RAC-SK |
| MF143205 | 2013 | USA | | RAC-SK | North America | RAC-SK |
| MF143206 | 2013 | USA | | RAC-SK | North America | RAC-SK |
| MF143207 | 2013 | USA | | Wild-Canis | North America | RAC-SK |
| MF143208 | 2013 | USA | | RAC-SK | North America | RAC-SK |
| MF143209 | 2013 | USA | | RAC-SK | North America | RAC-SK |
| MF143210 | 2013 | USA | | RAC-SK | North America | RAC-SK |
| MF143211 | 2013 | USA | | RAC-SK | North America | RAC-SK |
| MF143212 | 2013 | USA | | RAC-SK | North America | RAC-SK |
| MF143213 | 2014 | USA | | RAC-SK | North America | RAC-SK |
| MF143214 | 2014 | USA | | RAC-SK | North America | RAC-SK |
| MF143215 | 2014 | USA | | RAC-SK | North America | RAC-SK |
| MF143216 | 2014 | USA | | RAC-SK | North America | RAC-SK |
| MF143217 | 2014 | USA | | RAC-SK | North America | RAC-SK |
| MF143218 | 2014 | USA | | RAC-SK | North America | RAC-SK |
| MF143219 | 2014 | USA | | RAC-SK | North America | RAC-SK |
| MF143220 | 2014 | USA | | Wild-Canis | North America | RAC-SK |
| MF143221 | 2014 | USA | | RAC-SK | North America | RAC-SK |
| MF143222 | 2014 | USA | | RAC-SK | North America | RAC-SK |
| MF143223 | 2000 | Canada | | RAC-SK | North America | RAC-SK |
| MF143224 | 2000 | Canada | | RAC-SK | North America | RAC-SK |
| MF143225 | 2000 | Canada | | RAC-SK | North America | RAC-SK |
| MF143226 | 2000 | Canada | | RAC-SK | North America | RAC-SK |
| MF143227 | 2000 | Canada | | RAC-SK | North America | RAC-SK |
| MF143228 | 2001 | Canada | | RAC-SK | North America | RAC-SK |
| MF143229 | 2001 | Canada | | RAC-SK | North America | RAC-SK |
| MF143230 | 2001 | Canada | | RAC-SK | North America | RAC-SK |
| MF143231 | 2001 | Canada | | RAC-SK | North America | RAC-SK |
| MF143232 | 2001 | Canada | | RAC-SK | North America | RAC-SK |
| MF143233 | 2001 | Canada | | RAC-SK | North America | RAC-SK |
| MF143234 | 2002 | Canada | | RAC-SK | North America | RAC-SK |
| MF143235 | 2014 | Canada | | RAC-SK | North America | RAC-SK |
| MF143236 | 2015 | Canada | | RAC-SK | North America | RAC-SK |
| MF143237 | 2015 | Canada | | RAC-SK | North America | RAC-SK |
| MF143238 | 2015 | Canada | | RAC-SK | North America | RAC-SK |
| MF143239 | 2015 | Canada | | RAC-SK | North America | RAC-SK |
| MF143240 | 2015 | Canada | | RAC-SK | North America | RAC-SK |
| MF143241 | 2015 | Canada | | RAC-SK | North America | RAC-SK |
| MF143242 | 2015 | Canada | | RAC-SK | North America | RAC-SK |
| MF143243 | 2015 | Canada | | RAC-SK | North America | RAC-SK |
| MF143244 | 2015 | Canada | | RAC-SK | North America | RAC-SK |
| MF143245 | 2015 | Canada | | RAC-SK | North America | RAC-SK |
| MF143246 | 2015 | Canada | | RAC-SK | North America | RAC-SK |
| MF143247 | 2015 | Canada | | RAC-SK | North America | RAC-SK |
| MF143248 | 2015 | Canada | | RAC-SK | North America | RAC-SK |
| MF143249 | 2015 | Canada | | RAC-SK | North America | RAC-SK |
| MF143250 | 2015 | Canada | | RAC-SK | North America | RAC-SK |
| MF143251 | 2015 | Canada | | RAC-SK | North America | RAC-SK |
| MF143252 | 2015 | Canada | | RAC-SK | North America | RAC-SK |
| MF143253 | 2015 | Canada | | RAC-SK | North America | RAC-SK |
| MF143254 | 2015 | Canada | | RAC-SK | North America | RAC-SK |
| MF143255 | 2015 | Canada | | RAC-SK | North America | RAC-SK |
| MF143256 | 2007 | Canada | | RAC-SK | North America | RAC-SK |
| MF143257 | 2004 | USA | | RAC-SK | North America | RAC-SK |
| MF143258 | 2004 | USA | | RAC-SK | North America | RAC-SK |
| MF143259 | 2007 | Canada | | RAC-SK | North America | RAC-SK |
| MF143260 | 2007 | Canada | | RAC-SK | North America | RAC-SK |
| MF143261 | 2007 | Canada | | RAC-SK | North America | RAC-SK |
| MF143262 | 2007 | Canada | | RAC-SK | North America | RAC-SK |
| MF143263 | 2007 | Canada | | RAC-SK | North America | RAC-SK |
| MF143264 | 2007 | Canada | | RAC-SK | North America | RAC-SK |
| MF143265 | 2007 | Canada | | RAC-SK | North America | RAC-SK |
| MF143266 | 2007 | Canada | | RAC-SK | North America | RAC-SK |
| MF143267 | 2007 | Canada | | RAC-SK | North America | RAC-SK |
| MF143268 | 2007 | Canada | | RAC-SK | North America | RAC-SK |
| MF143269 | 2007 | Canada | | RAC-SK | North America | RAC-SK |
| MF143270 | 2007 | Canada | | RAC-SK | North America | RAC-SK |
| MF143271 | 2007 | Canada | | RAC-SK | North America | RAC-SK |
| MF143272 | 2008 | Canada | | RAC-SK | North America | RAC-SK |
| MF143273 | 2008 | Canada | | RAC-SK | North America | RAC-SK |
| MF143274 | 2008 | Canada | | RAC-SK | North America | RAC-SK |
| MF143275 | 2008 | Canada | | RAC-SK | North America | RAC-SK |
| MF143276 | 2009 | Canada | | RAC-SK | North America | RAC-SK |
| MF143277 | 2015 | Canada | | RAC-SK | North America | RAC-SK |
| MF143278 | 2006 | USA | | Other | North America | RAC-SK |
| MF143279 | 2006 | USA | | RAC-SK | North America | RAC-SK |
| MF143280 | 2006 | USA | | RAC-SK | North America | RAC-SK |
| MF143281 | 2006 | USA | | RAC-SK | North America | RAC-SK |
| MF143282 | 2007 | USA | | RAC-SK | North America | RAC-SK |
| MF143283 | 2007 | USA | | RAC-SK | North America | RAC-SK |
| MF143284 | 2007 | USA | | RAC-SK | North America | RAC-SK |
| MF143285 | 2007 | USA | | RAC-SK | North America | RAC-SK |
| MF143286 | 2007 | USA | | RAC-SK | North America | RAC-SK |
| MF143287 | 2007 | USA | | RAC-SK | North America | RAC-SK |
| MF143288 | 2008 | USA | | RAC-SK | North America | RAC-SK |
| MF143289 | 2009 | USA | | RAC-SK | North America | RAC-SK |
| MF143290 | 1994 | USA | | RAC-SK | North America | RAC-SK |
| MF143291 | 1995 | USA | | RAC-SK | North America | RAC-SK |
| MF143292 | 2003 | USA | | RAC-SK | North America | RAC-SK |
| MF143293 | 2003 | USA | | RAC-SK | North America | RAC-SK |
| MF143294 | 2003 | USA | | RAC-SK | North America | RAC-SK |
| MF143295 | 2003 | USA | | RAC-SK | North America | RAC-SK |
| MF143296 | 2004 | USA | | RAC-SK | North America | RAC-SK |
| MF143297 | 2004 | USA | | RAC-SK | North America | RAC-SK |
| MF143298 | 2004 | USA | | RAC-SK | North America | RAC-SK |
| MF143299 | 2004 | USA | | RAC-SK | North America | RAC-SK |
| MF143300 | 2004 | USA | | RAC-SK | North America | RAC-SK |
| MF143301 | 2004 | USA | | RAC-SK | North America | RAC-SK |
| MF143302 | 2004 | USA | | RAC-SK | North America | RAC-SK |
| MF143303 | 2004 | USA | | RAC-SK | North America | RAC-SK |
| MF143304 | 2004 | USA | | RAC-SK | North America | RAC-SK |
| MF143305 | 2004 | USA | | RAC-SK | North America | RAC-SK |
| MF143306 | 2004 | USA | | RAC-SK | North America | RAC-SK |
| MF143307 | 2004 | USA | | RAC-SK | North America | RAC-SK |
| MF143308 | 2010 | USA | | RAC-SK | North America | RAC-SK |
| MF143309 | 2010 | USA | | RAC-SK | North America | RAC-SK |
| MF143310 | 2010 | USA | | RAC-SK | North America | RAC-SK |
| MF143311 | 2010 | USA | | RAC-SK | North America | RAC-SK |
| MF143312 | 2010 | USA | | RAC-SK | North America | RAC-SK |
| MF143313 | 2010 | USA | | RAC-SK | North America | RAC-SK |
| MF143314 | 2010 | USA | | RAC-SK | North America | RAC-SK |
| MF143315 | 2010 | USA | | RAC-SK | North America | RAC-SK |
| MF143316 | 2010 | USA | | RAC-SK | North America | RAC-SK |
| MF143317 | 2010 | USA | | RAC-SK | North America | RAC-SK |
| MF143318 | 2014 | USA | | RAC-SK | North America | RAC-SK |
| MF143319 | 2010 | USA | | RAC-SK | North America | RAC-SK |
| MF143320 | 2010 | USA | | RAC-SK | North America | RAC-SK |
| MF143321 | 2010 | USA | | RAC-SK | North America | RAC-SK |
| MF143322 | 2010 | USA | | RAC-SK | North America | RAC-SK |
| MF143323 | 2010 | USA | | RAC-SK | North America | RAC-SK |
| MF143324 | 2011 | USA | | RAC-SK | North America | RAC-SK |
| MF143325 | 2011 | USA | | RAC-SK | North America | RAC-SK |
| MF143326 | 2011 | USA | | RAC-SK | North America | RAC-SK |
| MF143327 | 2011 | USA | | Other | North America | RAC-SK |
| MF143328 | 2011 | USA | | RAC-SK | North America | RAC-SK |
| MF143329 | 2011 | USA | | RAC-SK | North America | RAC-SK |
| MF143330 | 2011 | USA | | RAC-SK | North America | RAC-SK |
| MF143331 | 2011 | USA | | RAC-SK | North America | RAC-SK |
| MF143332 | 2011 | USA | | RAC-SK | North America | RAC-SK |
| MF143333 | 2011 | USA | | Other | North America | RAC-SK |
| MF143334 | 2011 | USA | | RAC-SK | North America | RAC-SK |
| MF143335 | 2011 | USA | | Wild-Canis | North America | RAC-SK |
| MF143336 | 2003 | USA | | RAC-SK | North America | RAC-SK |
| MF143337 | 2007 | USA | | RAC-SK | North America | RAC-SK |
| MF143338 | 2009 | USA | | Other | North America | RAC-SK |
| MF143339 | 2010 | USA | | RAC-SK | North America | RAC-SK |
| MF143340 | 2011 | USA | | RAC-SK | North America | RAC-SK |
| MF143341 | 2006 | Canada | | RAC-SK | North America | RAC-SK |
| MF143342 | 2006 | Canada | | RAC-SK | North America | RAC-SK |
| MF143343 | 2006 | Canada | | RAC-SK | North America | RAC-SK |
| MF143344 | 2007 | Canada | | RAC-SK | North America | RAC-SK |
| MF143345 | 2007 | Canada | | RAC-SK | North America | RAC-SK |
| MF143346 | 2007 | Canada | | RAC-SK | North America | RAC-SK |
| MF143347 | 2007 | Canada | | RAC-SK | North America | RAC-SK |
| MF143348 | 2007 | Canada | | RAC-SK | North America | RAC-SK |
| MF143349 | 2007 | Canada | | RAC-SK | North America | RAC-SK |
| MF143350 | 2007 | Canada | | RAC-SK | North America | RAC-SK |
| MF143351 | 2007 | Canada | | RAC-SK | North America | RAC-SK |
| MF143352 | 2007 | Canada | | RAC-SK | North America | RAC-SK |
| MF143353 | 2007 | Canada | | RAC-SK | North America | RAC-SK |
| MF143354 | 2007 | Canada | | RAC-SK | North America | RAC-SK |
| MF143355 | 2007 | Canada | | RAC-SK | North America | RAC-SK |
| MF143356 | 2007 | Canada | | RAC-SK | North America | RAC-SK |
| MF143357 | 2007 | Canada | | RAC-SK | North America | RAC-SK |
| MF143358 | 2007 | Canada | | RAC-SK | North America | RAC-SK |
| MF143359 | 2007 | Canada | | Wild-Canis | North America | RAC-SK |
| MF143360 | 2007 | Canada | | RAC-SK | North America | RAC-SK |
| MF143361 | 2008 | Canada | | RAC-SK | North America | RAC-SK |
| MF143362 | 2008 | Canada | | RAC-SK | North America | RAC-SK |
| MF143363 | 2008 | Canada | | RAC-SK | North America | RAC-SK |
| MF143364 | 2008 | Canada | | RAC-SK | North America | RAC-SK |
| MF143365 | 2008 | Canada | | RAC-SK | North America | RAC-SK |
| MF143366 | 2008 | Canada | | RAC-SK | North America | RAC-SK |
| MF143367 | 2008 | Canada | | RAC-SK | North America | RAC-SK |
| MF143368 | 2008 | Canada | | RAC-SK | North America | RAC-SK |
| MF143369 | 2008 | Canada | | RAC-SK | North America | RAC-SK |
| MF143370 | 2008 | Canada | | RAC-SK | North America | RAC-SK |
| MF143371 | 2008 | Canada | | RAC-SK | North America | RAC-SK |
| MF143372 | 2009 | Canada | | RAC-SK | North America | RAC-SK |
| MF143373 | 2005 | USA | | RAC-SK | North America | RAC-SK |
| MF143374 | 2006 | USA | | RAC-SK | North America | RAC-SK |
| MF143375 | 2006 | USA | | RAC-SK | North America | RAC-SK |
| MF143376 | 2006 | USA | | RAC-SK | North America | RAC-SK |
| MF143377 | 2006 | USA | | RAC-SK | North America | RAC-SK |
| MF143378 | 2007 | USA | | RAC-SK | North America | RAC-SK |
| MF143379 | 2007 | USA | | RAC-SK | North America | RAC-SK |
| MF143380 | 2007 | USA | | RAC-SK | North America | RAC-SK |
| MF143381 | 2007 | USA | | RAC-SK | North America | RAC-SK |
| MF143382 | 2007 | USA | | RAC-SK | North America | RAC-SK |
| MF143383 | 2007 | USA | | RAC-SK | North America | RAC-SK |
| MF143384 | 2007 | USA | | RAC-SK | North America | RAC-SK |
| MF143385 | 2007 | USA | | RAC-SK | North America | RAC-SK |
| MF143386 | 2007 | USA | | RAC-SK | North America | RAC-SK |
| MF143387 | 2007 | USA | | RAC-SK | North America | RAC-SK |
| MF143388 | 2007 | USA | | RAC-SK | North America | RAC-SK |
| MF143389 | 2007 | USA | | RAC-SK | North America | RAC-SK |
| MF143390 | 2007 | USA | | RAC-SK | North America | RAC-SK |
| MF143391 | 2008 | USA | | Other | North America | RAC-SK |
| MF143392 | 2009 | USA | | RAC-SK | North America | RAC-SK |
| MF143393 | 2009 | USA | | RAC-SK | North America | RAC-SK |
| MF143394 | 2009 | USA | | RAC-SK | North America | RAC-SK |
| MF143395 | 2009 | USA | | RAC-SK | North America | RAC-SK |
| MF143396 | 2009 | USA | | RAC-SK | North America | RAC-SK |
| MF143397 | 2009 | USA | | RAC-SK | North America | RAC-SK |
| MF143398 | 2009 | USA | | RAC-SK | North America | RAC-SK |
| MF143399 | 2009 | USA | | RAC-SK | North America | RAC-SK |
| MF143400 | 2009 | USA | | RAC-SK | North America | RAC-SK |
| MF143401 | 2009 | USA | | RAC-SK | North America | RAC-SK |
| MF143402 | 2009 | USA | | RAC-SK | North America | RAC-SK |
| MF143403 | 2009 | USA | | Other | North America | RAC-SK |
| MF143404 | 2009 | USA | | RAC-SK | North America | RAC-SK |
| MF143405 | 2009 | USA | | RAC-SK | North America | RAC-SK |
| MF143406 | 2009 | USA | | Other | North America | RAC-SK |
| MF143407 | 2010 | USA | | RAC-SK | North America | RAC-SK |
| MF143408 | 2010 | USA | | RAC-SK | North America | RAC-SK |
| MF143409 | 2010 | USA | | RAC-SK | North America | RAC-SK |
| MF143410 | 2010 | USA | | RAC-SK | North America | RAC-SK |
| MF143411 | 2010 | USA | | RAC-SK | North America | RAC-SK |
| MF143412 | 2010 | USA | | RAC-SK | North America | RAC-SK |
| MF143413 | 2010 | USA | | RAC-SK | North America | RAC-SK |
| MF143414 | 2011 | USA | | RAC-SK | North America | RAC-SK |
| MF143415 | 2011 | USA | | RAC-SK | North America | RAC-SK |
| MF197741 | 2008 | Poland | | Wild-Canis | Europe | Cosmopolitan |
| MF197742 | 2014 | Poland | | Wild-Canis | Europe | Cosmopolitan |
| MF197743 | 2010 | Poland | | Wild-Canis | Europe | Cosmopolitan |
| MF476106 | 2015 | China | | Human | Asia | Asian |
| MG011654 | 2016 | France | | Human | Europe | Arctic |
| MG201919 | 2011 | China | | Canis-familiaris | Asia | Asian |
| MG201920 | 2010 | China | | Canis-familiaris | Asia | Asian |
| MG201921 | 2012 | China | | Canis-familiaris | Asia | Asian |
| MG201922 | 2012 | China | | Canis-familiaris | Asia | Asian |
| MG201923 | 2003 | China | | Canis-familiaris | Asia | Asian |
| MG458304 | 1975 | USA | | Bat | North America | Bats |
| MG458307 | 2000 | South Africa | | Other | Africa | Africa-3 |
| MG458308 | 2000 | South Africa | | Other | Africa | Africa-3 |
| MG458309 | 1978 | Serbia | | Canis-familiaris | Europe | Cosmopolitan |
| MG458310 | 1986 | Serbia | | Other | Europe | Cosmopolitan |
| MG458311 | 1998 | Serbia | | Wild-Canis | Europe | Cosmopolitan |
| MG458312 | 1997 | Serbia | | Wild-Canis | Europe | Cosmopolitan |
| MG458313 | 1996 | Russia | | Wild-Canis | Europe | Arctic |
| MG458315 | 1998 | Egypt | | Canis-familiaris | Africa | Cosmopolitan |
| MG458316 | 1998 | Egypt | | Canis-familiaris | Africa | Cosmopolitan |
| MG458317 | 1999 | Egypt | | Canis-familiaris | Africa | Cosmopolitan |
| MG458318 | 2008 | South Africa | | Human | Africa | Cosmopolitan |
| MG458319 | 2012 | Grenada | | Other | North America | Cosmopolitan |
| MG458320 | 2012 | Nepal | | Human | Asia | Arctic |
| MG562518 | 2003 | USA | | RAC-SK | North America | RAC-SK |
| MG562519 | 2003 | USA | | RAC-SK | North America | RAC-SK |
| MG562520 | 2003 | USA | | RAC-SK | North America | RAC-SK |
| MG562521 | 2004 | USA | | Other | North America | RAC-SK |
| MG562522 | 2003 | USA | | RAC-SK | North America | RAC-SK |
| MG562523 | 1990 | USA | | RAC-SK | North America | RAC-SK |
| MG562524 | 1990 | USA | | RAC-SK | North America | RAC-SK |
| MG562525 | 1992 | USA | | RAC-SK | North America | RAC-SK |
| MG562526 | 1995 | USA | | RAC-SK | North America | RAC-SK |
| MG562527 | 2003 | USA | | RAC-SK | North America | RAC-SK |
| MG562528 | 2003 | USA | | RAC-SK | North America | RAC-SK |
| MG562529 | 2003 | USA | | RAC-SK | North America | RAC-SK |
| MG562530 | 2003 | USA | | RAC-SK | North America | RAC-SK |
| MG562531 | 2003 | USA | | RAC-SK | North America | RAC-SK |
| MG562532 | 2003 | USA | | RAC-SK | North America | RAC-SK |
| MG562533 | 2003 | USA | | RAC-SK | North America | RAC-SK |
| MG562534 | 2004 | USA | | RAC-SK | North America | RAC-SK |
| MG562535 | 2004 | USA | | RAC-SK | North America | RAC-SK |
| MG562536 | 2004 | USA | | RAC-SK | North America | RAC-SK |
| MG562537 | 2004 | USA | | RAC-SK | North America | RAC-SK |
| MG562538 | 2004 | USA | | RAC-SK | North America | RAC-SK |
| MG562539 | 2004 | USA | | RAC-SK | North America | RAC-SK |
| MG562540 | 2004 | USA | | RAC-SK | North America | RAC-SK |
| MG562541 | 2004 | USA | | RAC-SK | North America | RAC-SK |
| MG562542 | 2004 | USA | | RAC-SK | North America | RAC-SK |
| MG562543 | 2010 | USA | | RAC-SK | North America | RAC-SK |
| MG562544 | 2010 | USA | | RAC-SK | North America | RAC-SK |
| MG562545 | 2010 | USA | | RAC-SK | North America | RAC-SK |
| MG562546 | 2010 | USA | | RAC-SK | North America | RAC-SK |
| MG562547 | 2011 | USA | | RAC-SK | North America | RAC-SK |
| MG562548 | 2011 | USA | | RAC-SK | North America | RAC-SK |
| MG562549 | 2011 | USA | | RAC-SK | North America | RAC-SK |
| MG562550 | 2011 | USA | | RAC-SK | North America | RAC-SK |
| MG562551 | 2011 | USA | | Wild-Canis | North America | RAC-SK |
| MG562552 | 2011 | USA | | Wild-Canis | North America | RAC-SK |
| MG562553 | 2011 | USA | | RAC-SK | North America | RAC-SK |
| MG562554 | 2011 | USA | | RAC-SK | North America | RAC-SK |
| MG562555 | 2005 | USA | | RAC-SK | North America | RAC-SK |
| MG562556 | 2005 | USA | | RAC-SK | North America | RAC-SK |
| MG562557 | 2005 | USA | | RAC-SK | North America | RAC-SK |
| MG562558 | 2005 | USA | | RAC-SK | North America | RAC-SK |
| MG562559 | 2005 | USA | | RAC-SK | North America | RAC-SK |
| MG562560 | 2005 | USA | | RAC-SK | North America | RAC-SK |
| MG562561 | 2005 | USA | | RAC-SK | North America | RAC-SK |
| MG562562 | 2005 | USA | | RAC-SK | North America | RAC-SK |
| MG562563 | 2005 | USA | | RAC-SK | North America | RAC-SK |
| MG562564 | 2006 | USA | | RAC-SK | North America | RAC-SK |
| MG562565 | 2006 | USA | | RAC-SK | North America | RAC-SK |
| MG562566 | 2006 | USA | | RAC-SK | North America | RAC-SK |
| MG562567 | 2006 | USA | | RAC-SK | North America | RAC-SK |
| MG562568 | 2006 | USA | | RAC-SK | North America | RAC-SK |
| MG562569 | 2006 | USA | | RAC-SK | North America | RAC-SK |
| MG562570 | 2006 | USA | | RAC-SK | North America | RAC-SK |
| MG562571 | 2006 | USA | | Other | North America | RAC-SK |
| MG562572 | 2006 | USA | | RAC-SK | North America | RAC-SK |
| MG562573 | 2006 | USA | | RAC-SK | North America | RAC-SK |
| MG562574 | 2006 | USA | | Other | North America | RAC-SK |
| MG562575 | 2007 | USA | | Other | North America | RAC-SK |
| MG562576 | 2007 | USA | | RAC-SK | North America | RAC-SK |
| MG562577 | 2007 | USA | | RAC-SK | North America | RAC-SK |
| MG562578 | 2007 | USA | | RAC-SK | North America | RAC-SK |
| MG562579 | 2007 | USA | | RAC-SK | North America | RAC-SK |
| MG562580 | 2008 | USA | | RAC-SK | North America | RAC-SK |
| MG562581 | 2008 | USA | | RAC-SK | North America | RAC-SK |
| MG562582 | 2008 | USA | | RAC-SK | North America | RAC-SK |
| MG562583 | 2008 | USA | | RAC-SK | North America | RAC-SK |
| MG562584 | 2008 | USA | | RAC-SK | North America | RAC-SK |
| MG562585 | 2008 | USA | | RAC-SK | North America | RAC-SK |
| MG562586 | 2008 | USA | | RAC-SK | North America | RAC-SK |
| MG562587 | 2008 | USA | | RAC-SK | North America | RAC-SK |
| MG562588 | 2008 | USA | | RAC-SK | North America | RAC-SK |
| MG562589 | 2008 | USA | | RAC-SK | North America | RAC-SK |
| MG562590 | 2008 | USA | | RAC-SK | North America | RAC-SK |
| MG562591 | 2008 | USA | | RAC-SK | North America | RAC-SK |
| MG562592 | 2008 | USA | | RAC-SK | North America | RAC-SK |
| MG562593 | 2009 | USA | | RAC-SK | North America | RAC-SK |
| MG562594 | 2009 | USA | | Other | North America | RAC-SK |
| MG562595 | 2009 | USA | | RAC-SK | North America | RAC-SK |
| MG562596 | 2009 | USA | | Wild-Canis | North America | RAC-SK |
| MG562597 | 2009 | USA | | RAC-SK | North America | RAC-SK |
| MG562598 | 2009 | USA | | RAC-SK | North America | RAC-SK |
| MG562599 | 2009 | USA | | RAC-SK | North America | RAC-SK |
| MG562600 | 2009 | USA | | RAC-SK | North America | RAC-SK |
| MG562601 | 2009 | USA | | RAC-SK | North America | RAC-SK |
| MG562602 | 2009 | USA | | RAC-SK | North America | RAC-SK |
| MG562603 | 2009 | USA | | Other | North America | RAC-SK |
| MG562604 | 2009 | USA | | RAC-SK | North America | RAC-SK |
| MG562605 | 2009 | USA | | RAC-SK | North America | RAC-SK |
| MG562606 | 2009 | USA | | RAC-SK | North America | RAC-SK |
| MG562607 | 2010 | USA | | Wild-Canis | North America | RAC-SK |
| MG562608 | 2010 | USA | | RAC-SK | North America | RAC-SK |
| MG562609 | 2010 | USA | | Wild-Canis | North America | RAC-SK |
| MG562610 | 2011 | USA | | RAC-SK | North America | RAC-SK |
| MH267792 | 2017 | China | | Other | Asia | Cosmopolitan |
| MH514968 | 2001 | Senegal | | Human | Africa | Africa-2 |
| MH514969 | 2005 | Senegal | | Human | Africa | Africa-2 |
| MH514970 | 2011 | Senegal | | Canis-familiaris | Africa | Africa-2 |
| MH514971 | 2010 | Senegal | | Canis-familiaris | Africa | Africa-2 |
| MH514972 | 2008 | Senegal | | Canis-familiaris | Africa | Africa-2 |
| MH514973 | 2011 | Senegal | | Other | Africa | Africa-2 |
| MH514974 | 2011 | Senegal | | Canis-familiaris | Africa | Africa-2 |
| MH514975 | 2013 | Senegal | | Canis-familiaris | Africa | Africa-2 |
| MH514976 | 2010 | Senegal | | Canis-familiaris | Africa | Africa-2 |
| MH514977 | 2013 | Senegal | | Canis-familiaris | Africa | Africa-2 |
| MH514978 | 2014 | Senegal | | Canis-familiaris | Africa | Africa-2 |
| MH514979 | 2014 | Senegal | | Canis-familiaris | Africa | Africa-2 |
| MH514980 | 2014 | Senegal | | Canis-familiaris | Africa | Africa-2 |
| MH514981 | 2013 | Senegal | | Canis-familiaris | Africa | Africa-2 |
| MH514982 | 2007 | Senegal | | Human | Africa | Africa-2 |
| MH514983 | 2011 | Senegal | | Canis-familiaris | Africa | Africa-2 |
| MH514984 | 2011 | Senegal | | Human | Africa | Africa-2 |
| MH514985 | 2015 | Senegal | | Canis-familiaris | Africa | Africa-2 |
| MH671332 | 2017 | China | | Human | Asia | Indian-Sub |
| MK111075 | 2015 | Hungary | | Wild-Canis | Europe | Cosmopolitan |
| MK540658 | 1990 | USA | | RAC-SK | North America | RAC-SK |
| MK540659 | 1990 | USA | | RAC-SK | North America | RAC-SK |
| MK540660 | 1990 | USA | | RAC-SK | North America | RAC-SK |
| MK540661 | 1990 | USA | | RAC-SK | North America | RAC-SK |
| MK540662 | 1990 | USA | | RAC-SK | North America | RAC-SK |
| MK540663 | 1990 | USA | | RAC-SK | North America | RAC-SK |
| MK540664 | 1990 | USA | | RAC-SK | North America | RAC-SK |
| MK540665 | 1990 | USA | | RAC-SK | North America | RAC-SK |
| MK540666 | 1990 | USA | | RAC-SK | North America | RAC-SK |
| MK540667 | 1990 | USA | | RAC-SK | North America | RAC-SK |
| MK540668 | 1990 | USA | | RAC-SK | North America | RAC-SK |
| MK540669 | 1990 | USA | | Other | North America | RAC-SK |
| MK540670 | 1990 | USA | | RAC-SK | North America | RAC-SK |
| MK540671 | 1990 | USA | | RAC-SK | North America | RAC-SK |
| MK540672 | 1990 | USA | | RAC-SK | North America | RAC-SK |
| MK540673 | 1990 | USA | | RAC-SK | North America | RAC-SK |
| MK540674 | 1990 | USA | | RAC-SK | North America | RAC-SK |
| MK540675 | 1990 | USA | | RAC-SK | North America | RAC-SK |
| MK540676 | 1990 | USA | | RAC-SK | North America | RAC-SK |
| MK540677 | 1990 | USA | | RAC-SK | North America | RAC-SK |
| MK540678 | 1991 | USA | | RAC-SK | North America | RAC-SK |
| MK540679 | 1991 | USA | | RAC-SK | North America | RAC-SK |
| MK540680 | 1991 | USA | | RAC-SK | North America | RAC-SK |
| MK540681 | 1991 | USA | | RAC-SK | North America | RAC-SK |
| MK540682 | 1992 | USA | | RAC-SK | North America | RAC-SK |
| MK540683 | 1992 | USA | | RAC-SK | North America | RAC-SK |
| MK540684 | 1992 | USA | | RAC-SK | North America | RAC-SK |
| MK540685 | 1992 | USA | | RAC-SK | North America | RAC-SK |
| MK540686 | 1992 | USA | | RAC-SK | North America | RAC-SK |
| MK540687 | 1992 | USA | | RAC-SK | North America | RAC-SK |
| MK540688 | 1993 | USA | | RAC-SK | North America | RAC-SK |
| MK540689 | 1993 | USA | | RAC-SK | North America | RAC-SK |
| MK540690 | 1995 | USA | | RAC-SK | North America | RAC-SK |
| MK540691 | 2003 | USA | | RAC-SK | North America | RAC-SK |
| MK540692 | 2003 | USA | | RAC-SK | North America | RAC-SK |
| MK540693 | 2003 | USA | | RAC-SK | North America | RAC-SK |
| MK540694 | 2003 | USA | | RAC-SK | North America | RAC-SK |
| MK540695 | 2003 | USA | | RAC-SK | North America | RAC-SK |
| MK540696 | 2004 | USA | | RAC-SK | North America | RAC-SK |
| MK540697 | 2004 | USA | | RAC-SK | North America | RAC-SK |
| MK540698 | 2004 | USA | | RAC-SK | North America | RAC-SK |
| MK540699 | 2004 | USA | | RAC-SK | North America | RAC-SK |
| MK540700 | 2004 | USA | | RAC-SK | North America | RAC-SK |
| MK540701 | 2004 | USA | | RAC-SK | North America | RAC-SK |
| MK540702 | 2004 | USA | | RAC-SK | North America | RAC-SK |
| MK540703 | 2004 | USA | | RAC-SK | North America | RAC-SK |
| MK540704 | 2004 | USA | | RAC-SK | North America | RAC-SK |
| MK540705 | 2004 | USA | | RAC-SK | North America | RAC-SK |
| MK540706 | 2004 | USA | | RAC-SK | North America | RAC-SK |
| MK540707 | 2004 | USA | | RAC-SK | North America | RAC-SK |
| MK540708 | 2004 | USA | | RAC-SK | North America | RAC-SK |
| MK540709 | 2004 | USA | | RAC-SK | North America | RAC-SK |
| MK540710 | 2004 | USA | | RAC-SK | North America | RAC-SK |
| MK540711 | 2004 | USA | | RAC-SK | North America | RAC-SK |
| MK540712 | 2004 | USA | | RAC-SK | North America | RAC-SK |
| MK540713 | 2004 | USA | | RAC-SK | North America | RAC-SK |
| MK540714 | 2004 | USA | | RAC-SK | North America | RAC-SK |
| MK540715 | 2004 | USA | | RAC-SK | North America | RAC-SK |
| MK540716 | 2004 | USA | | RAC-SK | North America | RAC-SK |
| MK540717 | 2004 | USA | | RAC-SK | North America | RAC-SK |
| MK540718 | 2004 | USA | | RAC-SK | North America | RAC-SK |
| MK540719 | 2004 | USA | | RAC-SK | North America | RAC-SK |
| MK540720 | 2004 | USA | | RAC-SK | North America | RAC-SK |
| MK540721 | 2004 | USA | | RAC-SK | North America | RAC-SK |
| MK540722 | 2009 | USA | | RAC-SK | North America | RAC-SK |
| MK540723 | 2010 | USA | | RAC-SK | North America | RAC-SK |
| MK540724 | 2010 | USA | | RAC-SK | North America | RAC-SK |
| MK540725 | 2010 | USA | | RAC-SK | North America | RAC-SK |
| MK540726 | 2010 | USA | | RAC-SK | North America | RAC-SK |
| MK540727 | 2010 | USA | | RAC-SK | North America | RAC-SK |
| MK540728 | 2010 | USA | | RAC-SK | North America | RAC-SK |
| MK540729 | 2010 | USA | | RAC-SK | North America | RAC-SK |
| MK540730 | 2010 | USA | | RAC-SK | North America | RAC-SK |
| MK540731 | 2010 | USA | | RAC-SK | North America | RAC-SK |
| MK540732 | 2010 | USA | | RAC-SK | North America | RAC-SK |
| MK540733 | 2010 | USA | | RAC-SK | North America | RAC-SK |
| MK540734 | 2010 | USA | | RAC-SK | North America | RAC-SK |
| MK540735 | 2010 | USA | | RAC-SK | North America | RAC-SK |
| MK540736 | 2010 | USA | | RAC-SK | North America | RAC-SK |
| MK540737 | 2010 | USA | | RAC-SK | North America | RAC-SK |
| MK540738 | 2010 | USA | | RAC-SK | North America | RAC-SK |
| MK540739 | 2010 | USA | | RAC-SK | North America | RAC-SK |
| MK540740 | 2010 | USA | | RAC-SK | North America | RAC-SK |
| MK540741 | 2010 | USA | | RAC-SK | North America | RAC-SK |
| MK540742 | 2011 | USA | | RAC-SK | North America | RAC-SK |
| MK540743 | 2011 | USA | | RAC-SK | North America | RAC-SK |
| MK540744 | 2011 | USA | | RAC-SK | North America | RAC-SK |
| MK540745 | 2011 | USA | | RAC-SK | North America | RAC-SK |
| MK540746 | 2011 | USA | | RAC-SK | North America | RAC-SK |
| MK540747 | 2011 | USA | | Wild-Canis | North America | RAC-SK |
| MK540748 | 2011 | USA | | RAC-SK | North America | RAC-SK |
| MK540749 | 2011 | USA | | RAC-SK | North America | RAC-SK |
| MK540750 | 2011 | USA | | RAC-SK | North America | RAC-SK |
| MK540751 | 2011 | USA | | RAC-SK | North America | RAC-SK |
| MK540752 | 2011 | USA | | RAC-SK | North America | RAC-SK |
| MK540753 | 2011 | USA | | RAC-SK | North America | RAC-SK |
| MK540754 | 2011 | USA | | RAC-SK | North America | RAC-SK |
| MK540755 | 2011 | USA | | RAC-SK | North America | RAC-SK |
| MK540756 | 2011 | USA | | RAC-SK | North America | RAC-SK |
| MK540757 | 2011 | USA | | Wild-Canis | North America | RAC-SK |
| MK540758 | 2011 | USA | | RAC-SK | North America | RAC-SK |
| MK540759 | 2011 | USA | | Other | North America | RAC-SK |
| MK540760 | 2011 | USA | | Other | North America | RAC-SK |
| MK540761 | 2011 | USA | | Other | North America | RAC-SK |
| MK540762 | 2011 | USA | | Other | North America | RAC-SK |
| MK540763 | 2011 | USA | | RAC-SK | North America | RAC-SK |
| MK540764 | 2011 | USA | | Wild-Canis | North America | RAC-SK |
| MK540765 | 2011 | USA | | RAC-SK | North America | RAC-SK |
| MK540766 | 2011 | USA | | Wild-Canis | North America | RAC-SK |
| MK540767 | 2011 | USA | | RAC-SK | North America | RAC-SK |
| MK540768 | 2011 | USA | | RAC-SK | North America | RAC-SK |
| MK540769 | 2011 | USA | | Other | North America | RAC-SK |
| MK540770 | 2011 | USA | | RAC-SK | North America | RAC-SK |
| MK540771 | 2011 | USA | | RAC-SK | North America | RAC-SK |
| MK540772 | 2011 | USA | | Other | North America | RAC-SK |
| MK540773 | 2011 | USA | | RAC-SK | North America | RAC-SK |
| MK540774 | 2011 | USA | | Other | North America | RAC-SK |
| MK540775 | 2011 | USA | | RAC-SK | North America | RAC-SK |
| MK540776 | 2011 | USA | | Wild-Canis | North America | RAC-SK |
| MK540777 | 2011 | USA | | Other | North America | RAC-SK |
| MK540778 | 2011 | USA | | RAC-SK | North America | RAC-SK |
| MK540779 | 2011 | USA | | RAC-SK | North America | RAC-SK |
| MK540780 | 2011 | USA | | RAC-SK | North America | RAC-SK |
| MK540781 | 2011 | USA | | Other | North America | RAC-SK |
| MK540782 | 2011 | USA | | RAC-SK | North America | RAC-SK |
| MK540783 | 2011 | USA | | Other | North America | RAC-SK |
| MK540784 | 2011 | USA | | RAC-SK | North America | RAC-SK |
| MK540785 | 2011 | USA | | RAC-SK | North America | RAC-SK |
| MK540786 | 2011 | USA | | Wild-Canis | North America | RAC-SK |
| MK540787 | 2011 | USA | | RAC-SK | North America | RAC-SK |
| MK540788 | 2011 | USA | | RAC-SK | North America | RAC-SK |
| MK540789 | 2011 | USA | | Wild-Canis | North America | RAC-SK |
| MK540790 | 2011 | USA | | Other | North America | RAC-SK |
| MK540791 | 2011 | USA | | Other | North America | RAC-SK |
| MK540792 | 2011 | USA | | RAC-SK | North America | RAC-SK |
| MK540793 | 2011 | USA | | RAC-SK | North America | RAC-SK |
| MK540794 | 2015 | USA | | RAC-SK | North America | RAC-SK |
| MK540795 | 2015 | USA | | RAC-SK | North America | RAC-SK |
| MK540796 | 2015 | USA | | RAC-SK | North America | RAC-SK |
| MK540797 | 2018 | USA | | RAC-SK | North America | RAC-SK |
| MK540798 | 2018 | USA | | RAC-SK | North America | RAC-SK |
| MK540799 | 2018 | USA | | RAC-SK | North America | RAC-SK |
| MK540800 | 2015 | Canada | | RAC-SK | North America | RAC-SK |
| MK540801 | 2015 | Canada | | RAC-SK | North America | RAC-SK |
| MK540802 | 2015 | Canada | | RAC-SK | North America | RAC-SK |
| MK540803 | 2015 | Canada | | RAC-SK | North America | RAC-SK |
| MK540804 | 2015 | Canada | | RAC-SK | North America | RAC-SK |
| MK540805 | 2016 | Canada | | RAC-SK | North America | RAC-SK |
| MK540806 | 2016 | Canada | | RAC-SK | North America | RAC-SK |
| MK540807 | 2016 | Canada | | RAC-SK | North America | RAC-SK |
| MK540808 | 2016 | Canada | | RAC-SK | North America | RAC-SK |
| MK540809 | 2016 | Canada | | RAC-SK | North America | RAC-SK |
| MK540810 | 2016 | Canada | | RAC-SK | North America | RAC-SK |
| MK540811 | 2016 | Canada | | RAC-SK | North America | RAC-SK |
| MK540812 | 2016 | Canada | | RAC-SK | North America | RAC-SK |
| MK540813 | 2016 | Canada | | RAC-SK | North America | RAC-SK |
| MK540814 | 2016 | Canada | | RAC-SK | North America | RAC-SK |
| MK540815 | 2016 | Canada | | RAC-SK | North America | RAC-SK |
| MK540816 | 2016 | Canada | | RAC-SK | North America | RAC-SK |
| MK540817 | 2016 | Canada | | RAC-SK | North America | RAC-SK |
| MK540818 | 2016 | Canada | | RAC-SK | North America | RAC-SK |
| MK540819 | 2016 | Canada | | RAC-SK | North America | RAC-SK |
| MK540820 | 2016 | Canada | | RAC-SK | North America | RAC-SK |
| MK540821 | 2016 | Canada | | RAC-SK | North America | RAC-SK |
| MK540822 | 2016 | Canada | | RAC-SK | North America | RAC-SK |
| MK540823 | 2016 | Canada | | RAC-SK | North America | RAC-SK |
| MK540824 | 2016 | Canada | | RAC-SK | North America | RAC-SK |
| MK540825 | 2016 | Canada | | RAC-SK | North America | RAC-SK |
| MK540826 | 2016 | Canada | | RAC-SK | North America | RAC-SK |
| MK540827 | 2016 | Canada | | RAC-SK | North America | RAC-SK |
| MK540828 | 2016 | Canada | | RAC-SK | North America | RAC-SK |
| MK540829 | 2016 | Canada | | RAC-SK | North America | RAC-SK |
| MK540830 | 2016 | Canada | | RAC-SK | North America | RAC-SK |
| MK540831 | 2016 | Canada | | RAC-SK | North America | RAC-SK |
| MK540832 | 2016 | Canada | | RAC-SK | North America | RAC-SK |
| MK540833 | 2016 | Canada | | RAC-SK | North America | RAC-SK |
| MK540834 | 2016 | Canada | | RAC-SK | North America | RAC-SK |
| MK540835 | 2016 | Canada | | RAC-SK | North America | RAC-SK |
| MK540836 | 2016 | Canada | | RAC-SK | North America | RAC-SK |
| MK540837 | 2016 | Canada | | RAC-SK | North America | RAC-SK |
| MK540838 | 2016 | Canada | | RAC-SK | North America | RAC-SK |
| MK540839 | 2016 | Canada | | RAC-SK | North America | RAC-SK |
| MK540840 | 2016 | Canada | | RAC-SK | North America | RAC-SK |
| MK540841 | 2016 | Canada | | RAC-SK | North America | RAC-SK |
| MK540842 | 2016 | Canada | | RAC-SK | North America | RAC-SK |
| MK540843 | 2016 | Canada | | Wild-Canis | North America | RAC-SK |
| MK540844 | 2016 | Canada | | RAC-SK | North America | RAC-SK |
| MK540845 | 2016 | Canada | | RAC-SK | North America | RAC-SK |
| MK540846 | 2016 | Canada | | RAC-SK | North America | RAC-SK |
| MK540847 | 2016 | Canada | | RAC-SK | North America | RAC-SK |
| MK540848 | 2016 | Canada | | RAC-SK | North America | RAC-SK |
| MK540849 | 2016 | Canada | | RAC-SK | North America | RAC-SK |
| MK540850 | 2016 | Canada | | RAC-SK | North America | RAC-SK |
| MK540851 | 2016 | Canada | | RAC-SK | North America | RAC-SK |
| MK540852 | 2016 | Canada | | RAC-SK | North America | RAC-SK |
| MK540853 | 2016 | Canada | | RAC-SK | North America | RAC-SK |
| MK540854 | 2016 | Canada | | RAC-SK | North America | RAC-SK |
| MK540855 | 2016 | Canada | | RAC-SK | North America | RAC-SK |
| MK540856 | 2016 | Canada | | RAC-SK | North America | RAC-SK |
| MK540857 | 2016 | Canada | | RAC-SK | North America | RAC-SK |
| MK540858 | 2016 | Canada | | RAC-SK | North America | RAC-SK |
| MK540859 | 2016 | Canada | | RAC-SK | North America | RAC-SK |
| MK540860 | 2016 | Canada | | RAC-SK | North America | RAC-SK |
| MK540861 | 2016 | Canada | | RAC-SK | North America | RAC-SK |
| MK540862 | 2016 | Canada | | RAC-SK | North America | RAC-SK |
| MK540863 | 2016 | Canada | | RAC-SK | North America | RAC-SK |
| MK540864 | 2016 | Canada | | RAC-SK | North America | RAC-SK |
| MK540865 | 2016 | Canada | | RAC-SK | North America | RAC-SK |
| MK540866 | 2016 | Canada | | RAC-SK | North America | RAC-SK |
| MK540867 | 2016 | Canada | | RAC-SK | North America | RAC-SK |
| MK540868 | 2016 | Canada | | RAC-SK | North America | RAC-SK |
| MK540869 | 2016 | Canada | | RAC-SK | North America | RAC-SK |
| MK540870 | 2016 | Canada | | RAC-SK | North America | RAC-SK |
| MK540871 | 2016 | Canada | | RAC-SK | North America | RAC-SK |
| MK540872 | 2017 | Canada | | RAC-SK | North America | RAC-SK |
| MK540873 | 2017 | Canada | | RAC-SK | North America | RAC-SK |
| MK540874 | 2017 | Canada | | RAC-SK | North America | RAC-SK |
| MK540875 | 2017 | Canada | | RAC-SK | North America | RAC-SK |
| MK540876 | 2017 | Canada | | RAC-SK | North America | RAC-SK |
| MK540877 | 2017 | Canada | | RAC-SK | North America | RAC-SK |
| MK540878 | 2017 | Canada | | RAC-SK | North America | RAC-SK |
| MK540879 | 2017 | Canada | | RAC-SK | North America | RAC-SK |
| MK540880 | 2017 | Canada | | RAC-SK | North America | RAC-SK |
| MK540881 | 2017 | Canada | | RAC-SK | North America | RAC-SK |
| MK540882 | 2017 | Canada | | RAC-SK | North America | RAC-SK |
| MK540883 | 1989 | USA | | RAC-SK | North America | RAC-SK |
| MK540884 | 1989 | USA | | RAC-SK | North America | RAC-SK |
| MK540885 | 1989 | USA | | RAC-SK | North America | RAC-SK |
| MK540886 | 1989 | USA | | RAC-SK | North America | RAC-SK |
| MK540887 | 1990 | Canada | | RAC-SK | North America | Arctic |
| MK540888 | 1990 | Canada | | Wild-Canis | North America | Arctic |
| MK540889 | 1990 | Canada | | Wild-Canis | North America | Arctic |
| MK540890 | 1990 | Canada | | RAC-SK | North America | Arctic |
| MK540891 | 1990 | Canada | | RAC-SK | North America | Arctic |
| MK540892 | 1990 | Canada | | RAC-SK | North America | Arctic |
| MK540893 | 1991 | Canada | | RAC-SK | North America | Arctic |
| MK540894 | 1991 | Canada | | RAC-SK | North America | Arctic |
| MK540895 | 1991 | Canada | | Wild-Canis | North America | Arctic |
| MK540896 | 1991 | Canada | | RAC-SK | North America | Arctic |
| MK540897 | 1991 | Canada | | RAC-SK | North America | Arctic |
| MK540898 | 1991 | Canada | | Wild-Canis | North America | Arctic |
| MK540899 | 1991 | Canada | | Wild-Canis | North America | Arctic |
| MK540900 | 1991 | Canada | | Wild-Canis | North America | Arctic |
| MK540901 | 1991 | Canada | | Wild-Canis | North America | Arctic |
| MK540902 | 1993 | Canada | | RAC-SK | North America | Arctic |
| MK540903 | 1993 | Canada | | Wild-Canis | North America | Arctic |
| MK540904 | 1993 | Canada | | Wild-Canis | North America | Arctic |
| MK540905 | 1993 | Canada | | Wild-Canis | North America | Arctic |
| MK540906 | 1995 | Canada | | Wild-Canis | North America | Arctic |
| MK540907 | 1995 | Canada | | RAC-SK | North America | Arctic |
| MK540908 | 1995 | Canada | | Wild-Canis | North America | Arctic |
| MK540909 | 1995 | Canada | | Wild-Canis | North America | Arctic |
| MK540910 | 1995 | Canada | | Wild-Canis | North America | Arctic |
| MK540911 | 1995 | Canada | | Wild-Canis | North America | Arctic |
| MK540912 | 1995 | Canada | | RAC-SK | North America | Arctic |
| MK540913 | 1996 | Canada | | RAC-SK | North America | Arctic |
| MK540914 | 1996 | Canada | | Wild-Canis | North America | Arctic |
| MK540915 | 1996 | Canada | | Wild-Canis | North America | Arctic |
| MK540916 | 1996 | Canada | | Wild-Canis | North America | Arctic |
| MK540917 | 1996 | Canada | | RAC-SK | North America | Arctic |
| MK540918 | 1996 | Canada | | RAC-SK | North America | Arctic |
| MK540919 | 1996 | Canada | | RAC-SK | North America | Arctic |
| MK540920 | 1997 | Canada | | RAC-SK | North America | Arctic |
| MK540921 | 1998 | Canada | | RAC-SK | North America | Arctic |
| MK540922 | 1998 | Canada | | RAC-SK | North America | Arctic |
| MK540923 | 1998 | Canada | | RAC-SK | North America | Arctic |
| MK540924 | 1998 | Canada | | RAC-SK | North America | Arctic |
| MK540925 | 1998 | Canada | | RAC-SK | North America | Arctic |
| MK540926 | 1998 | Canada | | RAC-SK | North America | Arctic |
| MK540927 | 1999 | Canada | | RAC-SK | North America | Arctic |
| MK540928 | 1999 | Canada | | RAC-SK | North America | Arctic |
| MK540929 | 1999 | Canada | | RAC-SK | North America | Arctic |
| MK540930 | 1999 | Canada | | Wild-Canis | North America | Arctic |
| MK540931 | 1999 | Canada | | RAC-SK | North America | Arctic |
| MK540932 | 1999 | Canada | | RAC-SK | North America | Arctic |
| MK540933 | 1999 | Canada | | RAC-SK | North America | Arctic |
| MK540934 | 1999 | Canada | | Wild-Canis | North America | Arctic |
| MK540935 | 1999 | Canada | | RAC-SK | North America | Arctic |
| MK540936 | 2000 | Canada | | RAC-SK | North America | Arctic |
| MK540937 | 2000 | Canada | | RAC-SK | North America | Arctic |
| MK540938 | 2000 | Canada | | RAC-SK | North America | Arctic |
| MK540939 | 2000 | Canada | | RAC-SK | North America | Arctic |
| MK540940 | 2000 | Canada | | RAC-SK | North America | Arctic |
| MK540941 | 2000 | Canada | | RAC-SK | North America | Arctic |
| MK540942 | 2000 | Canada | | RAC-SK | North America | Arctic |
| MK540943 | 2000 | Canada | | Wild-Canis | North America | Arctic |
| MK540944 | 2000 | Canada | | RAC-SK | North America | Arctic |
| MK540945 | 2000 | Canada | | RAC-SK | North America | Arctic |
| MK540946 | 2001 | Canada | | RAC-SK | North America | Arctic |
| MK540947 | 2001 | Canada | | RAC-SK | North America | Arctic |
| MK540948 | 2001 | Canada | | RAC-SK | North America | Arctic |
| MK540949 | 2001 | Canada | | RAC-SK | North America | Arctic |
| MK540950 | 2001 | Canada | | RAC-SK | North America | Arctic |
| MK540951 | 2001 | Canada | | RAC-SK | North America | Arctic |
| MK540952 | 2001 | Canada | | RAC-SK | North America | Arctic |
| MK540953 | 2001 | Canada | | RAC-SK | North America | Arctic |
| MK540954 | 2001 | Canada | | RAC-SK | North America | Arctic |
| MK540955 | 2001 | Canada | | RAC-SK | North America | Arctic |
| MK540956 | 2002 | Canada | | RAC-SK | North America | Arctic |
| MK540957 | 2002 | Canada | | RAC-SK | North America | Arctic |
| MK540958 | 2002 | Canada | | RAC-SK | North America | Arctic |
| MK540959 | 2002 | Canada | | RAC-SK | North America | Arctic |
| MK540960 | 2003 | Canada | | RAC-SK | North America | Arctic |
| MK540961 | 2003 | Canada | | RAC-SK | North America | Arctic |
| MK540962 | 2003 | Canada | | Wild-Canis | North America | Arctic |
| MK540963 | 2003 | Canada | | Wild-Canis | North America | Arctic |
| MK540964 | 2004 | Canada | | RAC-SK | North America | Arctic |
| MK540965 | 2004 | Canada | | RAC-SK | North America | Arctic |
| MK540966 | 2004 | Canada | | RAC-SK | North America | Arctic |
| MK540967 | 2005 | Canada | | Wild-Canis | North America | Arctic |
| MK540968 | 2005 | Canada | | RAC-SK | North America | Arctic |
| MK540969 | 2005 | Canada | | Wild-Canis | North America | Arctic |
| MK540970 | 2005 | Canada | | RAC-SK | North America | Arctic |
| MK540971 | 2006 | Canada | | RAC-SK | North America | Arctic |
| MK540972 | 2006 | Canada | | RAC-SK | North America | Arctic |
| MK540973 | 2006 | Canada | | Wild-Canis | North America | Arctic |
| MK540974 | 2006 | Canada | | RAC-SK | North America | Arctic |
| MK540975 | 2006 | Canada | | RAC-SK | North America | Arctic |
| MK540976 | 2006 | Canada | | RAC-SK | North America | Arctic |
| MK540977 | 2006 | Canada | | RAC-SK | North America | Arctic |
| MK540978 | 2007 | Canada | | RAC-SK | North America | Arctic |
| MK540979 | 2007 | Canada | | RAC-SK | North America | Arctic |
| MK540980 | 2007 | Canada | | RAC-SK | North America | Arctic |
| MK540981 | 2008 | Canada | | RAC-SK | North America | Arctic |
| MK540982 | 2008 | Canada | | RAC-SK | North America | Arctic |
| MK540983 | 2008 | Canada | | RAC-SK | North America | Arctic |
| MK540984 | 2008 | Canada | | RAC-SK | North America | Arctic |
| MK540985 | 2008 | Canada | | Wild-Canis | North America | Arctic |
| MK540986 | 2008 | Canada | | RAC-SK | North America | Arctic |
| MK540987 | 2009 | Canada | | Wild-Canis | North America | Arctic |
| MK540988 | 2009 | Canada | | Other | North America | Arctic |
| MK540989 | 2009 | Canada | | Other | North America | Arctic |
| MK540990 | 2009 | Canada | | RAC-SK | North America | Arctic |
| MK540991 | 2009 | Canada | | Wild-Canis | North America | Arctic |
| MK540992 | 2009 | Canada | | RAC-SK | North America | Arctic |
| MK540993 | 2010 | Canada | | RAC-SK | North America | Arctic |
| MK540994 | 2011 | Canada | | Other | North America | Arctic |
| MK540995 | 2011 | Canada | | RAC-SK | North America | Arctic |
| MK540996 | 2012 | Canada | | RAC-SK | North America | Arctic |
| MK540997 | 2012 | Canada | | Other | North America | Arctic |
| MK540998 | 2015 | Canada | | Other | North America | Arctic |
| MK540999 | 2016 | Canada | | Other | North America | Arctic |
| MK541000 | 2017 | Canada | | Other | North America | Arctic |
| MK541001 | 2017 | Canada | | Other | North America | Arctic |
| MK541002 | 2017 | Canada | | Other | North America | Arctic |
| MK541003 | 2017 | Canada | | Other | North America | Arctic |
| MK541004 | 2017 | Canada | | Wild-Canis | North America | Arctic |
| MK541005 | 2017 | Canada | | RAC-SK | North America | Arctic |
| MK541006 | 2017 | Canada | | RAC-SK | North America | Arctic |
| MK541007 | 2017 | Canada | | RAC-SK | North America | Arctic |
| MK541008 | 2017 | Canada | | RAC-SK | North America | Arctic |
| MK541009 | 2018 | Canada | | RAC-SK | North America | Arctic |
| MK541010 | 2018 | Canada | | RAC-SK | North America | Arctic |
| MK541011 | 2018 | Canada | | RAC-SK | North America | Arctic |
| MK541012 | 2000 | Canada | | Wild-Canis | North America | Arctic |
| MK567666 | 2013 | China | | Canis-familiaris | Asia | Asian |
| MK577649 | 2015 | China | | Other | Asia | Asian |
| MK598338 | 2006 | Hungary | | Wild-Canis | Europe | Cosmopolitan |
| MK598339 | 2007 | Hungary | | Wild-Canis | Europe | Cosmopolitan |
| MK598340 | 2007 | Hungary | | Wild-Canis | Europe | Cosmopolitan |
| MK598341 | 2008 | Hungary | | Wild-Canis | Europe | Cosmopolitan |
| MK598342 | 2009 | Hungary | | Wild-Canis | Europe | Cosmopolitan |
| MK598343 | 2008 | Hungary | | Wild-Canis | Europe | Cosmopolitan |
| MK598344 | 2009 | Hungary | | Wild-Canis | Europe | Cosmopolitan |
| MK598345 | 2010 | Hungary | | Wild-Canis | Europe | Cosmopolitan |
| MK598346 | 2010 | Hungary | | Wild-Canis | Europe | Cosmopolitan |
| MK598347 | 2016 | Hungary | | Wild-Canis | Europe | Cosmopolitan |
| MK598348 | 2017 | Hungary | | Wild-Canis | Europe | Cosmopolitan |
| MK598349 | 2017 | Hungary | | Other | Europe | Cosmopolitan |
| MK598350 | 2017 | Hungary | | Other | Europe | Cosmopolitan |
| MK598351 | 2013 | Hungary | | Wild-Canis | Europe | Cosmopolitan |
| MK598352 | 2013 | Hungary | | Wild-Canis | Europe | Cosmopolitan |
| MK598353 | 2013 | Hungary | | Wild-Canis | Europe | Cosmopolitan |
| MK598354 | 2013 | Hungary | | Wild-Canis | Europe | Cosmopolitan |
| MK598355 | 2013 | Hungary | | Wild-Canis | Europe | Cosmopolitan |
| MK598356 | 2013 | Hungary | | Wild-Canis | Europe | Cosmopolitan |
| MK598357 | 2013 | Hungary | | Wild-Canis | Europe | Cosmopolitan |
| MK598358 | 2013 | Hungary | | Wild-Canis | Europe | Cosmopolitan |
| MK598359 | 2013 | Hungary | | Wild-Canis | Europe | Cosmopolitan |
| MK598360 | 2013 | Hungary | | Wild-Canis | Europe | Cosmopolitan |
| MK598361 | 2013 | Hungary | | Wild-Canis | Europe | Cosmopolitan |
| MK598362 | 2013 | Hungary | | Other | Europe | Cosmopolitan |
| MK598363 | 2013 | Hungary | | Wild-Canis | Europe | Cosmopolitan |
| MK598364 | 2013 | Hungary | | Other | Europe | Cosmopolitan |
| MK598365 | 2014 | Hungary | | Wild-Canis | Europe | Cosmopolitan |
| MK598366 | 2014 | Hungary | | Wild-Canis | Europe | Cosmopolitan |
| MK598367 | 2014 | Hungary | | Wild-Canis | Europe | Cosmopolitan |
| MK598368 | 2014 | Hungary | | Wild-Canis | Europe | Cosmopolitan |
| MK598369 | 2014 | Hungary | | Wild-Canis | Europe | Cosmopolitan |
| MK598370 | 2014 | Hungary | | Wild-Canis | Europe | Cosmopolitan |
| MK598371 | 2014 | Hungary | | Wild-Canis | Europe | Cosmopolitan |
| MK598372 | 2014 | Hungary | | Wild-Canis | Europe | Cosmopolitan |
| MK598373 | 2014 | Hungary | | Wild-Canis | Europe | Cosmopolitan |
| MK598374 | 2014 | Hungary | | Wild-Canis | Europe | Cosmopolitan |
| MK598375 | 2014 | Hungary | | Wild-Canis | Europe | Cosmopolitan |
| MK598376 | 2014 | Hungary | | Wild-Canis | Europe | Cosmopolitan |
| MK598377 | 2014 | Hungary | | Wild-Canis | Europe | Cosmopolitan |
| MK598378 | 2014 | Hungary | | Other | Europe | Cosmopolitan |
| MK598379 | 2014 | Hungary | | Canis-familiaris | Europe | Cosmopolitan |
| MK598380 | 2013 | Hungary | | Wild-Canis | Europe | Cosmopolitan |
| MK598381 | 2013 | Hungary | | Wild-Canis | Europe | Cosmopolitan |
| MK598382 | 2013 | Hungary | | Wild-Canis | Europe | Cosmopolitan |
| MK598383 | 2013 | Hungary | | Wild-Canis | Europe | Cosmopolitan |
| MK598384 | 2013 | Hungary | | Wild-Canis | Europe | Cosmopolitan |
| MK598385 | 2013 | Hungary | | Wild-Canis | Europe | Cosmopolitan |
| MK598386 | 2013 | Hungary | | Wild-Canis | Europe | Cosmopolitan |
| MK598387 | 2013 | Hungary | | Wild-Canis | Europe | Cosmopolitan |
| MK598388 | 2013 | Hungary | | Wild-Canis | Europe | Cosmopolitan |
| MK598389 | 2013 | Hungary | | Wild-Canis | Europe | Cosmopolitan |
| MK598390 | 2014 | Hungary | | Other | Europe | Cosmopolitan |
| MK598391 | 2014 | Hungary | | Wild-Canis | Europe | Cosmopolitan |
| MK598392 | 2014 | Hungary | | Wild-Canis | Europe | Cosmopolitan |
| MK598393 | 2014 | Hungary | | Wild-Canis | Europe | Cosmopolitan |
| MK598394 | 2014 | Hungary | | Wild-Canis | Europe | Cosmopolitan |
| MK598395 | 2014 | Hungary | | Wild-Canis | Europe | Cosmopolitan |
| MK598396 | 2014 | Hungary | | Wild-Canis | Europe | Cosmopolitan |
| MK598397 | 2014 | Hungary | | Wild-Canis | Europe | Cosmopolitan |
| MK598398 | 1996 | Hungary | | Other | Europe | Cosmopolitan |
| MK689674 | 2017 | China | | Canis-familiaris | Asia | Asian |
| MK689675 | 2017 | China | | Canis-familiaris | Asia | Asian |
| MK689676 | 2017 | China | | Canis-familiaris | Asia | Asian |
| MK760667 | 2008 | Iran | | Wild-Canis | Asia | Cosmopolitan |
| MK760668 | 2008 | Iran | | Canis-familiaris | Asia | Cosmopolitan |
| MK760669 | 2008 | Iran | | Wild-Canis | Asia | Cosmopolitan |
| MK760670 | 2008 | Iran | | Canis-familiaris | Asia | Cosmopolitan |
| MK760671 | 2008 | Iran | | Wild-Canis | Asia | Cosmopolitan |
| MK760672 | 2008 | Iran | | Other | Asia | Cosmopolitan |
| MK760673 | 2008 | Iran | | Wild-Canis | Asia | Cosmopolitan |
| MK760674 | 2008 | Iran | | Wild-Canis | Asia | Cosmopolitan |
| MK760675 | 2008 | Iran | | Canis-familiaris | Asia | Cosmopolitan |
| MK760676 | 2009 | Iran | | Canis-familiaris | Asia | Cosmopolitan |
| MK760677 | 2009 | Iran | | Canis-familiaris | Asia | Cosmopolitan |
| MK760678 | 2009 | Iran | | Wild-Canis | Asia | Cosmopolitan |
| MK760679 | 2008 | Iran | | Canis-familiaris | Asia | Cosmopolitan |
| MK760680 | 2008 | Iran | | Canis-familiaris | Asia | Cosmopolitan |
| MK760681 | 2009 | Iran | | Canis-familiaris | Asia | Cosmopolitan |
| MK760682 | 2009 | Iran | | Canis-familiaris | Asia | Cosmopolitan |
| MK760683 | 2009 | Iran | | Canis-familiaris | Asia | Cosmopolitan |
| MK760684 | 2009 | Iran | | Canis-familiaris | Asia | Cosmopolitan |
| MK760685 | 2009 | Iran | | Canis-familiaris | Asia | Cosmopolitan |
| MK760686 | 2009 | Iran | | Canis-familiaris | Asia | Cosmopolitan |
| MK760687 | 2009 | Iran | | Canis-familiaris | Asia | Cosmopolitan |
| MK760688 | 2009 | Iran | | Wild-Canis | Asia | Cosmopolitan |
| MK760689 | 2010 | Iran | | Wild-Canis | Asia | Cosmopolitan |
| MK760690 | 2010 | Iran | | Canis-familiaris | Asia | Cosmopolitan |
| MK760691 | 2009 | Iran | | Canis-familiaris | Asia | Cosmopolitan |
| MK760692 | 2009 | Iran | | Wild-Canis | Asia | Cosmopolitan |
| MK760693 | 2009 | Iran | | Canis-familiaris | Asia | Cosmopolitan |
| MK760694 | 2009 | Iran | | Canis-familiaris | Asia | Cosmopolitan |
| MK760695 | 2011 | Iran | | Wild-Canis | Asia | Cosmopolitan |
| MK760696 | 2012 | Iran | | Canis-familiaris | Asia | Cosmopolitan |
| MK760697 | 2012 | Iran | | Canis-familiaris | Asia | Cosmopolitan |
| MK760698 | 2012 | Iran | | Wild-Canis | Asia | Cosmopolitan |
| MK760699 | 2012 | Iran | | Wild-Canis | Asia | Cosmopolitan |
| MK760700 | 2012 | Iran | | Wild-Canis | Asia | Cosmopolitan |
| MK760701 | 2012 | Iran | | Wild-Canis | Asia | Cosmopolitan |
| MK760702 | 2012 | Iran | | Canis-familiaris | Asia | Arctic |
| MK760703 | 2012 | Iran | | Wild-Canis | Asia | Cosmopolitan |
| MK760704 | 2012 | Iran | | Canis-familiaris | Asia | Cosmopolitan |
| MK760705 | 2012 | Iran | | Canis-familiaris | Asia | Cosmopolitan |
| MK760706 | 2012 | Iran | | Wild-Canis | Asia | Cosmopolitan |
| MK760707 | 2012 | Iran | | Canis-familiaris | Asia | Cosmopolitan |
| MK760708 | 2012 | Iran | | Wild-Canis | Asia | Cosmopolitan |
| MK760709 | 2012 | Iran | | Canis-familiaris | Asia | Cosmopolitan |
| MK760710 | 2013 | Iran | | Canis-familiaris | Asia | Cosmopolitan |
| MK760711 | 2013 | Iran | | Canis-familiaris | Asia | Cosmopolitan |
| MK760712 | 2013 | Iran | | Wild-Canis | Asia | Cosmopolitan |
| MK760713 | 2013 | Iran | | Canis-familiaris | Asia | Cosmopolitan |
| MK760714 | 2013 | Iran | | Canis-familiaris | Asia | Cosmopolitan |
| MK760715 | 2013 | Iran | | Wild-Canis | Asia | Cosmopolitan |
| MK760716 | 2013 | Iran | | Canis-familiaris | Asia | Cosmopolitan |
| MK760717 | 2013 | Iran | | Canis-familiaris | Asia | Cosmopolitan |
| MK760718 | 2013 | Iran | | Canis-familiaris | Asia | Cosmopolitan |
| MK760719 | 2013 | Iran | | Wild-Canis | Asia | Cosmopolitan |
| MK760720 | 2013 | Iran | | Wild-Canis | Asia | Cosmopolitan |
| MK760721 | 2013 | Iran | | Canis-familiaris | Asia | Cosmopolitan |
| MK760722 | 2013 | Iran | | Canis-familiaris | Asia | Cosmopolitan |
| MK760723 | 2013 | Iran | | Canis-familiaris | Asia | Cosmopolitan |
| MK760724 | 2013 | Iran | | Wild-Canis | Asia | Cosmopolitan |
| MK760725 | 2013 | Iran | | Canis-familiaris | Asia | Cosmopolitan |
| MK760726 | 2013 | Iran | | Wild-Canis | Asia | Cosmopolitan |
| MK760727 | 2013 | Iran | | Wild-Canis | Asia | Cosmopolitan |
| MK760728 | 2014 | Iran | | Canis-familiaris | Asia | Cosmopolitan |
| MK760729 | 2014 | Iran | | Canis-familiaris | Asia | Arctic |
| MK760730 | 2014 | Iran | | Canis-familiaris | Asia | Cosmopolitan |
| MK760731 | 2014 | Iran | | Canis-familiaris | Asia | Cosmopolitan |
| MK760732 | 2014 | Iran | | Canis-familiaris | Asia | Cosmopolitan |
| MK760733 | 2014 | Iran | | Canis-familiaris | Asia | Cosmopolitan |
| MK760734 | 2013 | Iran | | Wild-Canis | Asia | Cosmopolitan |
| MK760735 | 2014 | Iran | | Canis-familiaris | Asia | Arctic |
| MK760736 | 2014 | Iran | | Canis-familiaris | Asia | Cosmopolitan |
| MK760737 | 2013 | Iran | | Wild-Canis | Asia | Cosmopolitan |
| MK760738 | 2014 | Iran | | Canis-familiaris | Asia | Cosmopolitan |
| MK760739 | 2013 | Iran | | Wild-Canis | Asia | Cosmopolitan |
| MK760740 | 2013 | Iran | | Canis-familiaris | Asia | Cosmopolitan |
| MK760741 | 2013 | Iran | | Canis-familiaris | Asia | Cosmopolitan |
| MK760742 | 2014 | Iran | | Canis-familiaris | Asia | Cosmopolitan |
| MK760743 | 2014 | Iran | | Canis-familiaris | Asia | Cosmopolitan |
| MK760744 | 2014 | Iran | | Wild-Canis | Asia | Cosmopolitan |
| MK760745 | 2014 | Iran | | Canis-familiaris | Asia | Cosmopolitan |
| MK760746 | 2014 | Iran | | Canis-familiaris | Asia | Cosmopolitan |
| MK760747 | 2014 | Iran | | Wild-Canis | Asia | Cosmopolitan |
| MK760748 | 2014 | Iran | | Wild-Canis | Asia | Cosmopolitan |
| MK760749 | 2014 | Iran | | Canis-familiaris | Asia | Cosmopolitan |
| MK760750 | 2014 | Iran | | Other | Asia | Cosmopolitan |
| MK760751 | 2014 | Iran | | Canis-familiaris | Asia | Cosmopolitan |
| MK760752 | 2014 | Iran | | Wild-Canis | Asia | Cosmopolitan |
| MK760753 | 2014 | Iran | | Wild-Canis | Asia | Cosmopolitan |
| MK760754 | 2014 | Iran | | Canis-familiaris | Asia | Cosmopolitan |
| MK760755 | 2014 | Iran | | Canis-familiaris | Asia | Cosmopolitan |
| MK760756 | 2014 | Iran | | Canis-familiaris | Asia | Cosmopolitan |
| MK760757 | 2014 | Iran | | Canis-familiaris | Asia | Cosmopolitan |
| MK760758 | 2014 | Iran | | Canis-familiaris | Asia | Cosmopolitan |
| MK760759 | 2014 | Iran | | Wild-Canis | Asia | Cosmopolitan |
| MK760760 | 2014 | Iran | | Canis-familiaris | Asia | Cosmopolitan |
| MK760761 | 2014 | Iran | | Canis-familiaris | Asia | Arctic |
| MK760762 | 2014 | Iran | | Wild-Canis | Asia | Cosmopolitan |
| MK760763 | 2014 | Iran | | Wild-Canis | Asia | Cosmopolitan |
| MK760764 | 2014 | Iran | | Canis-familiaris | Asia | Cosmopolitan |
| MK760765 | 2014 | Iran | | Canis-familiaris | Asia | Cosmopolitan |
| MK760766 | 2015 | Iran | | Canis-familiaris | Asia | Cosmopolitan |
| MK760767 | 2015 | Iran | | Wild-Canis | Asia | Cosmopolitan |
| MK760768 | 2007 | Egypt | | Canis-familiaris | Africa | Cosmopolitan |
| MK760769 | 2009 | Egypt | | Other | Africa | Cosmopolitan |
| MK760770 | 2009 | Egypt | | Canis-familiaris | Africa | Cosmopolitan |
| MK920923 | 2010 | Brazil | | RAC-SK | South America | Bats |
| MK981888 | 1993 | Tunisia | | Canis-familiaris | Africa | Cosmopolitan |
| MN075931 | 1999 | Thailand | | Canis-familiaris | Asia | Asian |
| MN175989 | 2019 | China | | Other | Asia | Asian |
| MN186249 | 2018 | China | | Canis-familiaris | Asia | Asian |
| MN186250 | 2018 | China | | Canis-familiaris | Asia | Asian |
| MN233898 | 1989 | USA | | Wild-Canis | North America | Arctic |
| MN233899 | 2007 | USA | | Wild-Canis | North America | Arctic |
| MN233900 | 2007 | USA | | Wild-Canis | North America | Arctic |
| MN233901 | 2008 | USA | | Wild-Canis | North America | Arctic |
| MN233902 | 2008 | USA | | Wild-Canis | North America | Arctic |
| MN233903 | 2005 | Canada | | Wild-Canis | North America | Arctic |
| MN233904 | 2012 | Canada | | Wild-Canis | North America | Arctic |
| MN233905 | 2012 | Canada | | Canis-familiaris | North America | Arctic |
| MN233906 | 1996 | Canada | | Wild-Canis | North America | Arctic |
| MN233907 | 1996 | Canada | | Wild-Canis | North America | Arctic |
| MN233908 | 1996 | Canada | | Wild-Canis | North America | Arctic |
| MN233909 | 1996 | Canada | | Wild-Canis | North America | Arctic |
| MN233910 | 1996 | Canada | | Wild-Canis | North America | Arctic |
| MN233911 | 1996 | Canada | | Wild-Canis | North America | Arctic |
| MN233912 | 1996 | Canada | | Wild-Canis | North America | Arctic |
| MN233913 | 2002 | Canada | | Wild-Canis | North America | Arctic |
| MN233914 | 2002 | Canada | | Wild-Canis | North America | Arctic |
| MN233915 | 2002 | Canada | | Wild-Canis | North America | Arctic |
| MN233916 | 2003 | Canada | | Wild-Canis | North America | Arctic |
| MN233917 | 2003 | Canada | | Wild-Canis | North America | Arctic |
| MN233918 | 2003 | Canada | | Wild-Canis | North America | Arctic |
| MN233919 | 2003 | Canada | | Wild-Canis | North America | Arctic |
| MN233920 | 2004 | Canada | | Wild-Canis | North America | Arctic |
| MN233921 | 2004 | Canada | | Wild-Canis | North America | Arctic |
| MN233922 | 2004 | Canada | | Wild-Canis | North America | Arctic |
| MN233923 | 2004 | Canada | | Canis-familiaris | North America | Arctic |
| MN233924 | 2012 | Canada | | Wild-Canis | North America | Arctic |
| MN233925 | 2012 | Canada | | Wild-Canis | North America | Arctic |
| MN233926 | 2012 | Canada | | Wild-Canis | North America | Arctic |
| MN233927 | 2012 | Canada | | Wild-Canis | North America | Arctic |
| MN233928 | 2012 | Canada | | Wild-Canis | North America | Arctic |
| MN233929 | 2012 | Canada | | Wild-Canis | North America | Arctic |
| MN233930 | 2012 | Canada | | Wild-Canis | North America | Arctic |
| MN233931 | 2012 | Canada | | Wild-Canis | North America | Arctic |
| MN233932 | 2012 | Canada | | Wild-Canis | North America | Arctic |
| MN233933 | 2012 | Canada | | Wild-Canis | North America | Arctic |
| MN233934 | 2012 | Canada | | Wild-Canis | North America | Arctic |
| MN233935 | 2012 | Canada | | Wild-Canis | North America | Arctic |
| MN233936 | 2012 | Canada | | Wild-Canis | North America | Arctic |
| MN233937 | 2015 | Canada | | Wild-Canis | North America | Arctic |
| MN233938 | 2015 | Canada | | Wild-Canis | North America | Arctic |
| MN233939 | 2015 | Canada | | Canis-familiaris | North America | Arctic |
| MN233940 | 2015 | Canada | | Wild-Canis | North America | Arctic |
| MN233941 | 2015 | Canada | | Wild-Canis | North America | Arctic |
| MN233942 | 2015 | Canada | | Wild-Canis | North America | Arctic |
| MN233943 | 2015 | Canada | | Wild-Canis | North America | Arctic |
| MN233944 | 2015 | Canada | | Wild-Canis | North America | Arctic |
| MN233945 | 2015 | Canada | | Wild-Canis | North America | Arctic |
| MN233946 | 2015 | Canada | | Other | North America | Arctic |
| MN233947 | 1990 | Canada | | Other | North America | Arctic |
| MN233948 | 1990 | Canada | | Wild-Canis | North America | Arctic |
| MN233949 | 1990 | Canada | | Wild-Canis | North America | Arctic |
| MN233950 | 1992 | Canada | | Wild-Canis | North America | Arctic |
| MN233951 | 1993 | Canada | | Wild-Canis | North America | Arctic |
| MN233952 | 1993 | Canada | | Wild-Canis | North America | Arctic |
| MN233953 | 1993 | Canada | | Wild-Canis | North America | Arctic |
| MN233954 | 1993 | Canada | | Wild-Canis | North America | Arctic |
| MN233955 | 1993 | Canada | | Wild-Canis | North America | Arctic |
| MN233956 | 1993 | Canada | | Wild-Canis | North America | Arctic |
| MN233957 | 1993 | Canada | | Wild-Canis | North America | Arctic |
| MN233958 | 1994 | Canada | | Wild-Canis | North America | Arctic |
| MN233959 | 1997 | Canada | | Wild-Canis | North America | Arctic |
| MN233960 | 1998 | Canada | | Wild-Canis | North America | Arctic |
| MN233961 | 2000 | Canada | | Wild-Canis | North America | Arctic |
| MN233962 | 2000 | Canada | | Wild-Canis | North America | Arctic |
| MN233963 | 2001 | Canada | | Wild-Canis | North America | Arctic |
| MN233964 | 2005 | Canada | | Canis-familiaris | North America | Arctic |
| MN233965 | 2006 | Canada | | Wild-Canis | North America | Arctic |
| MN233966 | 2006 | Canada | | Wild-Canis | North America | Arctic |
| MN233967 | 2007 | Canada | | Wild-Canis | North America | Arctic |
| MN233968 | 2008 | Canada | | Wild-Canis | North America | Arctic |
| MN233969 | 2008 | Canada | | Wild-Canis | North America | Arctic |
| MN233970 | 2012 | Canada | | Wild-Canis | North America | Arctic |
| MN233971 | 2012 | Canada | | Canis-familiaris | North America | Arctic |
| MN233972 | 2012 | Canada | | Wild-Canis | North America | Arctic |
| MN233973 | 2013 | Canada | | Wild-Canis | North America | Arctic |
| MN233974 | 1999 | Canada | | Wild-Canis | North America | Arctic |
| MN233975 | 1999 | Canada | | Wild-Canis | North America | Arctic |
| MN233976 | 2002 | Canada | | Wild-Canis | North America | Arctic |
| MN233977 | 2002 | Canada | | Wild-Canis | North America | Arctic |
| MN233978 | 2004 | Canada | | Wild-Canis | North America | Arctic |
| MN233979 | 2005 | Canada | | Wild-Canis | North America | Arctic |
| MN233980 | 2005 | Canada | | Wild-Canis | North America | Arctic |
| MN233981 | 2005 | Canada | | Wild-Canis | North America | Arctic |
| MN233982 | 2006 | Canada | | Wild-Canis | North America | Arctic |
| MN233983 | 2006 | Canada | | Wild-Canis | North America | Arctic |
| MN233984 | 2006 | Canada | | Wild-Canis | North America | Arctic |
| MN233985 | 2007 | Canada | | Wild-Canis | North America | Arctic |
| MN233986 | 2007 | Canada | | Wild-Canis | North America | Arctic |
| MN233987 | 2007 | Canada | | Wild-Canis | North America | Arctic |
| MN233988 | 2008 | Canada | | Wild-Canis | North America | Arctic |
| MN233989 | 2008 | Canada | | Wild-Canis | North America | Arctic |
| MN233990 | 2012 | Canada | | Wild-Canis | North America | Arctic |
| MN233991 | 2012 | Canada | | Wild-Canis | North America | Arctic |
| MN233992 | 2012 | Canada | | Wild-Canis | North America | Arctic |
| MN233993 | 2012 | Canada | | Wild-Canis | North America | Arctic |
| MN233994 | 2013 | Canada | | Wild-Canis | North America | Arctic |
| MN233995 | 2013 | Canada | | Wild-Canis | North America | Arctic |
| MN233996 | 2013 | Canada | | Canis-familiaris | North America | Arctic |
| MN233997 | 2013 | Canada | | Canis-familiaris | North America | Arctic |
| MN233998 | 2014 | Canada | | Wild-Canis | North America | Arctic |
| MN233999 | 2015 | Canada | | Wild-Canis | North America | Arctic |
| MN234000 | 2015 | Canada | | Wild-Canis | North America | Arctic |
| MN234001 | 2015 | Canada | | Wild-Canis | North America | Arctic |
| MN234002 | 2015 | Canada | | Wild-Canis | North America | Arctic |
| MN234003 | 2015 | Canada | | Wild-Canis | North America | Arctic |
| MN234004 | 2015 | Canada | | Wild-Canis | North America | Arctic |
| MN234005 | 2015 | Canada | | Canis-familiaris | North America | Arctic |
| MN234006 | 2015 | Canada | | Wild-Canis | North America | Arctic |
| MN234007 | 2016 | Canada | | Wild-Canis | North America | Arctic |
| MN234008 | 2017 | Canada | | Wild-Canis | North America | Arctic |
| MN234009 | 2017 | Canada | | Wild-Canis | North America | Arctic |
| MN234010 | 2017 | Canada | | Wild-Canis | North America | Arctic |
| MN234011 | 2017 | Canada | | Canis-familiaris | North America | Arctic |
| MN234012 | 2017 | Canada | | Canis-familiaris | North America | Arctic |
| MN234013 | 1995 | Canada | | Wild-Canis | North America | Arctic |
| MN234014 | 2000 | Canada | | Wild-Canis | North America | Arctic |
| MN234015 | 2013 | Canada | | Canis-familiaris | North America | Arctic |
| MN234016 | 1993 | Canada | | Wild-Canis | North America | Arctic |
| MN234017 | 1996 | Canada | | Wild-Canis | North America | Arctic |
| MN234018 | 1999 | Canada | | Wild-Canis | North America | Arctic |
| MN234019 | 2000 | Canada | | Canis-familiaris | North America | Arctic |
| MN234020 | 2000 | Canada | | Canis-familiaris | North America | Arctic |
| MN234021 | 2000 | Canada | | Wild-Canis | North America | Arctic |
| MN234022 | 2001 | Canada | | Wild-Canis | North America | Arctic |
| MN234023 | 2002 | Canada | | Wild-Canis | North America | Arctic |
| MN234024 | 2003 | Canada | | Canis-familiaris | North America | Arctic |
| MN234025 | 2003 | Canada | | Wild-Canis | North America | Arctic |
| MN234026 | 2003 | Canada | | Wild-Canis | North America | Arctic |
| MN234027 | 2004 | Canada | | Wild-Canis | North America | Arctic |
| MN234028 | 2004 | Canada | | Wild-Canis | North America | Arctic |
| MN234029 | 2004 | Canada | | Wild-Canis | North America | Arctic |
| MN234030 | 2004 | Canada | | Wild-Canis | North America | Arctic |
| MN234031 | 2009 | Canada | | Wild-Canis | North America | Arctic |
| MN234032 | 2009 | Canada | | Wild-Canis | North America | Arctic |
| MN234033 | 2009 | Canada | | Canis-familiaris | North America | Arctic |
| MN234034 | 2009 | Canada | | Canis-familiaris | North America | Arctic |
| MN234035 | 2009 | Canada | | Canis-familiaris | North America | Arctic |
| MN234036 | 2012 | Canada | | Wild-Canis | North America | Arctic |
| MN234037 | 2012 | Canada | | Canis-familiaris | North America | Arctic |
| MN234038 | 2012 | Canada | | Wild-Canis | North America | Arctic |
| MN234039 | 2012 | Canada | | Canis-familiaris | North America | Arctic |
| MN234040 | 2012 | Canada | | Wild-Canis | North America | Arctic |
| MN234041 | 2012 | Canada | | Canis-familiaris | North America | Arctic |
| MN234042 | 2012 | Canada | | Wild-Canis | North America | Arctic |
| MN234043 | 2012 | Canada | | Wild-Canis | North America | Arctic |
| MN234044 | 2012 | Canada | | Wild-Canis | North America | Arctic |
| MN234045 | 2012 | Canada | | Wild-Canis | North America | Arctic |
| MN234046 | 2012 | Canada | | Wild-Canis | North America | Arctic |
| MN234047 | 2012 | Canada | | Canis-familiaris | North America | Arctic |
| MN234048 | 2012 | Canada | | Wild-Canis | North America | Arctic |
| MN234049 | 2012 | Canada | | Wild-Canis | North America | Arctic |
| MN234050 | 2012 | Canada | | Wild-Canis | North America | Arctic |
| MN234051 | 2015 | Canada | | Canis-familiaris | North America | Arctic |
| MN234052 | 2015 | Canada | | Wild-Canis | North America | Arctic |
| MN234053 | 2015 | Canada | | Canis-familiaris | North America | Arctic |
| MN234054 | 2015 | Canada | | Wild-Canis | North America | Arctic |
| MN234055 | 2017 | Canada | | Wild-Canis | North America | Arctic |
| MN234056 | 2017 | Canada | | Canis-familiaris | North America | Arctic |
| MN418142 | 2018 | USA | | RAC-SK | North America | RAC-SK |
| MN418143 | 2008 | USA | | RAC-SK | North America | RAC-SK |
| MN418144 | 2017 | USA | | RAC-SK | North America | RAC-SK |
| MN418145 | 2017 | USA | | RAC-SK | North America | RAC-SK |
| MN418146 | 2017 | USA | | RAC-SK | North America | RAC-SK |
| MN418147 | 2017 | USA | | RAC-SK | North America | RAC-SK |
| MN418148 | 2017 | USA | | RAC-SK | North America | RAC-SK |
| MN418149 | 2017 | USA | | Wild-Canis | North America | RAC-SK |
| MN418150 | 2016 | USA | | RAC-SK | North America | RAC-SK |
| MN418151 | 2016 | USA | | RAC-SK | North America | RAC-SK |
| MN418152 | 2016 | USA | | RAC-SK | North America | RAC-SK |
| MN418153 | 2017 | USA | | RAC-SK | North America | RAC-SK |
| MN418154 | 2017 | USA | | Other | North America | RAC-SK |
| MN418155 | 2016 | USA | | RAC-SK | North America | RAC-SK |
| MN418156 | 2018 | USA | | RAC-SK | North America | RAC-SK |
| MN418157 | 2017 | USA | | RAC-SK | North America | RAC-SK |
| MN418158 | 2017 | USA | | Wild-Canis | North America | RAC-SK |
| MN418159 | 2016 | USA | | RAC-SK | North America | RAC-SK |
| MN418160 | 2017 | USA | | RAC-SK | North America | RAC-SK |
| MN418161 | 2017 | USA | | Bat | North America | RAC-SK |
| MN418162 | 2017 | USA | | RAC-SK | North America | RAC-SK |
| MN418163 | 2016 | USA | | RAC-SK | North America | RAC-SK |
| MN418164 | 2009 | USA | | RAC-SK | North America | RAC-SK |
| MN418165 | 2017 | USA | | Wild-Canis | North America | RAC-SK |
| MN418166 | 2018 | USA | | Canis-familiaris | North America | Arctic |
| MN418167 | 2017 | USA | | RAC-SK | North America | RAC-SK |
| MN418168 | 2017 | USA | | RAC-SK | North America | RAC-SK |
| MN418169 | 2018 | USA | | RAC-SK | North America | RAC-SK |
| MN418170 | 2017 | USA | | Other | North America | RAC-SK |
| MN418171 | 2018 | USA | | RAC-SK | North America | RAC-SK |
| MN418172 | 2017 | USA | | RAC-SK | North America | RAC-SK |
| MN418173 | 2018 | USA | | RAC-SK | North America | RAC-SK |
| MN418174 | 2017 | USA | | RAC-SK | North America | RAC-SK |
| MN418175 | 2017 | USA | | RAC-SK | North America | RAC-SK |
| MN418176 | 2017 | USA | | RAC-SK | North America | RAC-SK |
| MN418177 | 2017 | USA | | Wild-Canis | North America | RAC-SK |
| MN418178 | 2017 | USA | | RAC-SK | North America | RAC-SK |
| MN418179 | 2017 | USA | | RAC-SK | North America | RAC-SK |
| MN418180 | 2017 | USA | | RAC-SK | North America | RAC-SK |
| MN418181 | 2016 | USA | | RAC-SK | North America | RAC-SK |
| MN418182 | 2016 | USA | | RAC-SK | North America | RAC-SK |
| MN418183 | 2017 | USA | | Wild-Canis | North America | RAC-SK |
| MN418184 | 2017 | USA | | RAC-SK | North America | RAC-SK |
| MN534894 | 2018 | Nepal | | Human | Asia | Indian-Sub |
| MN534896 | 2018 | Qatar | | Other | Asia | Cosmopolitan |
| MN534897 | 2018 | Qatar | | Wild-Canis | Asia | Cosmopolitan |
| MN726804 | 2015 | Tanzania | | Canis-familiaris | Africa | Cosmopolitan |
| MN726805 | 2017 | Kenya | | Other | Africa | Cosmopolitan |
| MN726812 | 2017 | Kenya | | Canis-familiaris | Africa | Cosmopolitan |
| MN726814 | 2018 | Kenya | | Other | Africa | Cosmopolitan |
| MN726815 | 2015 | Tanzania | | Wild-Canis | Africa | Cosmopolitan |
| MN726818 | 2015 | Tanzania | | Canis-familiaris | Africa | Cosmopolitan |
| MN726819 | 2013 | Kenya | | Human | Africa | Cosmopolitan |
| MN726826 | 2016 | Tanzania | | Canis-familiaris | Africa | Cosmopolitan |
| MN726827 | 2017 | Tanzania | | Canis-familiaris | Africa | Cosmopolitan |
| MN726830 | 2018 | Kenya | | Other | Africa | Cosmopolitan |
| MN726831 | 2013 | Kenya | | Other | Africa | Cosmopolitan |
| MN726836 | 2013 | Philippines | | Canis-familiaris | Asia | Asian |
| MN726839 | 2015 | Philippines | | Canis-familiaris | Asia | Asian |
| MN726841 | 2014 | Philippines | | Canis-familiaris | Asia | Asian |
| MN726861 | 2015 | Philippines | | Canis-familiaris | Asia | Asian |
| MN726873 | 2014 | Philippines | | Canis-familiaris | Asia | Asian |
| MN726879 | 2012 | Philippines | | Canis-familiaris | Asia | Asian |
| MN857167 | 2019 | Philippines | | Canis-familiaris | Asia | Asian |
| MN857168 | 2019 | Philippines | | Canis-familiaris | Asia | Asian |
| MN857169 | 2019 | Philippines | | Canis-familiaris | Asia | Asian |
| MN857170 | 2019 | Philippines | | Canis-familiaris | Asia | Asian |
| MN857171 | 2019 | Philippines | | Other | Asia | Asian |
| MN862283 | 1991 | USA | | RAC-SK | North America | RAC-SK |
| MT454631 | 2015 | South Africa | | Canis-familiaris | Africa | Cosmopolitan |
| MT454632 | 2015 | South Africa | | Wild-Canis | Africa | Cosmopolitan |
| MT454633 | 2015 | South Africa | | Wild-Canis | Africa | Cosmopolitan |
| MT454634 | 2015 | South Africa | | Canis-familiaris | Africa | Cosmopolitan |
| MT454635 | 2015 | South Africa | | Canis-familiaris | Africa | Cosmopolitan |
| MT454636 | 2016 | South Africa | | Canis-familiaris | Africa | Cosmopolitan |
| MT454637 | 2016 | South Africa | | Canis-familiaris | Africa | Cosmopolitan |
| MT454638 | 2016 | South Africa | | Wild-Canis | Africa | Cosmopolitan |
| MT454639 | 2016 | South Africa | | Canis-familiaris | Africa | Cosmopolitan |
| MT454640 | 2016 | South Africa | | Wild-Canis | Africa | Cosmopolitan |
| MT454641 | 2016 | South Africa | | Canis-familiaris | Africa | Cosmopolitan |
| MT454642 | 2016 | South Africa | | Wild-Canis | Africa | Cosmopolitan |
| MT454643 | 2017 | South Africa | | Canis-familiaris | Africa | Cosmopolitan |
| MT454644 | 2017 | South Africa | | Canis-familiaris | Africa | Cosmopolitan |
| MT454645 | 2017 | South Africa | | Canis-familiaris | Africa | Cosmopolitan |
| MT454646 | 2017 | South Africa | | Wild-Canis | Africa | Cosmopolitan |
| MT454647 | 2017 | South Africa | | Wild-Canis | Africa | Cosmopolitan |
| MT454648 | 2017 | South Africa | | Wild-Canis | Africa | Cosmopolitan |
| MT454649 | 2017 | South Africa | | Wild-Canis | Africa | Cosmopolitan |
| MT454650 | 2017 | South Africa | | Wild-Canis | Africa | Cosmopolitan |
| MT454651 | 2017 | South Africa | | Wild-Canis | Africa | Cosmopolitan |
| MT454652 | 2017 | South Africa | | Wild-Canis | Africa | Cosmopolitan |
| MT454653 | 2017 | South Africa | | Wild-Canis | Africa | Cosmopolitan |
| MT454654 | 2017 | South Africa | | Wild-Canis | Africa | Cosmopolitan |
| MW177594 | 2016 | Moldova | | Other | Europe | Cosmopolitan |
| OK564514 | 2021 | Mexico | | Other | North America | Cosmopolitan |
| OL440112 | 2015 | Romania | | Other | Europe | Cosmopolitan |
| OL449092 | 2014 | Romania | | Wild-Canis | Europe | Cosmopolitan |
| OL449093 | 2014 | Romania | | Wild-Canis | Europe | Cosmopolitan |
| OL449095 | 2012 | Romania | | Wild-Canis | Europe | Cosmopolitan |
| OL515134 | 2013 | Romania | | Wild-Canis | Europe | Cosmopolitan |
| OL515135 | 2013 | Romania | | Canis-familiaris | Europe | Cosmopolitan |
| OL515137 | 2012 | Romania | | Wild-Canis | Europe | Cosmopolitan |
| OL515138 | 2012 | Romania | | Canis-familiaris | Europe | Cosmopolitan |
| OL515139 | 2012 | Romania | | Other | Europe | Cosmopolitan |
| OL515140 | 2012 | Romania | | Other | Europe | Cosmopolitan |
| OL515141 | 2016 | Romania | | Wild-Canis | Europe | Cosmopolitan |
| OL515144 | 2014 | Romania | | Wild-Canis | Europe | Cosmopolitan |
| OL515145 | 2016 | Romania | | Wild-Canis | Europe | Cosmopolitan |
| OL515150 | 2016 | Moldova | | Other | Europe | Cosmopolitan |
| OM021440 | 2016 | Moldova | | Canis-familiaris | Europe | Cosmopolitan |
| OM021441 | 2016 | Romania | | Wild-Canis | Europe | Cosmopolitan |
| OM203138 | 2017 | Moldova | | Other | Europe | Cosmopolitan |
| OM203141 | 2016 | Moldova | | Other | Europe | Cosmopolitan |
| OM542185 | 2018 | Poland | | Wild-Canis | Europe | Cosmopolitan |
| OM542186 | 2019 | Poland | | Wild-Canis | Europe | Cosmopolitan |
| OM542187 | 2020 | Poland | | Wild-Canis | Europe | Cosmopolitan |
| OM542188 | 2020 | Poland | | Wild-Canis | Europe | Cosmopolitan |
| OM542189 | 2020 | Poland | | Wild-Canis | Europe | Cosmopolitan |
| OM542190 | 2020 | Poland | | Wild-Canis | Europe | Cosmopolitan |
| OM542191 | 2020 | Poland | | Canis-familiaris | Europe | Cosmopolitan |
| OM542192 | 2020 | Poland | | Wild-Canis | Europe | Cosmopolitan |
| OM542193 | 2021 | Poland | | Wild-Canis | Europe | Cosmopolitan |
| OM542194 | 2021 | Poland | | Wild-Canis | Europe | Cosmopolitan |
| OM542195 | 2021 | Poland | | Wild-Canis | Europe | Cosmopolitan |
| OM542196 | 2001 | Poland | | Wild-Canis | Europe | Cosmopolitan |
| OM542197 | 2000 | Poland | | Wild-Canis | Europe | Cosmopolitan |
| OM542198 | 2004 | Poland | | Other | Europe | Cosmopolitan |
| OM542199 | 2021 | Poland | | Wild-Canis | Europe | Cosmopolitan |
| OM542200 | 2021 | Poland | | Wild-Canis | Europe | Cosmopolitan |
| OM542201 | 2021 | Poland | | Other | Europe | Cosmopolitan |
| OM542202 | 2021 | Poland | | Wild-Canis | Europe | Cosmopolitan |
| OM542203 | 2021 | Poland | | Wild-Canis | Europe | Cosmopolitan |
| OM542204 | 2021 | Poland | | Other | Europe | Cosmopolitan |
| OM542205 | 2021 | Poland | | Wild-Canis | Europe | Cosmopolitan |
| OM542206 | 2021 | Poland | | Wild-Canis | Europe | Cosmopolitan |
| OM863568 | 2020 | India | | Other | Asia | Arctic |
| OM863569 | 2021 | India | | Other | Asia | Arctic |
| OM891790 | 2016 | India | | Other | Asia | Arctic |
| OM891791 | 2020 | India | | Other | Asia | Arctic |
| OM891792 | 2021 | India | | Other | Asia | Arctic |
| OM971001 | 2007 | Mexico | | RAC-SK | North America | Bats |
| OM971002 | 2008 | Mexico | | RAC-SK | North America | Bats |
| OM971003 | 2015 | Mexico | | RAC-SK | North America | Cosmopolitan |
| OM971004 | 2017 | Mexico | | RAC-SK | North America | Cosmopolitan |
| OM971005 | 2020 | Mexico | | RAC-SK | North America | Cosmopolitan |
| ON366706 | 2021 | Kazakhstan | | Other | Asia | Cosmopolitan |
| ON366707 | 2021 | Kazakhstan | | Other | Asia | Cosmopolitan |
| ON366708 | 2021 | Kazakhstan | | Other | Asia | Cosmopolitan |
| ON366709 | 2021 | Kazakhstan | | Other | Asia | Cosmopolitan |
| ON366710 | 2021 | Kazakhstan | | Other | Asia | Cosmopolitan |
| ON986424 | 2019 | USA | | Other | North America | RAC-SK |
| ON986425 | 2019 | USA | | Other | North America | RAC-SK |
| ON986426 | 2019 | USA | | Other | North America | RAC-SK |
| ON986427 | 2020 | USA | | Other | North America | RAC-SK |
| ON986428 | 2017 | USA | | Other | North America | RAC-SK |
| ON986429 | 2018 | USA | | Other | North America | RAC-SK |
| ON986430 | 2018 | USA | | Other | North America | RAC-SK |
| ON986432 | 2018 | USA | | Wild-Canis | North America | RAC-SK |
| ON986433 | 2017 | USA | | RAC-SK | North America | RAC-SK |
| ON986434 | 2017 | USA | | RAC-SK | North America | RAC-SK |
| ON986435 | 2017 | USA | | RAC-SK | North America | RAC-SK |
| ON986436 | 2017 | USA | | RAC-SK | North America | RAC-SK |
| ON986437 | 2017 | USA | | RAC-SK | North America | RAC-SK |
| ON986438 | 2017 | USA | | RAC-SK | North America | RAC-SK |
| ON986439 | 2017 | USA | | RAC-SK | North America | RAC-SK |
| ON986440 | 2017 | USA | | RAC-SK | North America | RAC-SK |
| ON986441 | 2017 | USA | | RAC-SK | North America | RAC-SK |
| ON986442 | 2018 | USA | | RAC-SK | North America | RAC-SK |
| ON986443 | 2018 | USA | | RAC-SK | North America | RAC-SK |
| ON986444 | 2018 | USA | | RAC-SK | North America | RAC-SK |
| ON986445 | 2018 | USA | | RAC-SK | North America | RAC-SK |
| ON986446 | 2018 | USA | | RAC-SK | North America | RAC-SK |
| ON986447 | 2018 | USA | | RAC-SK | North America | RAC-SK |
| ON986448 | 2018 | USA | | RAC-SK | North America | RAC-SK |
| ON986449 | 2018 | USA | | RAC-SK | North America | RAC-SK |
| ON986450 | 2018 | USA | | RAC-SK | North America | RAC-SK |
| ON986451 | 2018 | USA | | RAC-SK | North America | RAC-SK |
| ON986452 | 2018 | USA | | RAC-SK | North America | RAC-SK |
| ON986453 | 2018 | USA | | RAC-SK | North America | RAC-SK |
| ON986454 | 2018 | USA | | RAC-SK | North America | RAC-SK |
| ON986455 | 2018 | USA | | RAC-SK | North America | RAC-SK |
| ON986456 | 2018 | USA | | RAC-SK | North America | RAC-SK |
| ON986457 | 2018 | USA | | RAC-SK | North America | RAC-SK |
| ON986458 | 2019 | USA | | RAC-SK | North America | RAC-SK |
| ON986459 | 2019 | USA | | RAC-SK | North America | RAC-SK |
| ON986460 | 2019 | USA | | RAC-SK | North America | RAC-SK |
| ON986461 | 2019 | USA | | RAC-SK | North America | RAC-SK |
| ON986462 | 2019 | USA | | RAC-SK | North America | RAC-SK |
| ON986463 | 2019 | USA | | RAC-SK | North America | RAC-SK |
| ON986464 | 2019 | USA | | RAC-SK | North America | RAC-SK |
| ON986465 | 2019 | USA | | RAC-SK | North America | RAC-SK |
| ON986466 | 2019 | USA | | RAC-SK | North America | RAC-SK |
| ON986467 | 2019 | USA | | RAC-SK | North America | RAC-SK |
| ON986468 | 2019 | USA | | RAC-SK | North America | RAC-SK |
| ON986469 | 2019 | USA | | RAC-SK | North America | RAC-SK |
| ON986470 | 2019 | USA | | RAC-SK | North America | RAC-SK |
| ON986471 | 2019 | USA | | RAC-SK | North America | RAC-SK |
| ON986472 | 2019 | USA | | RAC-SK | North America | RAC-SK |
| ON986473 | 2020 | USA | | RAC-SK | North America | RAC-SK |
| ON986474 | 2017 | USA | | RAC-SK | North America | RAC-SK |
| ON986475 | 2017 | USA | | RAC-SK | North America | RAC-SK |
| ON986476 | 2017 | USA | | RAC-SK | North America | RAC-SK |
| ON986477 | 2017 | USA | | RAC-SK | North America | RAC-SK |
| ON986478 | 2019 | USA | | RAC-SK | North America | RAC-SK |
| ON986479 | 2019 | USA | | RAC-SK | North America | RAC-SK |
| ON986480 | 2019 | USA | | RAC-SK | North America | RAC-SK |
| ON986481 | 2019 | USA | | RAC-SK | North America | RAC-SK |
| ON986482 | 2018 | USA | | Other | North America | RAC-SK |
| ON986483 | 2018 | USA | | Other | North America | RAC-SK |
| ON986484 | 2019 | USA | | Other | North America | RAC-SK |

Table S2 The CAI, RADI and SiD values of Selective sequences

| **Accession** | | **Virus** | **Host** | **CAI** | **RCDI** | **SiD** |
| --- | --- | --- | --- | --- | --- | --- |
| JQ685896 | Bat-related | | Bat | 0.779357 | 1.072257 | 0.491528 |
| JQ685897 | Bat-related | | Bat | 0.780226 | 1.069399 | 0.491732 |
| JQ685900 | Bat-related | | Bat | 0.783475 | 1.06969 | 0.491545 |
| JQ685903 | Bat-related | | Bat | 0.779379 | 1.07193 | 0.491689 |
| JQ685906 | Bat-related | | Bat | 0.780232 | 1.07137 | 0.491552 |
| JQ685908 | Bat-related | | Bat | 0.77881 | 1.072658 | 0.491521 |
| JQ685910 | Bat-related | | Bat | 0.780946 | 1.06859 | 0.491449 |
| JQ685911 | Bat-related | | Bat | 0.779385 | 1.070929 | 0.491731 |
| JQ685912 | Bat-related | | Bat | 0.779149 | 1.072375 | 0.491698 |
| JQ685913 | Bat-related | | Bat | 0.779373 | 1.070605 | 0.49174 |
| JQ685914 | Bat-related | | Bat | 0.779616 | 1.071175 | 0.491717 |
| JQ685915 | Bat-related | | Bat | 0.781118 | 1.065526 | 0.491716 |
| JQ685916 | Bat-related | | Bat | 0.779334 | 1.065452 | 0.491466 |
| JQ685918 | Bat-related | | Bat | 0.777021 | 1.068476 | 0.491523 |
| JQ685920 | Bat-related | | Bat | 0.779253 | 1.07615 | 0.491725 |
| JQ685922 | Bat-related | | Bat | 0.783635 | 1.071286 | 0.491534 |
| JQ685925 | Bat-related | | Bat | 0.780301 | 1.074515 | 0.491461 |
| JQ685931 | Bat-related | | Bat | 0.78104 | 1.069465 | 0.491459 |
| JQ685934 | Bat-related | | Bat | 0.779626 | 1.072805 | 0.491535 |
| JQ685935 | Bat-related | | Bat | 0.779676 | 1.07088 | 0.49153 |
| JQ685936 | Bat-related | | Bat | 0.780286 | 1.071757 | 0.49172 |
| JQ685940 | Bat-related | | Bat | 0.779264 | 1.071269 | 0.49151 |
| JQ685942 | Bat-related | | Bat | 0.781413 | 1.07628 | 0.491512 |
| JQ685945 | Bat-related | | Bat | 0.780079 | 1.069175 | 0.491516 |
| JQ685946 | Bat-related | | Bat | 0.781819 | 1.071832 | 0.491553 |
| JQ685949 | Bat-related | | Bat | 0.779728 | 1.071 | 0.491721 |
| JQ685953 | Bat-related | | Bat | 0.77628 | 1.07854 | 0.491385 |
| JQ685957 | Bat-related | | Bat | 0.777773 | 1.070411 | 0.491708 |
| JQ685959 | Bat-related | | Bat | 0.779065 | 1.071395 | 0.491517 |
| JQ685963 | Bat-related | | Bat | 0.781737 | 1.081126 | 0.491522 |
| JQ685966 | Bat-related | | Bat | 0.779676 | 1.07088 | 0.491471 |
| JQ685971 | Bat-related | | Bat | 0.779176 | 1.079729 | 0.491479 |
| JQ685974 | Bat-related | | Bat | 0.77878 | 1.070944 | 0.491517 |
| KM594024 | Bat-related | | Bat | 0.774117 | 1.06401 | 0.49171 |
| KM594025 | Bat-related | | Bat | 0.779682 | 1.058936 | 0.491402 |
| KM594031 | Bat-related | | Bat | 0.781185 | 1.078357 | 0.491392 |
| KM594032 | Bat-related | | Bat | 0.775736 | 1.066496 | 0.491472 |
| KM594033 | Bat-related | | Bat | 0.782166 | 1.083348 | 0.491627 |
| KM594036 | Bat-related | | Bat | 0.782038 | 1.087084 | 0.491711 |
| KM594038 | Bat-related | | Bat | 0.779559 | 1.076569 | 0.491717 |
| KM594040 | Bat-related | | Bat | 0.778041 | 1.076285 | 0.491714 |
| KM594042 | Bat-related | | Bat | 0.778583 | 1.075658 | 0.491485 |
| KM594043 | Bat-related | | Bat | 0.777376 | 1.078537 | 0.49144 |
| KX148100 | Bat-related | | Bat | 0.77921 | 1.076064 | 0.491541 |
| KX148109 | Bat-related | | Bat | 0.778692 | 1.07772 | 0.491548 |
| KX148268 | Bat-related | | Bat | 0.780212 | 1.0785 | 0.491713 |
| MG458304 | Bat-related | | Bat | 0.779715 | 1.069591 | 0.491719 |
| MK920923 | Bat-related | | Bat | 0.777364 | 1.068514 | 0.491525 |
| OM971001 | Bat-related | | Bat | 0.782602 | 1.080257 | 0.49155 |
| OM971002 | Bat-related | | Bat | 0.782153 | 1.08693 | 0.491744 |
| EF564174 | Dog-related | | Bat | 0.77633 | 1.073455 | 0.491721 |
| EU886631 | Dog-related | | Bat | 0.782701 | 1.077955 | 0.49149 |
| FJ712193 | Dog-related | | Bat | 0.780896 | 1.072996 | 0.491532 |
| FJ866835 | Dog-related | | Bat | 0.781934 | 1.075841 | 0.49153 |
| JX473840 | Dog-related | | Bat | 0.777694 | 1.075637 | 0.491716 |
| KR534217 | Dog-related | | Bat | 0.778812 | 1.077025 | 0.491565 |
| KR906753 | Dog-related | | Bat | 0.775467 | 1.075961 | 0.491542 |
| KR906757 | Dog-related | | Bat | 0.778877 | 1.079365 | 0.491708 |
| KR906763 | Dog-related | | Bat | 0.778154 | 1.073089 | 0.491723 |
| KX036363 | Dog-related | | Bat | 0.780943 | 1.075546 | 0.491718 |
| KX148114 | Dog-related | | Bat | 0.779663 | 1.072013 | 0.491707 |
| KX148211 | Dog-related | | Bat | 0.778884 | 1.083721 | 0.491513 |
| KX148227 | Dog-related | | Bat | 0.773736 | 1.0686 | 0.491451 |
| KX148260 | Dog-related | | Bat | 0.774236 | 1.066942 | 0.491594 |
| KY210225 | Dog-related | | Bat | 0.777182 | 1.076012 | 0.491542 |
| KY210253 | Dog-related | | Bat | 0.778162 | 1.077175 | 0.491702 |
| KY210290 | Dog-related | | Bat | 0.77793 | 1.076837 | 0.491721 |
| KY210303 | Dog-related | | Bat | 0.777806 | 1.077156 | 0.49171 |
| KY210311 | Dog-related | | Bat | 0.776101 | 1.077216 | 0.491451 |
| LC717423 | Dog-related | | Bat | 0.774197 | 1.065658 | 0.491382 |
| LM645024 | Dog-related | | Bat | 0.77936 | 1.074823 | 0.491605 |
| LM645043 | Dog-related | | Bat | 0.780135 | 1.078037 | 0.491715 |
| MF197741 | Dog-related | | Bat | 0.77665 | 1.063124 | 0.491552 |
| MH514972 | Dog-related | | Bat | 0.771811 | 1.05877 | 0.491537 |
| MK540913 | Dog-related | | Bat | 0.781084 | 1.074061 | 0.491713 |
| MK540920 | Dog-related | | Bat | 0.779536 | 1.074549 | 0.491719 |
| MK540960 | Dog-related | | Bat | 0.781786 | 1.076353 | 0.491701 |
| MK540990 | Dog-related | | Bat | 0.780676 | 1.075372 | 0.491542 |
| MK541000 | Dog-related | | Bat | 0.781191 | 1.074216 | 0.491705 |
| MK598383 | Dog-related | | Bat | 0.777232 | 1.073727 | 0.491543 |
| MK760690 | Dog-related | | Bat | 0.778788 | 1.077255 | 0.491485 |
| MN175989 | Dog-related | | Bat | 0.780594 | 1.073167 | 0.49155 |
| MN233910 | Dog-related | | Bat | 0.779126 | 1.077686 | 0.491543 |
| MN233914 | Dog-related | | Bat | 0.779958 | 1.076835 | 0.491707 |
| MN233919 | Dog-related | | Bat | 0.778666 | 1.076524 | 0.491705 |
| MN233936 | Dog-related | | Bat | 0.779424 | 1.076454 | 0.491711 |
| MN233942 | Dog-related | | Bat | 0.778536 | 1.075425 | 0.491712 |
| MN233949 | Dog-related | | Bat | 0.779358 | 1.077038 | 0.491675 |
| MN233962 | Dog-related | | Bat | 0.779922 | 1.077229 | 0.491709 |
| MN233974 | Dog-related | | Bat | 0.779223 | 1.07753 | 0.491521 |
| MN233999 | Dog-related | | Bat | 0.778698 | 1.075625 | 0.49172 |
| MN234029 | Dog-related | | Bat | 0.779587 | 1.077662 | 0.491699 |
| MN234032 | Dog-related | | Bat | 0.779141 | 1.077623 | 0.491723 |
| MN234033 | Dog-related | | Bat | 0.778862 | 1.07549 | 0.491529 |
| MN234053 | Dog-related | | Bat | 0.778825 | 1.073937 | 0.491514 |
| MN726812 | Dog-related | | Bat | 0.776585 | 1.078426 | 0.491517 |
| MN726815 | Dog-related | | Bat | 0.778012 | 1.070903 | 0.491543 |
| MT454632 | Dog-related | | Bat | 0.778965 | 1.077225 | 0.491476 |
| MT454649 | Dog-related | | Bat | 0.779125 | 1.078547 | 0.491488 |
| OM542191 | Dog-related | | Bat | 0.776416 | 1.069298 | 0.491547 |
| JQ685929 | RAC&SK-related | | Bat | 0.784386 | 1.067483 | 0.491479 |
| KY026418 | RAC&SK-related | | Bat | 0.780676 | 1.072163 | 0.491501 |
| KY026441 | RAC&SK-related | | Bat | 0.78066 | 1.071293 | 0.491608 |
| KY026446 | RAC&SK-related | | Bat | 0.780334 | 1.072472 | 0.491525 |
| KY026456 | RAC&SK-related | | Bat | 0.781222 | 1.073442 | 0.49172 |
| KY026472 | RAC&SK-related | | Bat | 0.780733 | 1.071615 | 0.491719 |
| KY026480 | RAC&SK-related | | Bat | 0.781526 | 1.07114 | 0.491432 |
| MF143205 | RAC&SK-related | | Bat | 0.782784 | 1.076076 | 0.49153 |
| MF143237 | RAC&SK-related | | Bat | 0.784714 | 1.07529 | 0.491548 |
| MF143262 | RAC&SK-related | | Bat | 0.780609 | 1.072522 | 0.491526 |
| MF143270 | RAC&SK-related | | Bat | 0.781129 | 1.07264 | 0.49165 |
| MF143320 | RAC&SK-related | | Bat | 0.780994 | 1.073458 | 0.491714 |
| MF143327 | RAC&SK-related | | Bat | 0.78132 | 1.072315 | 0.491544 |
| MF143334 | RAC&SK-related | | Bat | 0.780923 | 1.073434 | 0.491714 |
| MF143338 | RAC&SK-related | | Bat | 0.78058 | 1.071099 | 0.491548 |
| MF143350 | RAC&SK-related | | Bat | 0.780674 | 1.072927 | 0.491519 |
| MF143362 | RAC&SK-related | | Bat | 0.780311 | 1.072592 | 0.491536 |
| MF143367 | RAC&SK-related | | Bat | 0.780797 | 1.072333 | 0.491717 |
| MF143382 | RAC&SK-related | | Bat | 0.780441 | 1.07276 | 0.491703 |
| MF143387 | RAC&SK-related | | Bat | 0.78109 | 1.071602 | 0.491721 |
| MG562535 | RAC&SK-related | | Bat | 0.780984 | 1.074146 | 0.491535 |
| MG562552 | RAC&SK-related | | Bat | 0.780492 | 1.070482 | 0.491497 |
| MG562555 | RAC&SK-related | | Bat | 0.780943 | 1.070891 | 0.491374 |
| MG562560 | RAC&SK-related | | Bat | 0.781013 | 1.070918 | 0.491706 |
| MG562564 | RAC&SK-related | | Bat | 0.781031 | 1.072459 | 0.491709 |
| MG562567 | RAC&SK-related | | Bat | 0.780199 | 1.075194 | 0.491547 |
| MG562572 | RAC&SK-related | | Bat | 0.780387 | 1.072505 | 0.491711 |
| MG562586 | RAC&SK-related | | Bat | 0.780916 | 1.072273 | 0.491518 |
| MG562605 | RAC&SK-related | | Bat | 0.780747 | 1.071724 | 0.491454 |
| MK540658 | RAC&SK-related | | Bat | 0.78103 | 1.073901 | 0.491517 |
| MK540668 | RAC&SK-related | | Bat | 0.780898 | 1.072424 | 0.491409 |
| MK540669 | RAC&SK-related | | Bat | 0.781187 | 1.073678 | 0.491508 |
| MK540677 | RAC&SK-related | | Bat | 0.780726 | 1.072674 | 0.4917 |
| MK540702 | RAC&SK-related | | Bat | 0.780288 | 1.073495 | 0.491711 |
| MK540720 | RAC&SK-related | | Bat | 0.781457 | 1.071816 | 0.491712 |
| MK540723 | RAC&SK-related | | Bat | 0.78008 | 1.073516 | 0.491629 |
| MK540745 | RAC&SK-related | | Bat | 0.780261 | 1.07299 | 0.491716 |
| MK540747 | RAC&SK-related | | Bat | 0.781994 | 1.073853 | 0.491708 |
| MK540749 | RAC&SK-related | | Bat | 0.781199 | 1.072935 | 0.491637 |
| MK540761 | RAC&SK-related | | Bat | 0.779658 | 1.074249 | 0.491715 |
| MK540796 | RAC&SK-related | | Bat | 0.781095 | 1.071088 | 0.49161 |
| MK540799 | RAC&SK-related | | Bat | 0.780646 | 1.071814 | 0.491612 |
| MK540826 | RAC&SK-related | | Bat | 0.779723 | 1.071057 | 0.491446 |
| MK540853 | RAC&SK-related | | Bat | 0.780035 | 1.071717 | 0.491723 |
| MN418144 | RAC&SK-related | | Bat | 0.78176 | 1.074838 | 0.491573 |
| MN418168 | RAC&SK-related | | Bat | 0.781666 | 1.072713 | 0.491711 |
| MN418172 | RAC&SK-related | | Bat | 0.780505 | 1.071436 | 0.491522 |
| ON986457 | RAC&SK-related | | Bat | 0.78307 | 1.07568 | 0.491717 |
| ON986465 | RAC&SK-related | | Bat | 0.782284 | 1.074308 | 0.491714 |
| ON986474 | RAC&SK-related | | Bat | 0.782293 | 1.070753 | 0.491515 |
| JQ685896 | Bat-related | | Canis | 0.778876 | 1.09113 | 0.491582 |
| JQ685897 | Bat-related | | Canis | 0.779773 | 1.086452 | 0.49177 |
| JQ685900 | Bat-related | | Canis | 0.783088 | 1.089297 | 0.491581 |
| JQ685903 | Bat-related | | Canis | 0.778991 | 1.091582 | 0.491754 |
| JQ685906 | Bat-related | | Canis | 0.779773 | 1.088414 | 0.4916 |
| JQ685908 | Bat-related | | Canis | 0.778318 | 1.091449 | 0.491543 |
| JQ685910 | Bat-related | | Canis | 0.780657 | 1.089111 | 0.491474 |
| JQ685911 | Bat-related | | Canis | 0.778903 | 1.088053 | 0.491796 |
| JQ685912 | Bat-related | | Canis | 0.77866 | 1.091172 | 0.491743 |
| JQ685913 | Bat-related | | Canis | 0.778876 | 1.088539 | 0.491793 |
| JQ685914 | Bat-related | | Canis | 0.779173 | 1.090502 | 0.49178 |
| JQ685915 | Bat-related | | Canis | 0.780335 | 1.082075 | 0.491779 |
| JQ685916 | Bat-related | | Canis | 0.778501 | 1.079491 | 0.491497 |
| JQ685918 | Bat-related | | Canis | 0.776437 | 1.08755 | 0.491571 |
| JQ685920 | Bat-related | | Canis | 0.779 | 1.0958 | 0.49179 |
| JQ685922 | Bat-related | | Canis | 0.783426 | 1.091417 | 0.491588 |
| JQ685925 | Bat-related | | Canis | 0.780043 | 1.094113 | 0.491493 |
| JQ685931 | Bat-related | | Canis | 0.780577 | 1.088433 | 0.491489 |
| JQ685934 | Bat-related | | Canis | 0.779137 | 1.09184 | 0.49156 |
| JQ685935 | Bat-related | | Canis | 0.779156 | 1.08799 | 0.491585 |
| JQ685936 | Bat-related | | Canis | 0.779682 | 1.086475 | 0.491764 |
| JQ685940 | Bat-related | | Canis | 0.778733 | 1.088434 | 0.49156 |
| JQ685942 | Bat-related | | Canis | 0.780936 | 1.093512 | 0.491569 |
| JQ685945 | Bat-related | | Canis | 0.779626 | 1.085961 | 0.491566 |
| JQ685946 | Bat-related | | Canis | 0.781367 | 1.090257 | 0.491607 |
| JQ685949 | Bat-related | | Canis | 0.779208 | 1.088142 | 0.491785 |
| JQ685953 | Bat-related | | Canis | 0.775538 | 1.094095 | 0.491431 |
| JQ685957 | Bat-related | | Canis | 0.777412 | 1.089132 | 0.491771 |
| JQ685959 | Bat-related | | Canis | 0.778575 | 1.088459 | 0.491569 |
| JQ685963 | Bat-related | | Canis | 0.781076 | 1.098121 | 0.491572 |
| JQ685966 | Bat-related | | Canis | 0.779156 | 1.08799 | 0.491528 |
| JQ685971 | Bat-related | | Canis | 0.778636 | 1.096159 | 0.491527 |
| JQ685974 | Bat-related | | Canis | 0.778338 | 1.088672 | 0.491567 |
| KM594024 | Bat-related | | Canis | 0.774062 | 1.079018 | 0.491748 |
| KM594025 | Bat-related | | Canis | 0.779261 | 1.072957 | 0.491445 |
| KM594031 | Bat-related | | Canis | 0.780726 | 1.097997 | 0.491422 |
| KM594032 | Bat-related | | Canis | 0.775316 | 1.08646 | 0.491536 |
| KM594033 | Bat-related | | Canis | 0.781624 | 1.101817 | 0.491657 |
| KM594036 | Bat-related | | Canis | 0.781476 | 1.107177 | 0.491776 |
| KM594038 | Bat-related | | Canis | 0.779077 | 1.091523 | 0.491783 |
| KM594040 | Bat-related | | Canis | 0.777541 | 1.090815 | 0.491779 |
| KM594042 | Bat-related | | Canis | 0.778089 | 1.089715 | 0.491519 |
| KM594043 | Bat-related | | Canis | 0.776837 | 1.094477 | 0.491467 |
| KX148100 | Bat-related | | Canis | 0.778641 | 1.090399 | 0.49157 |
| KX148109 | Bat-related | | Canis | 0.77818 | 1.094382 | 0.491572 |
| KX148268 | Bat-related | | Canis | 0.779609 | 1.092859 | 0.491775 |
| MG458304 | Bat-related | | Canis | 0.779251 | 1.086437 | 0.491755 |
| MK920923 | Bat-related | | Canis | 0.776802 | 1.085081 | 0.491579 |
| OM971001 | Bat-related | | Canis | 0.781954 | 1.09817 | 0.491575 |
| OM971002 | Bat-related | | Canis | 0.781429 | 1.105784 | 0.491787 |
| EF564174 | Dog-related | | Canis | 0.776426 | 1.093202 | 0.491764 |
| EU886631 | Dog-related | | Canis | 0.782436 | 1.093226 | 0.491535 |
| FJ712193 | Dog-related | | Canis | 0.780898 | 1.090071 | 0.491555 |
| FJ866835 | Dog-related | | Canis | 0.78167 | 1.092489 | 0.491586 |
| JX473840 | Dog-related | | Canis | 0.777501 | 1.093473 | 0.491781 |
| KR534217 | Dog-related | | Canis | 0.77859 | 1.094478 | 0.491604 |
| KR906753 | Dog-related | | Canis | 0.77524 | 1.093269 | 0.491567 |
| KR906757 | Dog-related | | Canis | 0.778639 | 1.095596 | 0.491743 |
| KR906763 | Dog-related | | Canis | 0.777981 | 1.09055 | 0.491788 |
| KX036363 | Dog-related | | Canis | 0.780534 | 1.089491 | 0.491764 |
| KX148114 | Dog-related | | Canis | 0.779443 | 1.087003 | 0.491774 |
| KX148211 | Dog-related | | Canis | 0.778514 | 1.101423 | 0.491562 |
| KX148227 | Dog-related | | Canis | 0.773706 | 1.085343 | 0.491477 |
| KX148260 | Dog-related | | Canis | 0.773902 | 1.085044 | 0.491636 |
| KY210225 | Dog-related | | Canis | 0.776908 | 1.093546 | 0.49159 |
| KY210253 | Dog-related | | Canis | 0.778013 | 1.095104 | 0.491766 |
| KY210290 | Dog-related | | Canis | 0.777674 | 1.094095 | 0.491783 |
| KY210303 | Dog-related | | Canis | 0.777603 | 1.095303 | 0.491774 |
| KY210311 | Dog-related | | Canis | 0.775892 | 1.093643 | 0.491487 |
| LC717423 | Dog-related | | Canis | 0.773928 | 1.081786 | 0.491421 |
| LM645024 | Dog-related | | Canis | 0.778992 | 1.088712 | 0.491639 |
| LM645043 | Dog-related | | Canis | 0.779933 | 1.092428 | 0.49178 |
| MF197741 | Dog-related | | Canis | 0.776584 | 1.07795 | 0.491606 |
| MH514972 | Dog-related | | Canis | 0.771319 | 1.076175 | 0.491594 |
| MK540913 | Dog-related | | Canis | 0.780627 | 1.08945 | 0.491778 |
| MK540920 | Dog-related | | Canis | 0.779009 | 1.091323 | 0.491782 |
| MK540960 | Dog-related | | Canis | 0.781341 | 1.092071 | 0.491768 |
| MK540990 | Dog-related | | Canis | 0.780185 | 1.091263 | 0.491566 |
| MK541000 | Dog-related | | Canis | 0.780756 | 1.090166 | 0.491772 |
| MK598383 | Dog-related | | Canis | 0.776847 | 1.08896 | 0.491569 |
| MK760690 | Dog-related | | Canis | 0.778608 | 1.093909 | 0.491526 |
| MN175989 | Dog-related | | Canis | 0.780396 | 1.089648 | 0.491618 |
| MN233910 | Dog-related | | Canis | 0.778757 | 1.091664 | 0.491567 |
| MN233914 | Dog-related | | Canis | 0.779549 | 1.091012 | 0.491769 |
| MN233919 | Dog-related | | Canis | 0.778228 | 1.090584 | 0.491772 |
| MN233936 | Dog-related | | Canis | 0.779062 | 1.090339 | 0.491755 |
| MN233942 | Dog-related | | Canis | 0.778176 | 1.089551 | 0.491776 |
| MN233949 | Dog-related | | Canis | 0.779 | 1.091295 | 0.491719 |
| MN233962 | Dog-related | | Canis | 0.779491 | 1.091098 | 0.491775 |
| MN233974 | Dog-related | | Canis | 0.778814 | 1.09154 | 0.491578 |
| MN233999 | Dog-related | | Canis | 0.778318 | 1.089271 | 0.491781 |
| MN234029 | Dog-related | | Canis | 0.77915 | 1.091973 | 0.491749 |
| MN234032 | Dog-related | | Canis | 0.778769 | 1.091829 | 0.491787 |
| MN234033 | Dog-related | | Canis | 0.778467 | 1.089689 | 0.491553 |
| MN234053 | Dog-related | | Canis | 0.778491 | 1.088046 | 0.491558 |
| MN726812 | Dog-related | | Canis | 0.776386 | 1.095793 | 0.491567 |
| MN726815 | Dog-related | | Canis | 0.777766 | 1.089464 | 0.491566 |
| MT454632 | Dog-related | | Canis | 0.778658 | 1.0915 | 0.491508 |
| MT454649 | Dog-related | | Canis | 0.779045 | 1.093461 | 0.491525 |
| OM542191 | Dog-related | | Canis | 0.776105 | 1.084902 | 0.491602 |
| JQ685929 | RAC&SK-related | | Canis | 0.784092 | 1.083216 | 0.49153 |
| KY026418 | RAC&SK-related | | Canis | 0.780141 | 1.086568 | 0.491557 |
| KY026441 | RAC&SK-related | | Canis | 0.780149 | 1.085778 | 0.491638 |
| KY026446 | RAC&SK-related | | Canis | 0.779852 | 1.086615 | 0.491581 |
| KY026456 | RAC&SK-related | | Canis | 0.780706 | 1.087799 | 0.491782 |
| KY026472 | RAC&SK-related | | Canis | 0.780176 | 1.085937 | 0.491782 |
| KY026480 | RAC&SK-related | | Canis | 0.780956 | 1.084801 | 0.491479 |
| MF143205 | RAC&SK-related | | Canis | 0.782282 | 1.089179 | 0.491584 |
| MF143237 | RAC&SK-related | | Canis | 0.784211 | 1.089159 | 0.491572 |
| MF143262 | RAC&SK-related | | Canis | 0.780029 | 1.085444 | 0.491576 |
| MF143270 | RAC&SK-related | | Canis | 0.780546 | 1.085506 | 0.491688 |
| MF143320 | RAC&SK-related | | Canis | 0.780437 | 1.087574 | 0.491777 |
| MF143327 | RAC&SK-related | | Canis | 0.780721 | 1.085837 | 0.49157 |
| MF143334 | RAC&SK-related | | Canis | 0.780285 | 1.086886 | 0.491779 |
| MF143338 | RAC&SK-related | | Canis | 0.780002 | 1.08483 | 0.491572 |
| MF143350 | RAC&SK-related | | Canis | 0.780076 | 1.085747 | 0.491569 |
| MF143362 | RAC&SK-related | | Canis | 0.779727 | 1.085206 | 0.491561 |
| MF143367 | RAC&SK-related | | Canis | 0.780228 | 1.085274 | 0.49178 |
| MF143382 | RAC&SK-related | | Canis | 0.779848 | 1.085692 | 0.491768 |
| MF143387 | RAC&SK-related | | Canis | 0.78052 | 1.084785 | 0.491786 |
| MG562535 | RAC&SK-related | | Canis | 0.780387 | 1.087448 | 0.49158 |
| MG562552 | RAC&SK-related | | Canis | 0.77988 | 1.083376 | 0.491557 |
| MG562555 | RAC&SK-related | | Canis | 0.780328 | 1.083921 | 0.491421 |
| MG562560 | RAC&SK-related | | Canis | 0.780444 | 1.083904 | 0.491768 |
| MG562564 | RAC&SK-related | | Canis | 0.780455 | 1.08518 | 0.491771 |
| MG562567 | RAC&SK-related | | Canis | 0.779586 | 1.088549 | 0.491571 |
| MG562572 | RAC&SK-related | | Canis | 0.779782 | 1.086095 | 0.491777 |
| MG562586 | RAC&SK-related | | Canis | 0.78033 | 1.085361 | 0.491556 |
| MG562605 | RAC&SK-related | | Canis | 0.780244 | 1.084739 | 0.491481 |
| MK540658 | RAC&SK-related | | Canis | 0.780503 | 1.087969 | 0.491568 |
| MK540668 | RAC&SK-related | | Canis | 0.780304 | 1.085709 | 0.491464 |
| MK540669 | RAC&SK-related | | Canis | 0.78068 | 1.087817 | 0.491558 |
| MK540677 | RAC&SK-related | | Canis | 0.780189 | 1.086719 | 0.491765 |
| MK540702 | RAC&SK-related | | Canis | 0.779664 | 1.086851 | 0.491776 |
| MK540720 | RAC&SK-related | | Canis | 0.780927 | 1.084469 | 0.491776 |
| MK540723 | RAC&SK-related | | Canis | 0.77947 | 1.086887 | 0.491668 |
| MK540745 | RAC&SK-related | | Canis | 0.77964 | 1.086482 | 0.491782 |
| MK540747 | RAC&SK-related | | Canis | 0.781411 | 1.086555 | 0.491775 |
| MK540749 | RAC&SK-related | | Canis | 0.780618 | 1.086379 | 0.491677 |
| MK540761 | RAC&SK-related | | Canis | 0.779025 | 1.088005 | 0.491779 |
| MK540796 | RAC&SK-related | | Canis | 0.780597 | 1.085089 | 0.491667 |
| MK540799 | RAC&SK-related | | Canis | 0.780048 | 1.084463 | 0.491673 |
| MK540826 | RAC&SK-related | | Canis | 0.779172 | 1.084588 | 0.491488 |
| MK540853 | RAC&SK-related | | Canis | 0.779482 | 1.08513 | 0.49177 |
| MN418144 | RAC&SK-related | | Canis | 0.781117 | 1.088429 | 0.491633 |
| MN418168 | RAC&SK-related | | Canis | 0.781076 | 1.085984 | 0.491777 |
| MN418172 | RAC&SK-related | | Canis | 0.77993 | 1.083905 | 0.491572 |
| ON986457 | RAC&SK-related | | Canis | 0.782581 | 1.089136 | 0.491783 |
| ON986465 | RAC&SK-related | | Canis | 0.781807 | 1.088043 | 0.491777 |
| ON986474 | RAC&SK-related | | Canis | 0.781848 | 1.085348 | 0.491573 |
| JQ685896 | Bat-related | | Human | 0.773902 | 1.070392 | 0.491477 |
| JQ685897 | Bat-related | | Human | 0.774786 | 1.067875 | 0.491682 |
| JQ685900 | Bat-related | | Human | 0.777978 | 1.068164 | 0.491494 |
| JQ685903 | Bat-related | | Human | 0.773929 | 1.070626 | 0.491634 |
| JQ685906 | Bat-related | | Human | 0.774751 | 1.069675 | 0.491502 |
| JQ685908 | Bat-related | | Human | 0.773358 | 1.070965 | 0.491464 |
| JQ685910 | Bat-related | | Human | 0.77548 | 1.06669 | 0.491396 |
| JQ685911 | Bat-related | | Human | 0.773937 | 1.069353 | 0.491679 |
| JQ685912 | Bat-related | | Human | 0.773693 | 1.07054 | 0.491644 |
| JQ685913 | Bat-related | | Human | 0.773922 | 1.069044 | 0.491691 |
| JQ685914 | Bat-related | | Human | 0.774183 | 1.070069 | 0.491664 |
| JQ685915 | Bat-related | | Human | 0.775827 | 1.063687 | 0.491663 |
| JQ685916 | Bat-related | | Human | 0.774049 | 1.063233 | 0.491408 |
| JQ685918 | Bat-related | | Human | 0.771706 | 1.066017 | 0.491474 |
| JQ685920 | Bat-related | | Human | 0.773928 | 1.074739 | 0.491672 |
| JQ685922 | Bat-related | | Human | 0.77805 | 1.068497 | 0.491484 |
| JQ685925 | Bat-related | | Human | 0.774875 | 1.072882 | 0.491403 |
| JQ685931 | Bat-related | | Human | 0.775589 | 1.068021 | 0.491406 |
| JQ685934 | Bat-related | | Human | 0.774172 | 1.070978 | 0.491477 |
| JQ685935 | Bat-related | | Human | 0.774227 | 1.069186 | 0.491479 |
| JQ685936 | Bat-related | | Human | 0.774953 | 1.069373 | 0.49167 |
| JQ685940 | Bat-related | | Human | 0.773829 | 1.069806 | 0.491459 |
| JQ685942 | Bat-related | | Human | 0.775943 | 1.074507 | 0.491462 |
| JQ685945 | Bat-related | | Human | 0.774656 | 1.067744 | 0.491465 |
| JQ685946 | Bat-related | | Human | 0.77631 | 1.06962 | 0.491496 |
| JQ685949 | Bat-related | | Human | 0.774279 | 1.069341 | 0.491668 |
| JQ685953 | Bat-related | | Human | 0.770908 | 1.076934 | 0.491333 |
| JQ685957 | Bat-related | | Human | 0.772396 | 1.068303 | 0.491655 |
| JQ685959 | Bat-related | | Human | 0.773614 | 1.069986 | 0.491467 |
| JQ685963 | Bat-related | | Human | 0.776405 | 1.078408 | 0.491472 |
| JQ685966 | Bat-related | | Human | 0.774227 | 1.069186 | 0.491418 |
| JQ685971 | Bat-related | | Human | 0.773875 | 1.077774 | 0.491431 |
| JQ685974 | Bat-related | | Human | 0.773313 | 1.06938 | 0.491466 |
| KM594024 | Bat-related | | Human | 0.768656 | 1.064402 | 0.491659 |
| KM594025 | Bat-related | | Human | 0.774265 | 1.058032 | 0.491357 |
| KM594031 | Bat-related | | Human | 0.775829 | 1.076676 | 0.49134 |
| KM594032 | Bat-related | | Human | 0.770498 | 1.06561 | 0.491419 |
| KM594033 | Bat-related | | Human | 0.776727 | 1.081105 | 0.49157 |
| KM594036 | Bat-related | | Human | 0.776649 | 1.084658 | 0.491658 |
| KM594038 | Bat-related | | Human | 0.774186 | 1.074947 | 0.491664 |
| KM594040 | Bat-related | | Human | 0.772719 | 1.074289 | 0.49166 |
| KM594042 | Bat-related | | Human | 0.773256 | 1.073766 | 0.491432 |
| KM594043 | Bat-related | | Human | 0.772026 | 1.075694 | 0.491385 |
| KX148100 | Bat-related | | Human | 0.773884 | 1.072726 | 0.491487 |
| KX148109 | Bat-related | | Human | 0.773353 | 1.075616 | 0.491493 |
| KX148268 | Bat-related | | Human | 0.774888 | 1.074989 | 0.49166 |
| MG458304 | Bat-related | | Human | 0.774288 | 1.067957 | 0.491666 |
| MK920923 | Bat-related | | Human | 0.771944 | 1.069303 | 0.491473 |
| OM971001 | Bat-related | | Human | 0.777278 | 1.077015 | 0.491496 |
| OM971002 | Bat-related | | Human | 0.776858 | 1.083269 | 0.491698 |
| EF564174 | Dog-related | | Human | 0.770829 | 1.073458 | 0.491671 |
| EU886631 | Dog-related | | Human | 0.77719 | 1.074785 | 0.491447 |
| FJ712193 | Dog-related | | Human | 0.775545 | 1.071264 | 0.491476 |
| FJ866835 | Dog-related | | Human | 0.776646 | 1.074028 | 0.491476 |
| JX473840 | Dog-related | | Human | 0.772226 | 1.073928 | 0.491662 |
| KR534217 | Dog-related | | Human | 0.77341 | 1.075746 | 0.491515 |
| KR906753 | Dog-related | | Human | 0.77011 | 1.075919 | 0.491486 |
| KR906757 | Dog-related | | Human | 0.773457 | 1.077568 | 0.491659 |
| KR906763 | Dog-related | | Human | 0.772716 | 1.072496 | 0.491671 |
| KX036363 | Dog-related | | Human | 0.775528 | 1.073353 | 0.491668 |
| KX148114 | Dog-related | | Human | 0.77433 | 1.07007 | 0.491654 |
| KX148211 | Dog-related | | Human | 0.773507 | 1.081846 | 0.491463 |
| KX148227 | Dog-related | | Human | 0.768285 | 1.068317 | 0.491397 |
| KX148260 | Dog-related | | Human | 0.768893 | 1.067425 | 0.491542 |
| KY210225 | Dog-related | | Human | 0.77183 | 1.07514 | 0.491485 |
| KY210253 | Dog-related | | Human | 0.77274 | 1.076347 | 0.491646 |
| KY210290 | Dog-related | | Human | 0.772563 | 1.075941 | 0.491667 |
| KY210303 | Dog-related | | Human | 0.772415 | 1.076339 | 0.491656 |
| KY210311 | Dog-related | | Human | 0.770704 | 1.076388 | 0.491396 |
| LC717423 | Dog-related | | Human | 0.768828 | 1.065033 | 0.491325 |
| LM645024 | Dog-related | | Human | 0.77397 | 1.072729 | 0.491546 |
| LM645043 | Dog-related | | Human | 0.774662 | 1.076045 | 0.491662 |
| MF197741 | Dog-related | | Human | 0.771182 | 1.062021 | 0.491506 |
| MH514972 | Dog-related | | Human | 0.7664 | 1.057454 | 0.491486 |
| MK540913 | Dog-related | | Human | 0.775685 | 1.072862 | 0.49166 |
| MK540920 | Dog-related | | Human | 0.774154 | 1.073483 | 0.491667 |
| MK540960 | Dog-related | | Human | 0.776377 | 1.075158 | 0.491647 |
| MK540990 | Dog-related | | Human | 0.775274 | 1.074342 | 0.491486 |
| MK541000 | Dog-related | | Human | 0.77581 | 1.072888 | 0.491652 |
| MK598383 | Dog-related | | Human | 0.771736 | 1.071203 | 0.491486 |
| MK760690 | Dog-related | | Human | 0.77333 | 1.075513 | 0.491432 |
| MN175989 | Dog-related | | Human | 0.775294 | 1.071825 | 0.491502 |
| MN233910 | Dog-related | | Human | 0.773699 | 1.076154 | 0.491487 |
| MN233914 | Dog-related | | Human | 0.774515 | 1.074795 | 0.491653 |
| MN233919 | Dog-related | | Human | 0.77327 | 1.075073 | 0.491652 |
| MN233936 | Dog-related | | Human | 0.773992 | 1.075047 | 0.491659 |
| MN233942 | Dog-related | | Human | 0.773118 | 1.074199 | 0.491659 |
| MN233949 | Dog-related | | Human | 0.773915 | 1.075369 | 0.491627 |
| MN233962 | Dog-related | | Human | 0.774504 | 1.075884 | 0.491657 |
| MN233974 | Dog-related | | Human | 0.773801 | 1.07623 | 0.491466 |
| MN233999 | Dog-related | | Human | 0.773279 | 1.074498 | 0.491667 |
| MN234029 | Dog-related | | Human | 0.774172 | 1.076416 | 0.491645 |
| MN234032 | Dog-related | | Human | 0.773705 | 1.076735 | 0.49167 |
| MN234033 | Dog-related | | Human | 0.773428 | 1.073898 | 0.491472 |
| MN234053 | Dog-related | | Human | 0.77341 | 1.071734 | 0.491461 |
| MN726812 | Dog-related | | Human | 0.771164 | 1.078448 | 0.491466 |
| MN726815 | Dog-related | | Human | 0.772584 | 1.069994 | 0.491487 |
| MT454632 | Dog-related | | Human | 0.77352 | 1.076024 | 0.491423 |
| MT454649 | Dog-related | | Human | 0.77362 | 1.07749 | 0.491432 |
| OM542191 | Dog-related | | Human | 0.770953 | 1.067241 | 0.491497 |
| JQ685929 | RAC&SK-related | | Human | 0.778875 | 1.064713 | 0.491429 |
| KY026418 | RAC&SK-related | | Human | 0.775412 | 1.069967 | 0.491448 |
| KY026441 | RAC&SK-related | | Human | 0.775387 | 1.069224 | 0.491552 |
| KY026446 | RAC&SK-related | | Human | 0.775066 | 1.070427 | 0.491473 |
| KY026456 | RAC&SK-related | | Human | 0.775966 | 1.071319 | 0.491668 |
| KY026472 | RAC&SK-related | | Human | 0.775478 | 1.06964 | 0.491666 |
| KY026480 | RAC&SK-related | | Human | 0.776261 | 1.068954 | 0.491373 |
| MF143205 | RAC&SK-related | | Human | 0.777558 | 1.073396 | 0.491479 |
| MF143237 | RAC&SK-related | | Human | 0.779442 | 1.072358 | 0.491492 |
| MF143262 | RAC&SK-related | | Human | 0.775354 | 1.070443 | 0.491475 |
| MF143270 | RAC&SK-related | | Human | 0.775868 | 1.070545 | 0.491596 |
| MF143320 | RAC&SK-related | | Human | 0.77577 | 1.071091 | 0.491661 |
| MF143327 | RAC&SK-related | | Human | 0.776078 | 1.069691 | 0.491488 |
| MF143334 | RAC&SK-related | | Human | 0.775677 | 1.071499 | 0.491661 |
| MF143338 | RAC&SK-related | | Human | 0.775321 | 1.069043 | 0.491492 |
| MF143350 | RAC&SK-related | | Human | 0.775426 | 1.070752 | 0.491469 |
| MF143362 | RAC&SK-related | | Human | 0.775057 | 1.070575 | 0.491479 |
| MF143367 | RAC&SK-related | | Human | 0.77554 | 1.070176 | 0.491664 |
| MF143382 | RAC&SK-related | | Human | 0.775187 | 1.070668 | 0.491648 |
| MF143387 | RAC&SK-related | | Human | 0.77583 | 1.069391 | 0.491668 |
| MG562535 | RAC&SK-related | | Human | 0.77574 | 1.071644 | 0.491483 |
| MG562552 | RAC&SK-related | | Human | 0.775244 | 1.068665 | 0.491444 |
| MG562555 | RAC&SK-related | | Human | 0.775691 | 1.068651 | 0.491315 |
| MG562560 | RAC&SK-related | | Human | 0.775754 | 1.068771 | 0.491651 |
| MG562564 | RAC&SK-related | | Human | 0.775775 | 1.070423 | 0.491656 |
| MG562567 | RAC&SK-related | | Human | 0.774942 | 1.073137 | 0.491491 |
| MG562572 | RAC&SK-related | | Human | 0.775139 | 1.070325 | 0.491658 |
| MG562586 | RAC&SK-related | | Human | 0.77567 | 1.070322 | 0.491466 |
| MG562605 | RAC&SK-related | | Human | 0.775463 | 1.069787 | 0.491401 |
| MK540658 | RAC&SK-related | | Human | 0.775764 | 1.07176 | 0.491466 |
| MK540668 | RAC&SK-related | | Human | 0.775653 | 1.07033 | 0.491355 |
| MK540669 | RAC&SK-related | | Human | 0.775918 | 1.07146 | 0.491457 |
| MK540677 | RAC&SK-related | | Human | 0.775474 | 1.070521 | 0.491646 |
| MK540702 | RAC&SK-related | | Human | 0.775054 | 1.07148 | 0.491657 |
| MK540720 | RAC&SK-related | | Human | 0.776178 | 1.069666 | 0.491659 |
| MK540723 | RAC&SK-related | | Human | 0.774838 | 1.071581 | 0.491575 |
| MK540745 | RAC&SK-related | | Human | 0.775016 | 1.070921 | 0.491663 |
| MK540747 | RAC&SK-related | | Human | 0.776736 | 1.071362 | 0.491656 |
| MK540749 | RAC&SK-related | | Human | 0.775933 | 1.07049 | 0.491589 |
| MK540761 | RAC&SK-related | | Human | 0.774425 | 1.072133 | 0.491662 |
| MK540796 | RAC&SK-related | | Human | 0.775852 | 1.068649 | 0.491567 |
| MK540799 | RAC&SK-related | | Human | 0.775368 | 1.069788 | 0.491559 |
| MK540826 | RAC&SK-related | | Human | 0.774438 | 1.069029 | 0.491391 |
| MK540853 | RAC&SK-related | | Human | 0.774757 | 1.069504 | 0.491673 |
| MN418144 | RAC&SK-related | | Human | 0.776514 | 1.072475 | 0.491525 |
| MN418168 | RAC&SK-related | | Human | 0.77639 | 1.070339 | 0.491658 |
| MN418172 | RAC&SK-related | | Human | 0.775216 | 1.06951 | 0.491472 |
| ON986457 | RAC&SK-related | | Human | 0.777775 | 1.072906 | 0.491663 |
| ON986465 | RAC&SK-related | | Human | 0.776996 | 1.071854 | 0.491661 |
| ON986474 | RAC&SK-related | | Human | 0.777024 | 1.068443 | 0.491462 |
| JQ685896 | Bat-related | | RAC&SK | 0.784846 | 1.082784 | 0.491608 |
| JQ685897 | Bat-related | | RAC&SK | 0.785736 | 1.078712 | 0.491801 |
| JQ685900 | Bat-related | | RAC&SK | 0.78897 | 1.082259 | 0.491616 |
| JQ685903 | Bat-related | | RAC&SK | 0.784915 | 1.08248 | 0.491781 |
| JQ685906 | Bat-related | | RAC&SK | 0.785781 | 1.080889 | 0.49163 |
| JQ685908 | Bat-related | | RAC&SK | 0.784314 | 1.082974 | 0.491588 |
| JQ685910 | Bat-related | | RAC&SK | 0.786388 | 1.081581 | 0.491514 |
| JQ685911 | Bat-related | | RAC&SK | 0.784845 | 1.080308 | 0.49182 |
| JQ685912 | Bat-related | | RAC&SK | 0.784649 | 1.082822 | 0.491774 |
| JQ685913 | Bat-related | | RAC&SK | 0.784883 | 1.080849 | 0.491819 |
| JQ685914 | Bat-related | | RAC&SK | 0.785153 | 1.081565 | 0.491807 |
| JQ685915 | Bat-related | | RAC&SK | 0.78633 | 1.076213 | 0.491806 |
| JQ685916 | Bat-related | | RAC&SK | 0.784526 | 1.074769 | 0.491535 |
| JQ685918 | Bat-related | | RAC&SK | 0.78237 | 1.079157 | 0.491604 |
| JQ685920 | Bat-related | | RAC&SK | 0.784892 | 1.087493 | 0.491815 |
| JQ685922 | Bat-related | | RAC&SK | 0.789236 | 1.083351 | 0.491613 |
| JQ685925 | Bat-related | | RAC&SK | 0.785898 | 1.087769 | 0.491532 |
| JQ685931 | Bat-related | | RAC&SK | 0.786597 | 1.080408 | 0.491526 |
| JQ685934 | Bat-related | | RAC&SK | 0.78514 | 1.083349 | 0.491602 |
| JQ685935 | Bat-related | | RAC&SK | 0.785163 | 1.080515 | 0.49161 |
| JQ685936 | Bat-related | | RAC&SK | 0.78591 | 1.080797 | 0.491795 |
| JQ685940 | Bat-related | | RAC&SK | 0.784699 | 1.080508 | 0.491587 |
| JQ685942 | Bat-related | | RAC&SK | 0.786973 | 1.086389 | 0.491595 |
| JQ685945 | Bat-related | | RAC&SK | 0.785582 | 1.078219 | 0.491594 |
| JQ685946 | Bat-related | | RAC&SK | 0.787408 | 1.082755 | 0.491631 |
| JQ685949 | Bat-related | | RAC&SK | 0.785215 | 1.080633 | 0.491811 |
| JQ685953 | Bat-related | | RAC&SK | 0.781804 | 1.087727 | 0.49146 |
| JQ685957 | Bat-related | | RAC&SK | 0.783245 | 1.080763 | 0.491798 |
| JQ685959 | Bat-related | | RAC&SK | 0.784545 | 1.080701 | 0.491597 |
| JQ685963 | Bat-related | | RAC&SK | 0.787226 | 1.092101 | 0.491599 |
| JQ685966 | Bat-related | | RAC&SK | 0.785163 | 1.080515 | 0.491559 |
| JQ685971 | Bat-related | | RAC&SK | 0.784719 | 1.090131 | 0.491555 |
| JQ685974 | Bat-related | | RAC&SK | 0.78429 | 1.080839 | 0.491596 |
| KM594024 | Bat-related | | RAC&SK | 0.779638 | 1.07268 | 0.491783 |
| KM594025 | Bat-related | | RAC&SK | 0.785138 | 1.068867 | 0.491482 |
| KM594031 | Bat-related | | RAC&SK | 0.786788 | 1.090395 | 0.491456 |
| KM594032 | Bat-related | | RAC&SK | 0.781298 | 1.077764 | 0.491562 |
| KM594033 | Bat-related | | RAC&SK | 0.787621 | 1.094105 | 0.491699 |
| KM594036 | Bat-related | | RAC&SK | 0.78741 | 1.098314 | 0.491802 |
| KM594038 | Bat-related | | RAC&SK | 0.784965 | 1.085299 | 0.491808 |
| KM594040 | Bat-related | | RAC&SK | 0.783659 | 1.085324 | 0.491803 |
| KM594042 | Bat-related | | RAC&SK | 0.784235 | 1.084517 | 0.491553 |
| KM594043 | Bat-related | | RAC&SK | 0.782893 | 1.088661 | 0.491506 |
| KX148100 | Bat-related | | RAC&SK | 0.784734 | 1.086138 | 0.491608 |
| KX148109 | Bat-related | | RAC&SK | 0.784242 | 1.08784 | 0.491614 |
| KX148268 | Bat-related | | RAC&SK | 0.78573 | 1.088155 | 0.491803 |
| MG458304 | Bat-related | | RAC&SK | 0.785188 | 1.078913 | 0.491789 |
| MK920923 | Bat-related | | RAC&SK | 0.782691 | 1.07867 | 0.491605 |
| OM971001 | Bat-related | | RAC&SK | 0.788181 | 1.091018 | 0.491615 |
| OM971002 | Bat-related | | RAC&SK | 0.787591 | 1.099714 | 0.491817 |
| EF564174 | Dog-related | | RAC&SK | 0.782206 | 1.084327 | 0.491795 |
| EU886631 | Dog-related | | RAC&SK | 0.788621 | 1.086485 | 0.491565 |
| FJ712193 | Dog-related | | RAC&SK | 0.786667 | 1.083041 | 0.491598 |
| FJ866835 | Dog-related | | RAC&SK | 0.787584 | 1.08615 | 0.491615 |
| JX473840 | Dog-related | | RAC&SK | 0.783362 | 1.085623 | 0.491808 |
| KR534217 | Dog-related | | RAC&SK | 0.784527 | 1.086661 | 0.491634 |
| KR906753 | Dog-related | | RAC&SK | 0.781162 | 1.08494 | 0.491609 |
| KR906757 | Dog-related | | RAC&SK | 0.784495 | 1.087911 | 0.491778 |
| KR906763 | Dog-related | | RAC&SK | 0.783848 | 1.082191 | 0.491814 |
| KX036363 | Dog-related | | RAC&SK | 0.786481 | 1.084846 | 0.491794 |
| KX148114 | Dog-related | | RAC&SK | 0.785493 | 1.081074 | 0.491798 |
| KX148211 | Dog-related | | RAC&SK | 0.784672 | 1.093142 | 0.491589 |
| KX148227 | Dog-related | | RAC&SK | 0.779443 | 1.077202 | 0.491515 |
| KX148260 | Dog-related | | RAC&SK | 0.779903 | 1.077213 | 0.491665 |
| KY210225 | Dog-related | | RAC&SK | 0.782872 | 1.085412 | 0.491618 |
| KY210253 | Dog-related | | RAC&SK | 0.783917 | 1.086628 | 0.491792 |
| KY210290 | Dog-related | | RAC&SK | 0.783627 | 1.086143 | 0.491811 |
| KY210303 | Dog-related | | RAC&SK | 0.783513 | 1.086875 | 0.491801 |
| KY210311 | Dog-related | | RAC&SK | 0.781785 | 1.085566 | 0.491521 |
| LC717423 | Dog-related | | RAC&SK | 0.779842 | 1.075871 | 0.491455 |
| LM645024 | Dog-related | | RAC&SK | 0.784868 | 1.083969 | 0.491673 |
| LM645043 | Dog-related | | RAC&SK | 0.78577 | 1.087174 | 0.491806 |
| MF197741 | Dog-related | | RAC&SK | 0.782544 | 1.071526 | 0.491628 |
| MH514972 | Dog-related | | RAC&SK | 0.777491 | 1.067116 | 0.491626 |
| MK540913 | Dog-related | | RAC&SK | 0.786641 | 1.083489 | 0.491803 |
| MK540920 | Dog-related | | RAC&SK | 0.785065 | 1.084234 | 0.491808 |
| MK540960 | Dog-related | | RAC&SK | 0.787379 | 1.085524 | 0.491793 |
| MK540990 | Dog-related | | RAC&SK | 0.786265 | 1.084629 | 0.491608 |
| MK541000 | Dog-related | | RAC&SK | 0.786759 | 1.083747 | 0.491796 |
| MK598383 | Dog-related | | RAC&SK | 0.783023 | 1.084343 | 0.49161 |
| MK760690 | Dog-related | | RAC&SK | 0.784616 | 1.086608 | 0.491557 |
| MN175989 | Dog-related | | RAC&SK | 0.78625 | 1.082868 | 0.491636 |
| MN233910 | Dog-related | | RAC&SK | 0.784682 | 1.086388 | 0.491609 |
| MN233914 | Dog-related | | RAC&SK | 0.785464 | 1.086049 | 0.491796 |
| MN233919 | Dog-related | | RAC&SK | 0.78419 | 1.085058 | 0.491796 |
| MN233936 | Dog-related | | RAC&SK | 0.784999 | 1.085006 | 0.491786 |
| MN233942 | Dog-related | | RAC&SK | 0.784105 | 1.084066 | 0.491803 |
| MN233949 | Dog-related | | RAC&SK | 0.784894 | 1.085985 | 0.491745 |
| MN233962 | Dog-related | | RAC&SK | 0.785486 | 1.085655 | 0.491799 |
| MN233974 | Dog-related | | RAC&SK | 0.78479 | 1.086072 | 0.491608 |
| MN233999 | Dog-related | | RAC&SK | 0.784258 | 1.083987 | 0.491808 |
| MN234029 | Dog-related | | RAC&SK | 0.785142 | 1.086428 | 0.491782 |
| MN234032 | Dog-related | | RAC&SK | 0.78472 | 1.085904 | 0.491813 |
| MN234033 | Dog-related | | RAC&SK | 0.784418 | 1.084212 | 0.491595 |
| MN234053 | Dog-related | | RAC&SK | 0.784357 | 1.082958 | 0.491589 |
| MN726812 | Dog-related | | RAC&SK | 0.782318 | 1.086367 | 0.491596 |
| MN726815 | Dog-related | | RAC&SK | 0.78373 | 1.080806 | 0.491609 |
| MT454632 | Dog-related | | RAC&SK | 0.784653 | 1.084862 | 0.491544 |
| MT454649 | Dog-related | | RAC&SK | 0.784931 | 1.086624 | 0.491558 |
| OM542191 | Dog-related | | RAC&SK | 0.782247 | 1.080229 | 0.49163 |
| JQ685929 | RAC&SK-related | | RAC&SK | 0.789963 | 1.078069 | 0.49156 |
| KY026418 | RAC&SK-related | | RAC&SK | 0.786167 | 1.081168 | 0.491582 |
| KY026441 | RAC&SK-related | | RAC&SK | 0.786156 | 1.080387 | 0.491682 |
| KY026446 | RAC&SK-related | | RAC&SK | 0.785823 | 1.081335 | 0.491604 |
| KY026456 | RAC&SK-related | | RAC&SK | 0.7867 | 1.082448 | 0.49181 |
| KY026472 | RAC&SK-related | | RAC&SK | 0.78621 | 1.080398 | 0.491809 |
| KY026480 | RAC&SK-related | | RAC&SK | 0.787008 | 1.080252 | 0.49151 |
| MF143205 | RAC&SK-related | | RAC&SK | 0.788373 | 1.084742 | 0.491609 |
| MF143237 | RAC&SK-related | | RAC&SK | 0.790322 | 1.084404 | 0.491613 |
| MF143262 | RAC&SK-related | | RAC&SK | 0.786082 | 1.081215 | 0.491604 |
| MF143270 | RAC&SK-related | | RAC&SK | 0.786605 | 1.081316 | 0.491721 |
| MF143320 | RAC&SK-related | | RAC&SK | 0.786486 | 1.082759 | 0.491804 |
| MF143327 | RAC&SK-related | | RAC&SK | 0.786753 | 1.081348 | 0.491612 |
| MF143334 | RAC&SK-related | | RAC&SK | 0.786349 | 1.082172 | 0.491803 |
| MF143338 | RAC&SK-related | | RAC&SK | 0.786037 | 1.08009 | 0.491613 |
| MF143350 | RAC&SK-related | | RAC&SK | 0.786135 | 1.081714 | 0.491597 |
| MF143362 | RAC&SK-related | | RAC&SK | 0.785771 | 1.081232 | 0.491602 |
| MF143367 | RAC&SK-related | | RAC&SK | 0.786274 | 1.08113 | 0.491807 |
| MF143382 | RAC&SK-related | | RAC&SK | 0.78589 | 1.081421 | 0.491793 |
| MF143387 | RAC&SK-related | | RAC&SK | 0.786568 | 1.080493 | 0.491811 |
| MG562535 | RAC&SK-related | | RAC&SK | 0.786438 | 1.083245 | 0.491602 |
| MG562552 | RAC&SK-related | | RAC&SK | 0.78595 | 1.078782 | 0.491583 |
| MG562555 | RAC&SK-related | | RAC&SK | 0.786392 | 1.079608 | 0.491455 |
| MG562560 | RAC&SK-related | | RAC&SK | 0.786491 | 1.079582 | 0.491796 |
| MG562564 | RAC&SK-related | | RAC&SK | 0.786505 | 1.081024 | 0.491798 |
| MG562567 | RAC&SK-related | | RAC&SK | 0.785665 | 1.083661 | 0.491612 |
| MG562572 | RAC&SK-related | | RAC&SK | 0.785839 | 1.08135 | 0.491803 |
| MG562586 | RAC&SK-related | | RAC&SK | 0.786376 | 1.080854 | 0.491592 |
| MG562605 | RAC&SK-related | | RAC&SK | 0.78624 | 1.080219 | 0.491519 |
| MK540658 | RAC&SK-related | | RAC&SK | 0.786531 | 1.082701 | 0.491595 |
| MK540668 | RAC&SK-related | | RAC&SK | 0.786354 | 1.081032 | 0.491494 |
| MK540669 | RAC&SK-related | | RAC&SK | 0.786701 | 1.082614 | 0.491586 |
| MK540677 | RAC&SK-related | | RAC&SK | 0.786205 | 1.081617 | 0.49179 |
| MK540702 | RAC&SK-related | | RAC&SK | 0.785745 | 1.082076 | 0.491801 |
| MK540720 | RAC&SK-related | | RAC&SK | 0.786929 | 1.080219 | 0.491802 |
| MK540723 | RAC&SK-related | | RAC&SK | 0.785541 | 1.08219 | 0.4917 |
| MK540745 | RAC&SK-related | | RAC&SK | 0.785722 | 1.081674 | 0.491807 |
| MK540747 | RAC&SK-related | | RAC&SK | 0.787457 | 1.082285 | 0.491799 |
| MK540749 | RAC&SK-related | | RAC&SK | 0.786686 | 1.081434 | 0.491707 |
| MK540761 | RAC&SK-related | | RAC&SK | 0.785118 | 1.083139 | 0.491805 |
| MK540796 | RAC&SK-related | | RAC&SK | 0.786617 | 1.07998 | 0.491698 |
| MK540799 | RAC&SK-related | | RAC&SK | 0.786161 | 1.080336 | 0.491698 |
| MK540826 | RAC&SK-related | | RAC&SK | 0.785232 | 1.079531 | 0.491521 |
| MK540853 | RAC&SK-related | | RAC&SK | 0.785529 | 1.080262 | 0.491799 |
| MN418144 | RAC&SK-related | | RAC&SK | 0.7872 | 1.083604 | 0.491654 |
| MN418168 | RAC&SK-related | | RAC&SK | 0.787121 | 1.081698 | 0.491802 |
| MN418172 | RAC&SK-related | | RAC&SK | 0.786011 | 1.079674 | 0.4916 |
| ON986457 | RAC&SK-related | | RAC&SK | 0.78858 | 1.084457 | 0.491808 |
| ON986465 | RAC&SK-related | | RAC&SK | 0.787825 | 1.083116 | 0.491803 |
| ON986474 | RAC&SK-related | | RAC&SK | 0.787868 | 1.079989 | 0.491597 |
